# Synthesis of 5-(pyrazol-4-yl) pentanoic acids and 4-(pyrazol-4-yl) butanoic acids *via* a cascade annulation/ring-opening reaction between hydrazone and dienone†

**DOI:** 10.1039/d5ra03561a

**Published:** 2025-07-10

**Authors:** Kalinga H. Nayak, Robert K. Jijin, Beneesh P. Babu

**Affiliations:** a Department of Chemistry, National Institute of Technology Karnataka Surathkal Mangalore 575025 India pbbeneesh@nitk.edu.in

## Abstract

Herein, we report an interesting [3 + 2] annulation/ring-opening cascade reaction between hydrazones and exocyclic dienones *via* an aerobic, copper(ii) catalysis to synthesize 5-(pyrazol-4-yl) pentanoic acid and 4-(pyrazol-4-yl) butanoic acid derivatives. The annulation first affords a spiro pyrazoline with unprecedented regiochemistry, followed by a cascade nucleophilic ring opening by water to yield pyrazolyl pentanoic and butanoic acid derivatives in good yield. Broad substrate scope, inexpensive and green catalyst and oxidant, and relatively mild reaction conditions enhance the versatility of this protocol.

## Abstract 2

A copper(ii) catalyzed synthesis of 5-(pyrazol-4-yl) pentanoic acid and 4-(pyrazol-4-yl) butanoic acid derivatives *via* an aerobic [3 + 2] annulation/ring-opening cascade pathway is described.

## Graphical abstract

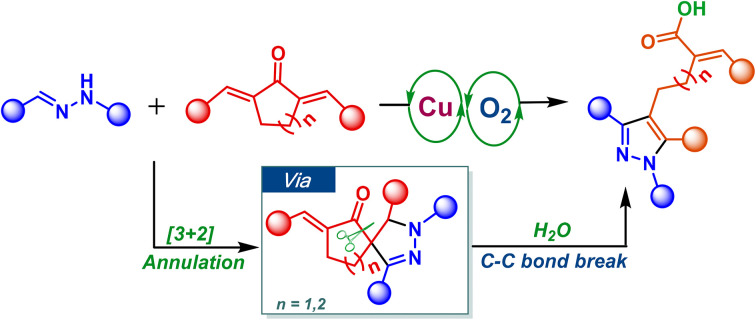

## Introduction

Synthetic tools to access small organic molecules and their libraries with diversity, in a rapid and selective manner, are one of the most prominent research focuses. Heterocycles and their derivatives hold a major share of such molecular libraries due to their widespread presence in molecules ranging from drugs and pharmaceuticals to agrochemicals, chromophores, dyes, *etc.*^1^ However, environmental demands and financial constraints always challenge synthetic organic chemists to devise new and improved protocols.^2^ Traditionally, the C–C bond formation is considered the backbone of synthetic organic chemistry. Nevertheless, the same C–C sigma bond cleavage has emerged as a potential organic synthesis strategy in the past decade.^3^ The abundant and robust C–C bond cleavage could make unconventional transformations feasible, generating complexity and skeletal changes that otherwise would have been impossible to execute.^4^ The cleavage of the C–C sigma bond is a high-energy process due to the stability of C–C bonds, and the literature reports in this direction are predominantly catalyzed by transition metals such as Pd, Ru, Rh, and Ir.^5^ Despite the high-energy barrier associated with the C–C bond cleavage, such reactivity is common in cyclic molecules, especially among spiro compounds. The driving force behind such a pathway is mainly the release in the ring strain accompanying the ring system.^6^

**Fig. 1:**
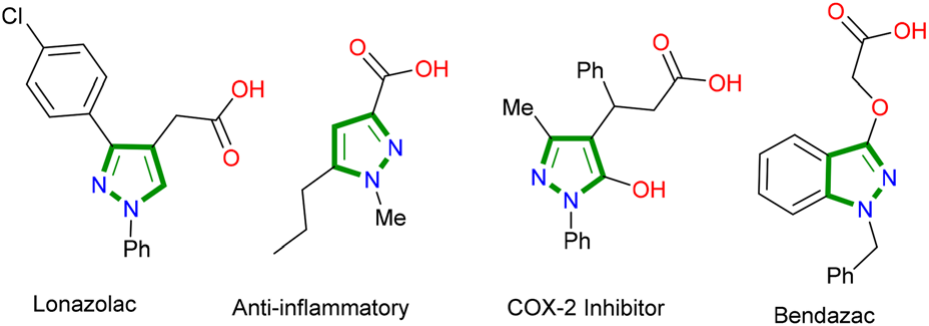
Examples of some drug molecules containing pyrazolyl carboxylic acids.

Pyrazole is an important class of five-membered heterocycle from the azole family and constitutes the core unit of many commercially available drug molecules.^7^ Heterocyclic carboxylic acids hold a special place among the bioactive molecules, and many commercial drug molecules contain this scaffold.^8^ It is reported that nearly 400+ marketed drug molecules contain at least one free carboxylic acid moiety. Pyrazole carboxylic acids are not an exception and can be found in several bioactive molecules and drugs (Fig. 1).^9^ The free acid group can respond to the pH variations very effectively and provide an active site for strong electrostatic interaction and non-covalent interactions such as hydrogen bonding.^10^

**Scheme 1:**
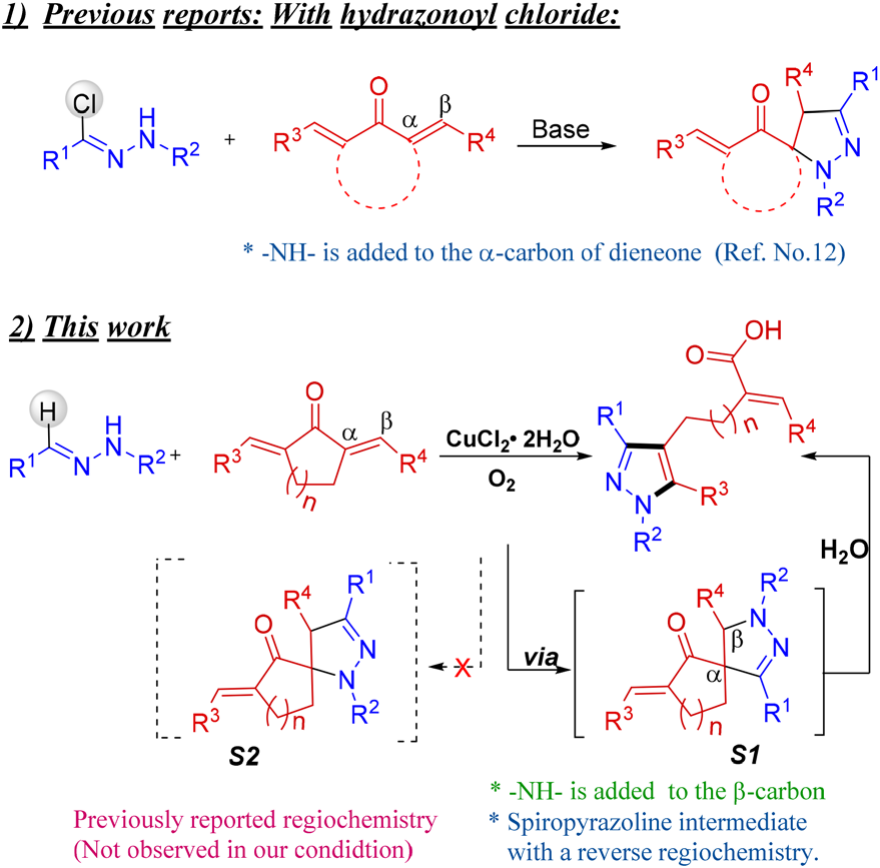
Reaction between hydrazone and exocyclic dienone.

In light of our recent reports on the [3 + 2] annulation reactions of aldehyde hydrazones across various enones and dienones,^11^ we were curious to expand the scope of this [3 + 2] annulation reaction further to cyclic dienones such as dibenzylidenecyclohexanone containing exocyclic double bonds. The [3 + 2] annulation between exocyclic dienones and hydrazonoyl chlorides, resulting in spiro pyrazolines *via* nitrile imine intermediate, had already been documented.^12^ However, the corresponding reactivity of simple hydrazones has not been explored. In comparison, the hydrazones are ideal substrates for such reactions as they avoid the extra halogenation step to prepare hydrazonoyl chloride. In this report, we disclose an interesting reaction between hydrazones and dibenzylidene cycloalkanones. Unlike the expected spiro pyrazoline derivative, an unprecedented ring-opening pathway prevailed after the annulation, and pyrazolyl pentanoic/butanoic acid was obtained exclusively by a cascade reaction. Explicit characterization of the final product, including a single crystal X-ray analysis, confirmed that the regiochemistry of the annulation of hydrazone with dienone is opposite to that reported earlier between hydrazonoyl chloride and dienone. The spiro pyrazoline intermediate formed is an all-carbon-bonded spiro carbon (structure S1, Scheme 1) in contrast to the previously reported spiro pyrazolines with hydrazonoyl chloride, where one bond of the spiro carbon was a C–N bond (structure S2, Scheme 1).^12^ Further, the unexpected C–C bond cleavage of the spiro pyrazoline, under the optimized conditions, is driven by the presence of the Lewis acid Cu(ii) salt and the increased stability of the final product due to the release of ring strain and decreased steric effect.

## Results and discussion

In the pilot experiment, the cyclic dienone, 2,6-dibenzylidenecyclohexan-1-one 2a was treated with benzaldehyde hydrazone 1a. No reaction was observed either at room temperature or on heating in dichloroethane under an oxygen atmosphere without any catalyst or additive. Interestingly, as observed in the previous studies, a new product was formed in low yield in the presence of catalytic CuCl_2_·2H_2_O (50 mol%) at 80 °C. The product was later characterized unambiguously as a pyrazole-bound pentanoic acid 3aa, contrary to the expected spiro pyrazoline. An unprecedented ring-opening reaction of the expected spiro pyrazoline derivative led to the formation of the new product. As mentioned above, the regiochemistry of the annulation between the hydrazone and 2a is opposite to that reported with hydrazonoyl chloride and dienones. The amine –NH– of the hydrazone is regioselectively added to the β-carbon of the exocyclic dienone (structure S1, Scheme 1) in contrast to the amine –NH– of hydrazonoyl chloride that is added to the α-carbon of the exocyclic enone affording spiro pyrazolines of S2-type connectivity (Scheme 1). The spiropyrazoline subsequently opened up by water to yield pyrazolyl pentanoic acid (Scheme 1).

Encouraged by this unexpected yet new reaction pathway, we optimized the reaction conditions by systematically varying the parameters one by one, and the observations are summarized in Table 1. While screening the solvents of different polarities and boiling points, acetonitrile was identified as the best solvent, offering a 76% product yield. Further, optimization trials to choose the best Lewis acids were performed using various salts such as AlCl_3_, Mg(OTf)_2_, Ca(OTf)_2_, Zn(OTf)_2_, Cu(OTf)_2_, FeCl_3_·4H_2_O and anhydrous FeCl_3_. Nevertheless, all salts were less effective than CuCl_2_·2H_2_O. After these extensive screenings, the most favourable condition for the reaction was identified as heating 1a and 2a in acetonitrile at 80 °C for 28 h under an oxygen atmosphere (1 atm.) in the presence of the catalyst CuCl_2_·2H_2_O (50 mol%). Interestingly, when the reaction was repeated with anhydrous CuCl_2_ under the optimized conditions, the spiro pyrazoline 3′aa (Scheme 5) was obtained exclusively, confirming the role of water in the ring-opening step. The difference in the regiochemistry of the product was confirmed by ^1^H NMR, ^13^C NMR, and DEPT-135 spectra explicitly (please see ESI for details†).

**Table 1:** Optimization of the reaction conditions^a^

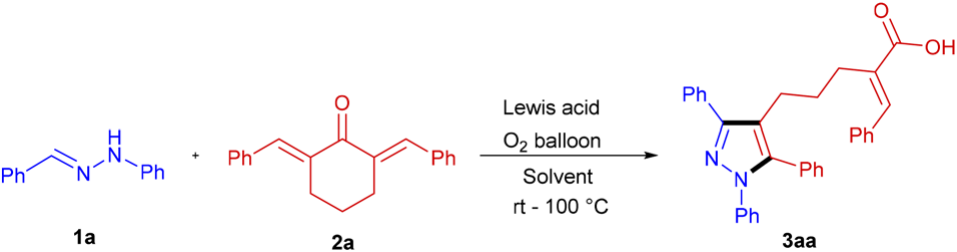				
SI no.	Lewis acid	Solvent	Temp. (°C)	Yield^b^ (%)
1	—	Dichloroethane	rt	nd
2	—	Dichloroethane	80	nd
3	CuCl_2_·2H_2_O	Dichloroethane	80	30
4	CuCl_2_·2H_2_O	DMSO	100	nd
5	CuCl_2_·2H_2_O	Chlorobenzene	90	20
6	CuCl_2_·2H_2_O	CCl_4_	70	15
7	CuCl_2_·2H_2_O	Benzene	80	nd
8	FeCl_3_	Dichloroethane	80	nd
9	CuCl_2_·2H_2_O	Ethanol	80	nd
10	CuCl_2_·2H_2_O	Acetonitrile	80	76
11	Cu (OTf)_2_	Acetonitrile	80	nd
12	Mg (OTf)_2_	Acetonitrile	80	nd
13	Ca (OTf)_2_	Acetonitrile	80	nd
14	Zn (OTf)_2_	Acetonitrile	80	nd
15	AlCl_3_	Acetonitrile	80	nd
16	CuCl_2_	Acetonitrile	80	nd^c^
17	FeCl_3_·4H_2_O	Acetonitrile	80	nd

a Reaction conditions: 1a (0.6 mmol), 2a (0.5 mmol), and Lewis acid (50 mol%) in solvent (5 mL) at 80 °C under O_2_ atmosphere, 28 h.

b Isolated yield, nd = not detected.

c Spiro pyrazoline was formed exclusively.

**Scheme 2:**
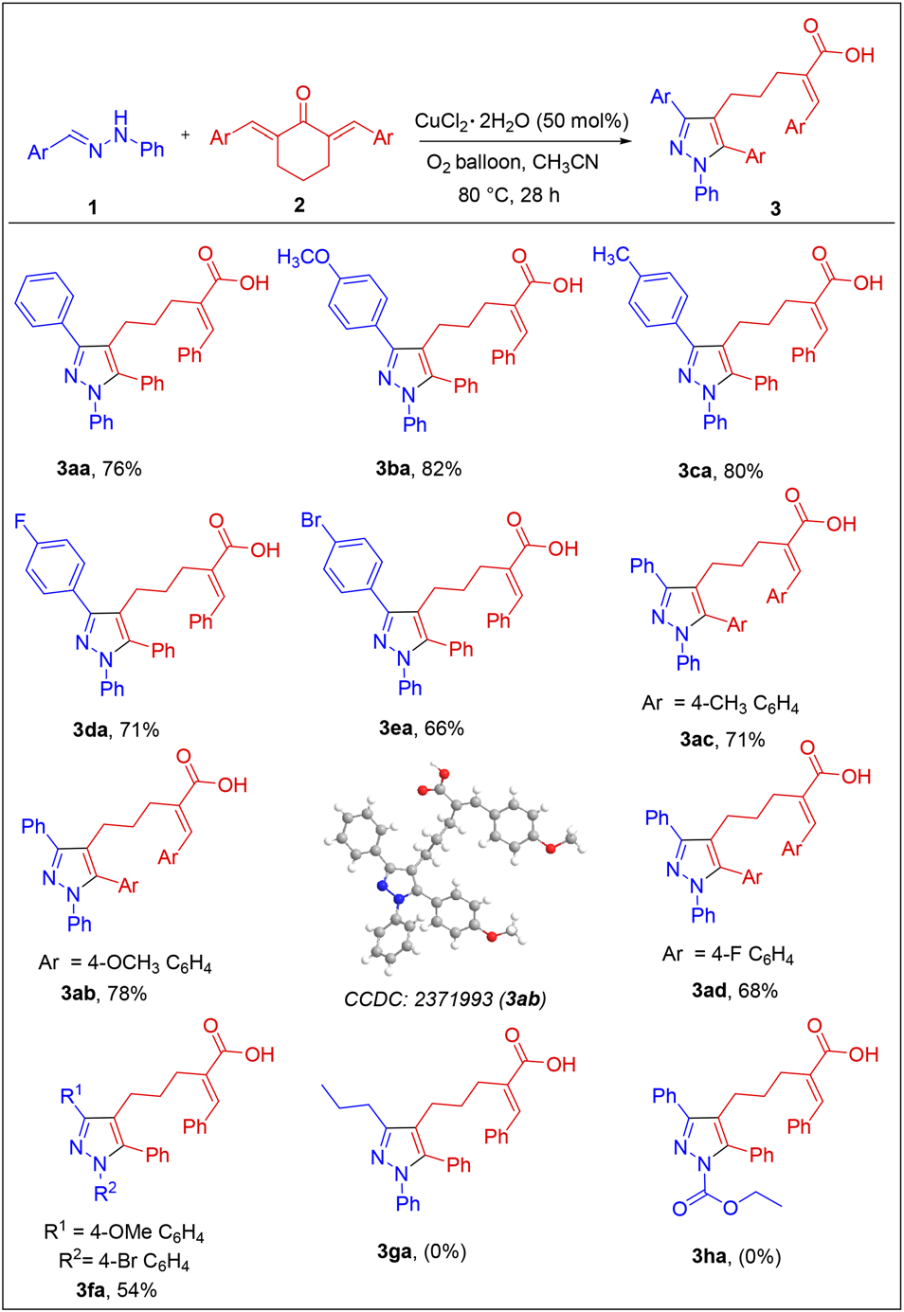
Substrate-scope of hydrazones and six-membered dienones. Reaction conditions: 1a (0.6 mmol), 2a (0.5 mmol), CuCl_2_·2H_2_O (50 mol%), acetonitrile (5 mL), at 80 °C under O_2_ atmosphere, 28 h. Isolated yields are reported.

With the optimized reaction conditions, we next examined the compatibility of this synthetic route using hydrazones and cyclic dienones of different electronic natures (Scheme 2) and the results are summarized in Scheme 2. Hydrazones prepared from aldehydes of different electronic natures readily reacted with 2a under the optimized conditions to afford the pyrazolyl acids in moderate to good yield (3aa–3fa). Similarly, analogs of 2a were prepared from cyclohexanone and various aldehydes and later treated with 1a, under the standard conditions. As expected, the products were isolated in good yields (3ab–3ad) expanding the versatility of the protocol. So, the reaction is highly flexible as the functional groups of different electronic natures are well tolerated on either side of the substrates, thereby expanding the diversity further. Further, the derivative 3ab was characterized unambiguously by single-crystal analysis (CCDC no.: 2371993). However, it was noticed that substrates with more electron-dense substituents performed slightly better in the reaction. Furthermore, the hydrazones prepared from aliphatic aldehydes failed to offer the product 3ga due to the ready hydrolysis of such hydrazones under the optimized reaction conditions. The hydrazone prepared from benzaldehyde and ethyl carbazate was also unsuccessful.

**Scheme 3:**
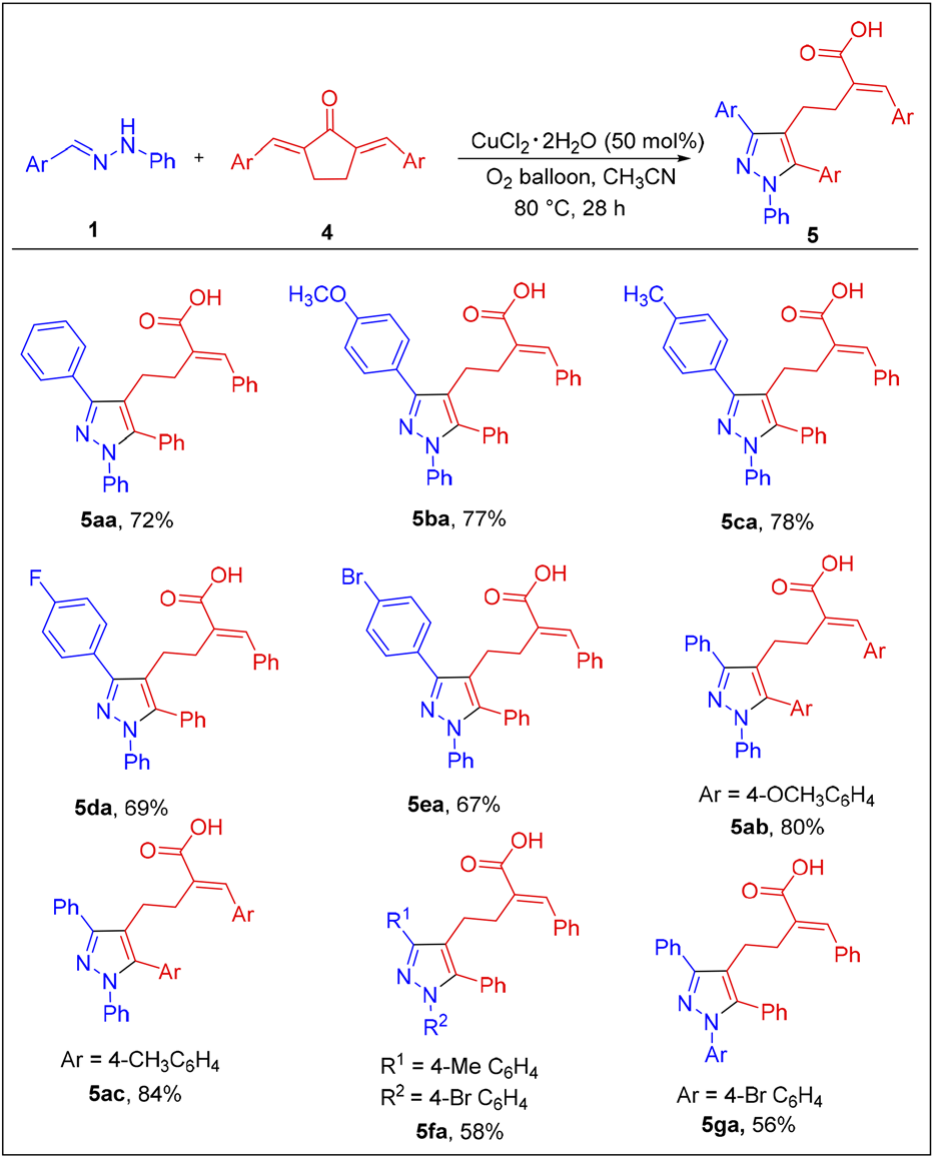
Substrate scope of hydrazones and five-membered dienones. Reaction conditions: 1a (0.6 mmol), 4a (0.5 mmol), CuCl_2_·2H_2_O (50 mol%), acetonitrile (5 mL), at 80 °C under O_2_ atmosphere, 28 h. Isolated yields are reported.

**Scheme 4:**
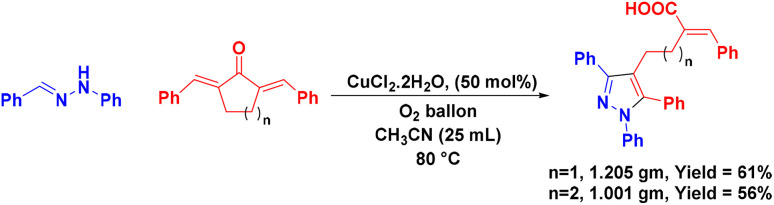
Gram-scale synthesis.

Subsequently, we focused on expanding the reaction scope to 2,5-dibenzylidene cyclopentane-1-one 4a. Various aldehyde hydrazones were treated with 4a under the optimized conditions, and the results are summarized in Scheme 3. As expected, the [3 + 2] annulation followed by the ring-opening pathway was compatible with 4a, and the expected pyrazolyl butanoic acid derivatives were isolated in moderate to good yield. As is evident from Scheme 3, the reaction was quite flexible with substrates of various electronic natures on either substrate and the results were also comparable. However, aliphatic hydrazones were unsuccessful in the reaction as observed before. Further, the scalability of the reaction was checked by performing the reaction between 1a (4.44 mmol) and 2a (3.7 mmol) under the optimized conditions, and the product 3aa was isolated as a pale-yellow solid in 56% yield, 1.001 gm (Scheme 4). The scalability of the reaction was extended further to synthesize pyrazolyl butanoic acid (5aa) derivative, and the final product was collected in around 61% yield.

**Scheme 5:**
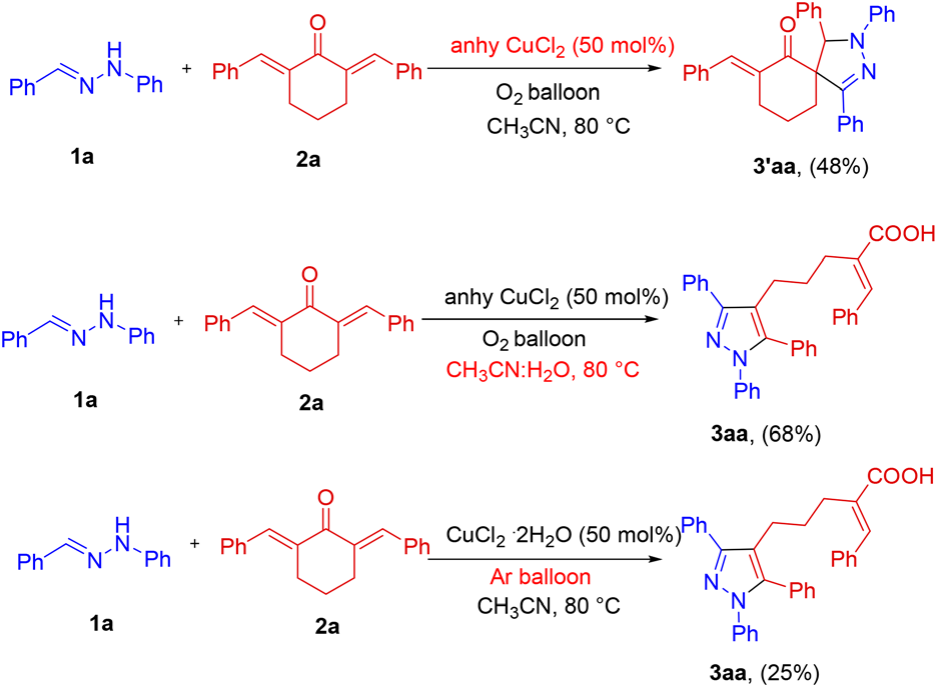
Control experiments.

Control experiments were performed to gain insight into the reaction pathway and are summarized in Scheme 5. Interestingly, when anhydrous CuCl_2_ is used in place of CuCl_2_·2H_2_O, the intermediate spiro pyrazoline 3′aa formation was observed instead of 3aa. Nevertheless, the expected 3aa was formed exclusively when the reaction was repeated in a mixture of CH_3_CN : H_2_O (9 : 1) with anhydrous CuCl_2_. These observations suggest that the water molecules of the hydrated CuCl_2_ salt trigger the ring opening and the presence of water is essential, either in the catalyst or solvent, to drive the reaction to the ring opening direction.^13*a*^ To the best of our knowledge, this is a very rare observation in which a mild nucleophile, such as water, opens up an all-carbon spiro ketone at ambient reaction conditions in a selective manner. When the reaction was repeated under an inert argon atmosphere, the conversion was very low indicating the involvement of a copper-catalyzed aerobic oxidation step converting dihydropyrazole to the pyrazole core.

**Scheme 6:**
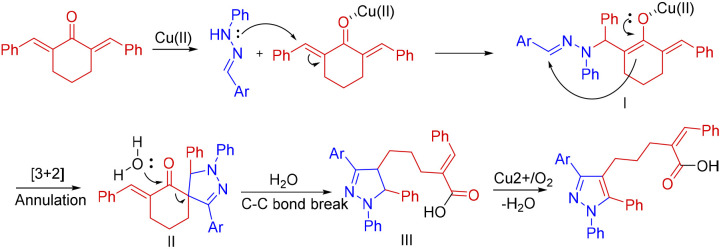
Plausible reaction mechanism.

Based on the control experiments and previous literature reports,^11^ a possible reaction mechanism was proposed for this [3 + 2] annulation/ring-opening reaction (Scheme 6). The Lewis acidity of the copper chloride first facilitates the addition of the mild nucleophile hydrazone to the dienone. The regiochemistry of this addition is opposite to what has already been reported with hydrazonoyl chloride. The coordination of the carbonyl oxygen to the Cu(ii) will likely render the β-carbon more electrophilic, directing the hydrazone –NH– towards it, forming the intermediate I. This nucleophilic addition is followed by [3 + 2] annulation to form the spiro pyrazoline II. This intermediate spiro pyrazoline II further underwent nucleophilic ring opening by water to afford the dihydropyrazole derivative III. The final product is formed by the copper-catalyzed aerobic oxidation of the intermediate III.^11*a*,13*b*,*c*^

## Conclusions

In conclusion, we have disclosed an interesting reaction that allows the synthesis of pyrazolyl pentanoic/butanoic acid derivatives *via* an aerobic, copper-catalyzed, annulation/ring-opening pathway. Abundant, stable, and cost-effective substrates such as hydrazones and exocyclic dienones are on board at ambient conditions. Notably, this regioselective protocol showcases two novel observations in one spot. First, the regiochemistry of the addition of hydrazone to exocyclic dienone is opposite to that reported with similar hydrazone analogs. Surprisingly, the [3 + 2] annulation was further followed by an unprecedented ring opening by water, enabling easy access to highly challenging scaffolds such as pyrazole-bound pentanoic and butanoic acid derivatives in one pot. Green and cost-effective reagents and catalysts and ambient reaction conditions, which are free of any expensive substrates or additives, are particularly noteworthy. Attempts to expand the synthetic potential of this protocol further to access more challenging scaffolds are underway.

## Conflicts of interest

There are no conflicts to declare.

## Supplementary Material

RA-015-D5RA03561A-s001

RA-015-D5RA03561A-s002

## Data Availability

The data supporting this article have been included as part of the ESI.† It contains experimental details, copies of the ^1^H NMR and ^13^C NMR spectra of all the newly synthesized compounds, and single-crystal X-ray data of compound 3ab (CCDC no. 2371993).
