# Supplementary material for: Synthesis of 5-(pyrazol-4-yl) pentanoic acids and 4-(pyrazol-4-yl) butanoic acids via a cascade annulation/ring-opening reaction between hydrazone and dienone
Source: RSC Adv. 2025 Jul 10;15(30):24137–41. doi: 10.1039/d5ra03561a (PMC12242993; doi:10.1039/d5ra03561a)

Supplementary Information (SI) for RSC Advances.
This journal is © The Royal Society of Chemistry 2025

Synthesis of 5-(Pyrazol-4-yl) Pentanoic Acids and 4-(Pyrazol-4-yl) Butanoic Acids via a Cascade Annulation/Ring-Opening Reaction between Hydrazone and Dienone

Kalinga H. Nayak, Robert K. Jijin and Beneesh P. Babu*

Department of Chemistry, National Institute of Technology, Karnataka

Surathkal, Mangalore, INDIA – 575025 E-mail: pbbeneesh@nitk.edu.in

Table of contents

**1.** General information and procedures		
1.1	General procedure for the synthesis of hydrazones	S1
1.2	General procedure for the synthesis of dienone derivatives	S2
1.3	Mechanistic Investigations	S2
1.4	Single Crystal X-ray Data of the Compound 3ab	S6
1.5	General procedure for the synthesis of pyrazole acid derivatives:	S8
1.6	Characterization data of all the newly synthesized compounds	S8
1.7	Procedure for control experiments	S16
1.8	Procedure for Gram-scale synthesis	S17
1.9	References	S18
2.0	^{1}H and $^{13}C\{^{1}H\}$ NMR spectral images of all new compounds	S19

1. General information and procedures

Unless otherwise stated, all reactions were carried out in a 25 mL round-bottom flask. All the reagents were bought from commercial suppliers and used without additional purification. The crude reaction mixture was purified by silica gel (100-200 mesh) column chromatography using a pet ether-ethyl acetate solvent mixture as the eluent. The isolated compounds were characterized by ^{1}H and ^{13}C NMR spectroscopy, Infrared spectroscopy, and High-Resolution Mass Spectrometry (HRMS).

Melting points of the solid samples were determined using the Stuart melting point apparatus. Other characterizations such as ^{1}H NMR and ^{13}C NMR spectra were recorded in $CDCl_3$/ DMSO-d_6 on Bruker Ascend^{TM} 400 MHz spectrometer with tetramethyl silane (TMS; δ H = 0 ppm) as an internal standard and chemical shifts were reported in ppm relative to TMS. The resonance multiplicity is described as s (singlet), d (doublet), t (triplet), m (multiplet), dd (doublet of doublets), and q (quartet). Fourier transform infrared (FTIR) spectra using ATR technique on a Bruker Alpha 400 FTIR spectrometer equipped with silicon carbide as an IR source and only intense peaks were reported. HRMS were recorded on a Waters-Xevo G2-XS-QtoF and The ACQUITY^{TM} UPLC^{TM} H-Class PLUS Bio system mass spectrometer using the ESI method with an orbitrap mass analyzer.

1.1 General procedure for the synthesis of hydrazones:

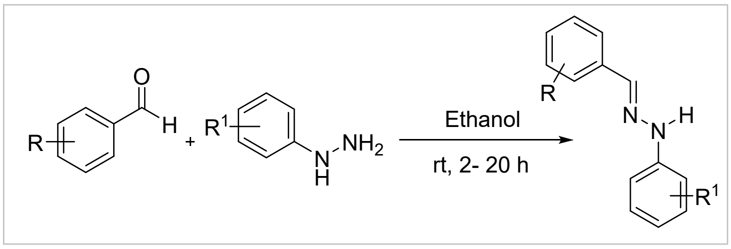

Scheme S1. Synthesis of hydrazone

Phenyl hydrazine (1.08 g, 1 equiv.) in ethanol (10 mL), was stirred in a round bottom flask at room temperature. To the stirring solution, the corresponding aldehyde (1.06 g, 10 mmol) was added (solid aldehyde was added portion-wise and liquid aldehyde dropwise) and stirred the mixture for about 8-20 hours (depending on the electronic nature of aldehydes). The progress of the reaction was monitored by TLC. After the completion of the reaction, the mixture was poured into ice-cold water. The precipitate formed was filtered off and washed with ice-cold water, followed by a pet ether. The solid mass obtained was dissolved in

dichloromethane and dried using Na_2SO_4. The solvent was evaporated under a vacuum and the obtained product was used for all other reactions.

1.2 General procedure for the synthesis of dienone:

NaOH (2.0 g, 5 equiv.) in 10 mL water was added dropwise to 20 mL of ethanol in a round bottom flask followed by cyclohexanone (981mg, 10 mmol) and the benzaldehyde (2.12 g, 20.0 mmol) (solid aldehyde was added portion-wise and liquid aldehyde dropwise). The progress of the reaction was monitored by TLC. After the completion of the reaction, the mixture was poured into ice-cold water. The precipitate formed was filtered off and washed with ice-cold water, followed by a pet ether. The solid mass obtained was dissolved in dichloromethane and dried using Na_2SO_4. The solvent was evaporated under a vacuum and the obtained product was used for all other reactions.

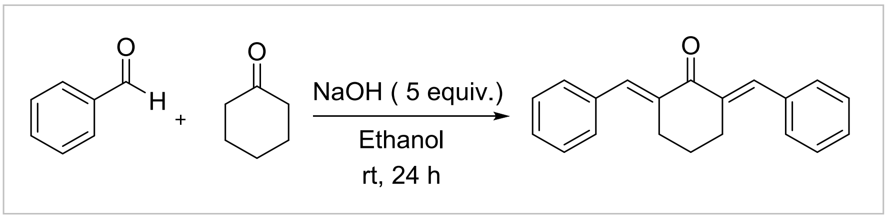

Scheme S2. Synthesis of dienone

1.3 Mechanistic Investigations

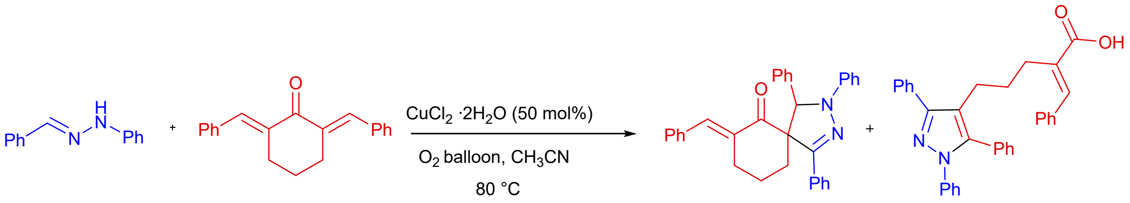

Scheme S3. Pilot experiment between **1a** and **2a**

To an oven-dried 25 mL round bottom flask equipped with a magnetic stirrer, 1-benzylidene-2-phenylhydrazine, **1a** (0.6 mmol, 1.2 equiv.), 2,6-di((E)-benzylidene) cyclohexan-1-one, **2a** (0.5 mmol) and $CuCl_2 \cdot 2H_2O$ (0.25 mmol, 0.5 equiv.) were weighed and added followed by 5 mL of acetonitrile solvent. The reaction vessel was stirred in an oil bath at 80 °C under an oxygen atmosphere. The progress of the reaction was monitored by TLC as shown below. After 9h, the reaction predominantly yielded the 7-benzylidene-1,3,4-triphenyl-2,3-diazaspiro [4.5] dec-1-en-6-one derivative, **3'aa** as shown below. After 20h, the

TLC showed a mixture of both 2-benzylidene-5-(1,3,5-triphenyl-1H-pyrazol-4-yl) pentanoic acid, **3aa** and the 7-benzylidene-1,3,4-triphenyl-2,3-diazaspiro [4.5] dec-1-en-6-one derivatives, **3’aa**. After 28h, the reaction exclusively offered only the 2-benzylidene-5-(1,3,5-triphenyl-1H-pyrazol-4-yl) pentanoic acid derivative, **3aa** (Fig 1). The reaction mixture was cooled to room temperature, diluted with ethyl acetate, and washed with water. The organic layer was concentrated, and the residue was purified by silica gel column chromatography using pet ether-ethyl acetate (hexane/EtOAc, 8:2) as eluent.

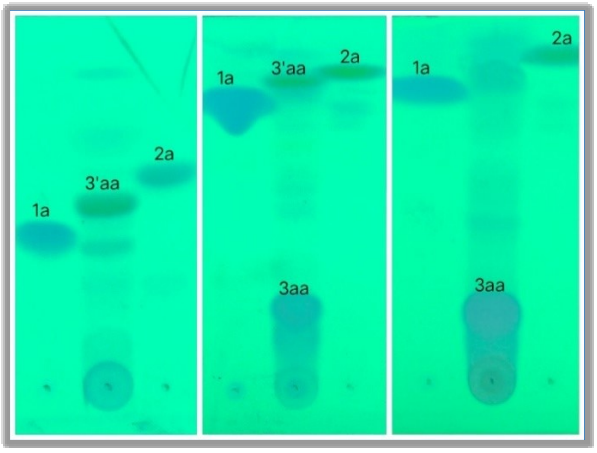

9 h 20 h 28 h

Figure 1: Progress of the reaction between **1a** and **2a** monitored by thin layer chromatography.

The spiro pyrazoline intermediate (**3’aa**) formed was characterized exclusively by ^{1}H,^{13}C, DEPT-135 NMR analysis, (pages S24, S25, and S26) and mass spectrometry, and the observed chemical shift value of the spiro carbon (δ=66.4 ppm) suggested the formation of an all-carbon-bonded spiro carbon (Figure 2) in contrast to the previously reported spiro pyrazolines with hydrazonoyl chlorides (Figure 3) where one bond of the spiro carbon was a C-N bond with a δ value of 80 ppm. The chemical shift value of the benzylic H close to the spiro centre, δ = 5.5 ppm, also validates the proposed structure. Finally, the structure of **3aa** was confirmed unambiguously by single-crystal X-ray analysis.

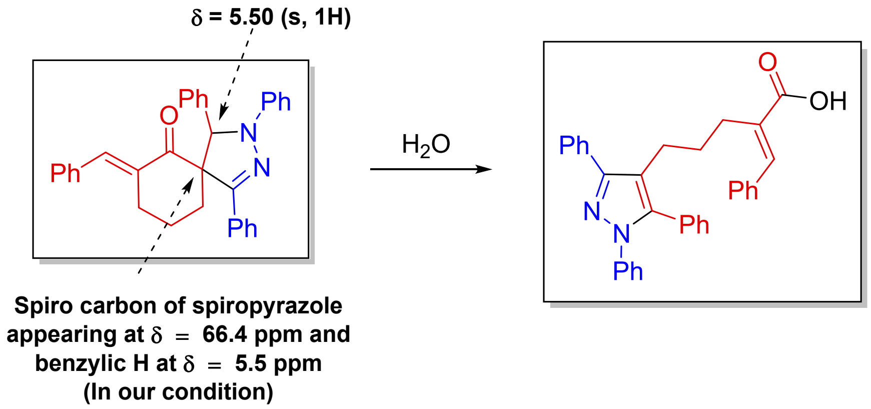

Figure 2: ^{1}H and ^{13}C NMR chemical shift value of spiro compound **3'aa** obtained with hydrazone

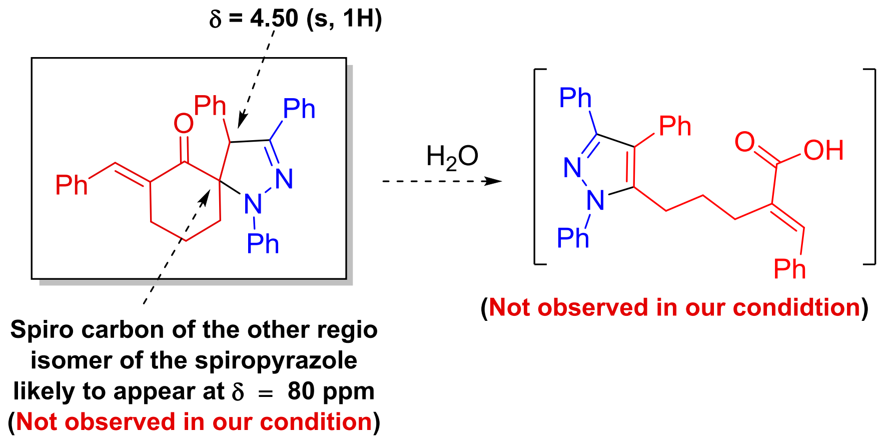

Figure 3: ^{1}H and ^{13}C NMR chemical shift values of the other regio- isomer of the spiropyrazoline reported with hydrazonoyl chloride.[S3]

The progress of the reaction between **1a** and **2a** under the optimized conditions was followed by live HRMS recording. A portion of the reaction mixture was withdrawn halfway through the reaction, that is, after 14h, and subjected to HRMS analysis, which clearly confirmed that the spiro pyrazoline, **3'aa**, and the ring-opened pyrazolyl pentanoic acid, **3aa**, coexist in the reaction mixture (figures 4,5,6). However, on prolonged heating, after 28h, the reaction afforded only **3aa** exclusively. We also isolated the spiro pyrazoline intermediate via column chromatography and characterized it exclusively using ^{1}H, ^{13}C, DEPT-135, and HRMS spectra. Please refer the spectra session for details.

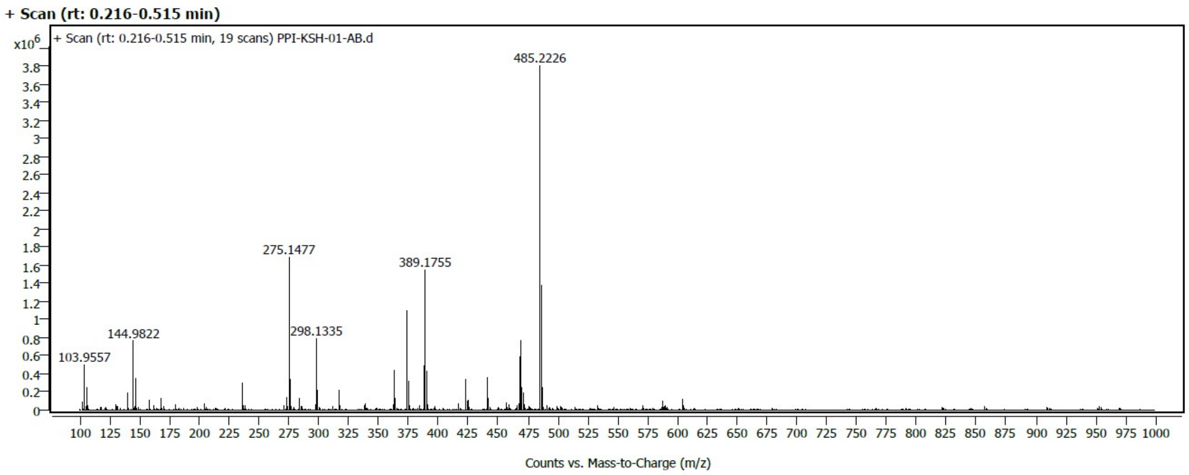

Figure 4: HRMS Data of reaction mixture between **1a** and **2a**

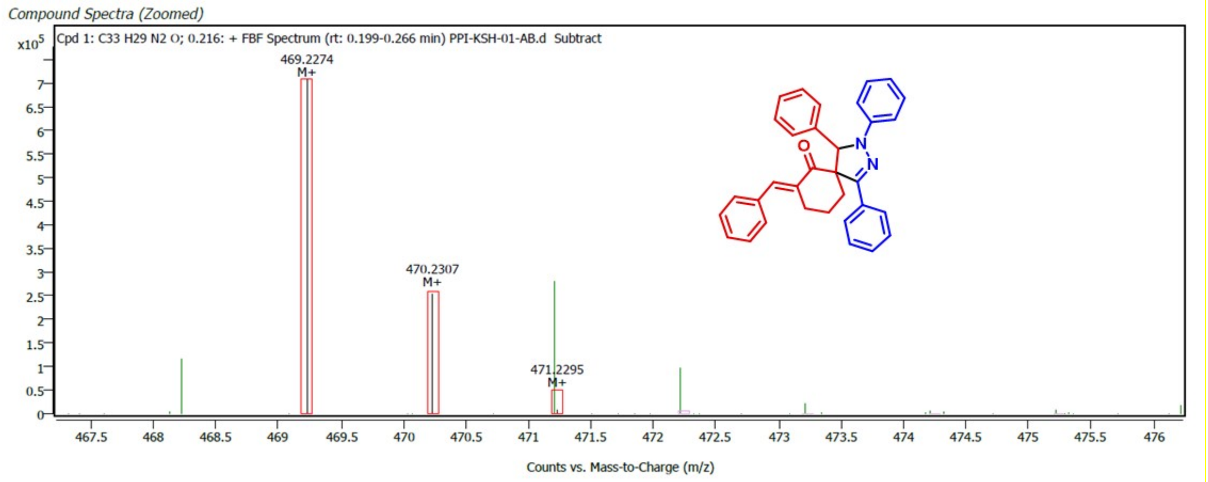

Figure 5: HRMS (ESI-TOF) m/z: $[M+H]^+$ calcd for $C_{33}H_{29}N_2O$ 469.2274; found 469.2274.

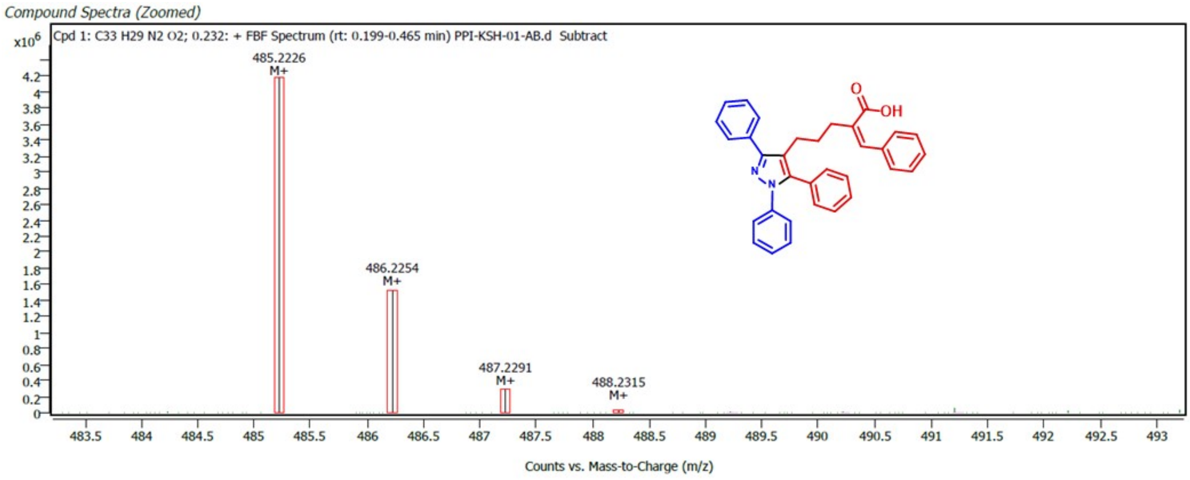

Figure 6: HRMS (ESI-TOF) m/z: $[M+H]^+$ calcd for $C_{33}H_{29}N_2O_2$ 485.2224; found 485.2226.

1.4 Single Crystal X-ray Data of the Compound 3ab:

Compound 3ab:

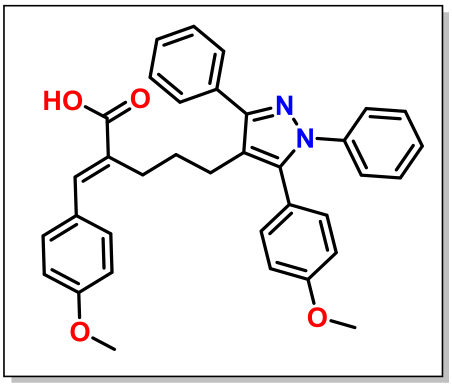

CCDC Number: 2371993

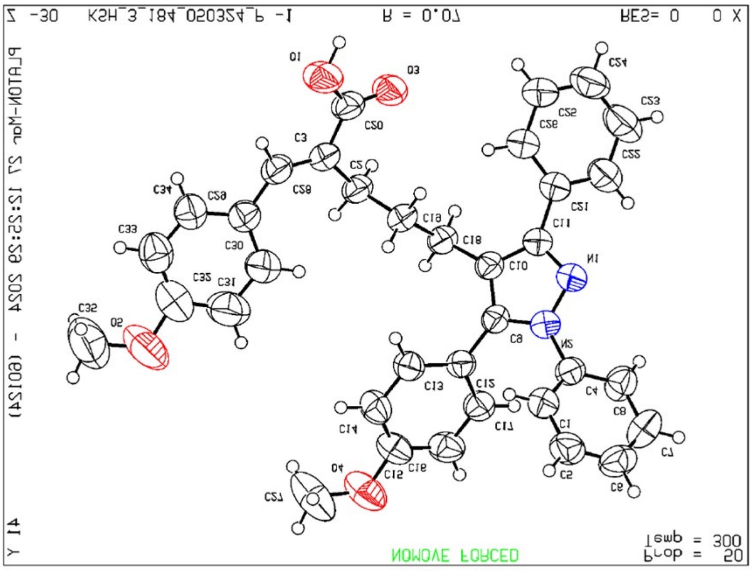

Figure 7: ORTEP diagram of molecule **3ab**. Displacement ellipsoids are drawn at the 50% probability level.

Table 1. Sample and crystal data.

Identification code	**3ab**	
Chemical formula	$C_{35}H_{32}N_2O_4$	
Formula weight	544.62 g/mol	
Temperature	299(2) K	
Wavelength	0.71073 Å	
Crystal size	0.111 x 0.178 x 0.230 mm	
Crystal habit	white block	
Crystal system	triclinic	
Space group	P -1	
Unit cell dimensions	a = 8.7430(5) Å	α = 74.888(2)°
	b = 11.8023(6) Å	β = 83.416(2)°
	c = 15.3997(8) Å	γ = 74.210(2)°
Volume	1474.58(14) Å^3	

Z	2
Density (calculated)	1.227 g/cm^3
Absorption coefficient	0.080 mm^{-1}
F(000)	576

Table 2. Data collection and structure refinement.

Theta range for data collection	2.02 to 28.35°	
Index ranges	-11<=h<=11, -15<=k<=15, -20<=l<=20	
Reflections collected	36504	
Independent reflections	7347 [R(int) = 0.0616]	
Coverage of independent reflections	99.7%	
Absorption correction	Multi-Scan	
Max. and min. transmission	0.9910 and 0.9820	
Structure solution technique	direct methods	
Structure solution program	XT, VERSION 2018/2	
Refinement method	Full-matrix least-squares on F^2	
Refinement program	SHELXL-2019/1 (Sheldrick, 2019)	
Function minimized	Σ w (F_o^2 - F_c^2)^2	
Data/restraints/parameters	7347 / 0 / 375	
Goodness-of-fit on F^2	1.030	
Final R indices	3367 data; I>2σ(I)	R1 = 0.0700, wR2 = 0.1744
	all data	R1 = 0.1599, wR2 = 0.2343
Weighting scheme	w=1/[$\sigma^2(F_o^2)$ + (0.0925P)^2+0.4470P]<br>where P=(F_o^2+2F_c^2)/3	
Largest diff. peak and hole	0.368 and -0.293 eÅ^{-3}	
R.M.S. deviation from mean	0.038 eÅ^{-3}	

1.5 General procedure for the synthesis of pyrazole acid derivatives:

To an oven-dried 25 mL round-bottom flask equipped with a magnetic stirrer, hydrazones (0.6 mmol, 1.2 equiv.), cyclic dienones (0.5 mmol, 1 equiv.) and $CuCl_2 \cdot 2H_2O$ (0.25

mmol, 0.5 equiv.) were weighed and added, followed by 5 mL of acetonitrile. The reaction vessel was stirred in an oil bath at 80 °C in an oxygen atmosphere. The progress of the reaction was monitored by TLC. After 28 h, the reaction mixture was cooled to room temperature, diluted with ethyl acetate, and washed with water. The organic layer was extracted with ethyl acetate and concentrated, and the residue was purified by silica gel column chromatography (100-200 mesh) using petether- ethyl acetate as eluent (hexane/EtOAc, 8:2).

1.6 Characterization data of all the newly synthesized compounds

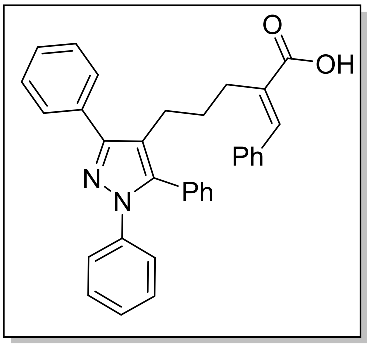

2-benzylidene-5-(1,3,5-triphenyl-1H-pyrazol-4-yl) pentanoic acid (3aa) : Yield: 184.2 mg, 76%; yellow solid; mp: 126-128 °C; IR (ATR): ν_{max} = 2951, 2847, 1670, 1494, 1360, 1264, 1155, 758 cm^{-1}; ^{1}H NMR (400 MHz, DMSO-d_6) δ 12.4 (s, 1H, OH), 7.76-7.74 (m, 2H), 7.50-7.47 (m, 3H), 7.43-7.40 (m, 4H), 7.38-7.32 (m, 5H), 7.29-7.24 (m, 5H), 7.16-7.14 (m, 2H), 2.67-2.63 (m, 2H, CH_2), 2.26-2.22 (m, 2H, CH_2), 1.61-1.52 (m, 2H, CH_2); ^{13}C{^{1}H} NMR (100 MHz, DMSO-d_6) δ 169.3(C=O), , 150.2, 142.0, 140.1, 138.1, 135.6, 134.1, 133.6, 130.7, 130.3, 129.3, 129.3, 129.2, 129.1, 129.0, 128.9, 128.2, 127.8, 127.8, 127.5, 125.0, 118.8, 30.2(CH_2), 27.4(CH_2), 23.9(CH_2); HRMS (ESI-TOF) m/z: [M+H]$^+$ calcd for $C_{33}H_{29}N_2O_2$ 485.2224; found 485.2226.

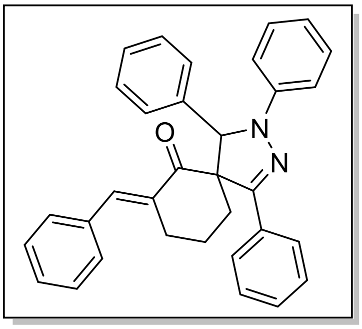

(E)-7-benzylidene-1,3,4-triphenyl-2,3-diazaspiro[4.5]dec-1-en-6-one (3'aa) : Yield: 113.2 mg, 48%; yellow solid; mp: 120-122 °C; IR (ATR): ν_{max} = 2954, 2922, 1670, 1595, 1496, 1255, 1157, 758 cm^{-1}; ^{1}H NMR (400 MHz, DMSO-d_6) δ 7.67 (s, 1H), 7.61-7.55 (m, 4H), 7.51-7.47 (m, 2H), 7.45-7.41 (m, 3H), 7.38-7.30 (m, 6H), 7.14 (d, J = 7.8 Hz, 2H), 6.99 (d, J = 8.0 Hz, 2H), 6.76 (t, J = 7.2 Hz, 1H), 5.53 (s, 1H,HC-N), 2.95-2.81 (m, 2H, CH_2), 2.14-2.06 (m, 1H), 1.81-1.83 (m, 1H), 1.39-1.34 (m, 1H), 0.87-0.81 (m, 1H); ^{13}C{^{1}H} NMR (100 MHz, DMSO-d_6) δ 199.9(C=O), , 150.6, 143.9, 138.1, 135.5, 135.0, 134.8, 130.6, 130.5, 129.3, 128.7, 128.6, 128.5, 126.6, 119.6, 114.5, 74.7, 66.4(Spiro Carbon), 27.5(CH_2), 27.2(CH_2), 19.3(CH_2); **DEPT 135** ^{13}C NMR (100 MHz, DMSO-d_6) δ = 138.6, 131.1, 129.8, 129.2, 129.1, 129.1, 129.0, 129.0, 128.5,

127.7, 120.1, 115.0, 75.2, 28.(CH$_2$), 27.7(CH$_2$), 19.8(CH$_2$); HRMS (ESI-TOF) m/z: [M+H]$^+$ calcd for $C_{33}H_{29}N_2O$ 469.2274; found 469.2274.

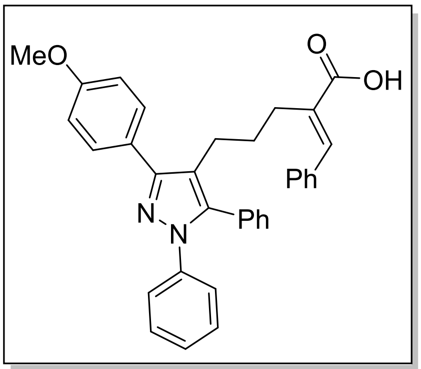

2-benzylidene-5-(3-(4-methoxyphenyl)-1,5-diphenyl-1H-pyrazol-4-yl) pentanoic acid (3ba) : Yield: 212.3 mg, 82%; yellow solid; mp: 168-170 °C; IR (ATR): ν_{max} = 2953, 2834, 1673, 1612, 1494, 1247, 1024, 761 cm^{-1}; ^{1}H NMR (400 MHz, DMSO-d_6) δ 12.4 (s, 1H, OH), 7.67 (d, J = 8.7 Hz, 2H), 7.46 (s, 1H, allylic CH), 7.41-7.39 (m, 3H), 7.36-7.34 (m, 3H), 7.31 (d, J = 7.5 Hz, 2H), 7.26-7.19 (m, 5H), 7.17-7.14 (m, 2H), 7.02 (d, J = 8.7 Hz, 2H), 3.8 (s, 3H, OCH$_3$), 2.63-2.60 (m, 2H, CH$_2$), 2.25-2.21 (m, 2H, CH$_2$), 1.60-1.54 (m, 2H, CH$_2$); ^{13}C{^{1}H} NMR (100 MHz, DMSO-d_6) δ 169.3(C=O), 159.3, 150.1, 141.8, 140.1, 138.1, 135.6, 133.6, 130.7, 130.3, 129.3, 129.2, 129.1, 129.0, 129.0, 128.9, 128.9, 127.3, 126.5, 124.8, 118.4, 114.4, 55.5(OCH$_3$), 30.2(CH$_2$), 27.3(CH$_2$), 23.8(CH$_2$); HRMS (ESI-TOF) m/z: [M+H]$^+$ calcd for $C_{34}H_{31}N_2O_3$ 515.2329; found 515.2330.

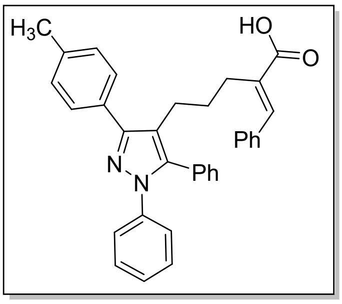

2-benzylidene-5-(1,5-diphenyl-3-(p-tolyl)-1H-pyrazol-4-yl)pentanoic acid (3ca) : Yield: 199.4 mg, 80%; yellow solid; mp: 132-134 °C; IR (ATR): ν_{max} = 3055, 2923, 1669, 1495, 1360, 1265, 967, 762 cm^{-1}; ^{1}H NMR (400 MHz, DMSO-d_6) δ 12.4 (s, 1H, OH), 7.65 (d, J = 7.7 Hz, 2H), 7.47 (s, 1H, allylic CH), 7.42-7.40 (m, 3H), 7.36-7.35 (m, 3H), 7.33-7.31 (m, 2H), 7.29-7.26 (m, 4H), 7.24-7.21 (m, 3H), 7.16-7.14 (m, 2H), 2.65-2.62 (m, 2H, CH$_2$), 2.36 (s, 3H, CH$_3$), 2.26-2.20 (m, 2H, CH$_2$), 1.61-1.53 (m, 2H, CH$_2$); ^{13}C{^{1}H} NMR (100 MHz, DMSO-d_6) δ 169.3(C=O), 150.2, 141.9, 140.1, 138.1, 137.4, 135.6, 133.6, 131.2, 130.7, 130.3, 129.6, 129.3, 129.2, 129.1, 129.0, 128.9, 128.9, 127.7, 127.4, 124.8, 118.6, 30.2(CH$_2$), 27.3(CH$_2$), 23.9(CH$_2$), 21.3(CH$_3$); HRMS (ESI-TOF) m/z: [M+H]$^+$ calcd for $C_{34}H_{31}N_2O_2$ 499.2380; found 499.2384.

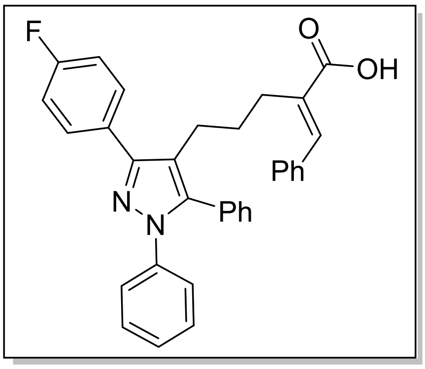

2-benzylidene-5-(3-(4-fluorophenyl)-1,5-diphenyl-1H-pyrazol-4-yl)pentanoic acid (3da) : Yield: 179.4 mg, 71%; yellow solid; mp: 136-138 °C; IR (ATR): ν_{max} = 2922, 1667, 1597, 1496, 1262, 1184, 967, 760 cm^{-1}; ^{1}H NMR (400 MHz, DMSO-d_6) δ 12.4 (s, 1H, OH), 7.80-7.76 (m, 2H), 7.47 (s, 1H, allylic CH), 7.42-7.41 (m, 3H), 7.37-7.32 (m, 6H), 7.30-

7.26 (m, 4H), 7.24-7.21 (m, 2H), 7.17-7.15 (m, 2H), 2.65-2.61 (m, 2H, CH_2), 2.26-2.22 (m, 2H, CH_2), 1.59-1.53 (m, 2H, CH_2); $^{13}C\{^1H\}$ NMR (100 MHz, DMSO-d_6) δ 169.3(C=O), 162.2 (d, $J_{C\text{-}F}$ = 243.1 Hz), 149.4, 142.1, 140.0, 138.2, 135.6, 130.6, 130.5, 130.3, 129.8 (d, $J_{C\text{-}F}$ = 8.0 Hz), 129.3, 129.3, 129.2, 129.1, 128.9, 128.9, 127.5, 124.9, 118.6, 115.9 (d, $J_{C\text{-}F}$ = 21.0 Hz), 30.1(CH_2), 27.3(CH_2), 23.7(CH_2); HRMS (ESI-TOF) m/z: $[M+H]^+$ calcd for $C_{33}H_{28}FN_2O_2$ 503.2129; found 503.2129.

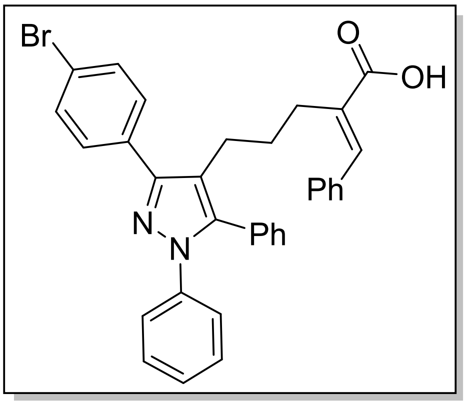

2-benzylidene-5-(3-(4-bromophenyl)-1,5-diphenyl-1H-pyrazol-4-yl)pentanoic acid (3ea) : Yield: 198.1 mg, 66%; yellow solid; mp: 150-152 °C; IR (ATR): ν_{max} = 2951, 2916, 1672, 1593, 1494, 1359, 1247, 757 cm^{-1}; ^1H NMR (400 MHz, DMSO-d_6) δ 12.4 (s, 1H, OH), 7.73-7.65 (m, 4H), 7.47 (s, 1H, allylic CH), 7.42-7.41 (m, 3H), 7.36-7.32 (m, 5H), 7.29-7.20 (m, 5H), 7.17-7.15 (m, 2H), 2.66-2.62 (m, 2H, CH_2), 2.26-2.22 (m, 2H, CH_2), 1.60-1.52 (m, 2H, CH_2); $^{13}C\{^1H\}$ NMR (100 MHz, DMSO-d_6) δ 168.2(C=O), 147.9, 141.2, 138.9, 137.1, 134.5, 132.5, 132.2, 130.9, 129.37, 130.9, 129.3, 129.3, 129.3, 128.6, 128.2, 128.1, 128.0, 127.8, 126.5, 123.8, 120.4, 117.7, 29.0(CH_2), 26.2(CH_2), 22.7(CH_2); HRMS (ESI-TOF) m/z: $[M+H]^+$ calcd for $C_{33}H_{28}BrN_2O_2$ 563.1329; found 563.1335.

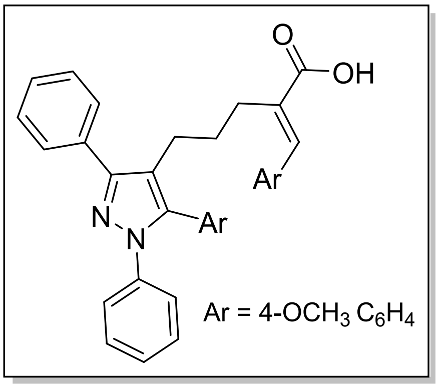

2-(4-methoxybenzylidene)-5-(5-(4-methoxyphenyl)-1,3-diphenyl-1H-pyrazol-4-yl)pentanoic acid (3ab): Yield: 212.6 mg, 78%; white solid; mp: 190-192 °C; IR (ATR): ν_{max} = 2952, 2954, 1671, 1598, 1495, 1247, 1024, 761 cm^{-1}; ^1H NMR (400 MHz, DMSO-d_6) δ 12.3 (s, 1H, OH), 7.75 (d, J = 7.0 Hz, 2H), 7.47 (t, J = 7.4 Hz, 2H), 7.42-7.39 (m, 2H), 7.37-7.33 (m, 2H), 7.28-7.24 (m, 3H), 7.18-7.12 (m, 4H), 6.98 (d, J = 8.7 Hz, 2H), 6.87 (d, J = 8.8 Hz, 2H), 3.79 (s, 3H, OCH_3), 3.77 (s, 3H), 2.68-2.64 (m, 2H, CH_2), 2.28-2.24 (m, 2H, CH_2), 1.60-1.52 (m, 2H, CH_2); $^{13}C\{^1H\}$ NMR (100 MHz, DMSO-d_6) δ 169.6(C=O), 159.8, 159.7, 150.1, 141.3, 140.2, 137.8, 134.2, 131.7, 131.2, 129.3, 129.0, 128.1, 127.9, 127.8, 127.7, 127.4, 124.8, 122.6, 118.7, 114.6, 114.4, 55.6(OCH_3), 55.5(OCH_3), 30.1(CH_2), 27.3(CH_2), 23.9(CH_2); HRMS (ESI-TOF) m/z: $[M+H]^+$ calcd for $C_{35}H_{33}N_2O_4$ 545.2435; found 545.2438.

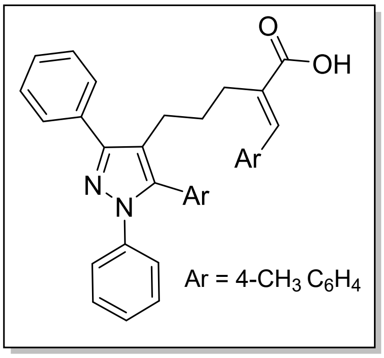

5-(1,3-diphenyl-5-(p-tolyl)-1H-pyrazol-4-yl)-2-(4-methylbenzylidene)pentanoic acid (3ac): Yield: 182.6 mg, 71%; yellow solid; mp: 158-160 °C; IR (ATR): ν_{max} = 2953, 2921, 1670, 1595, 1496, 1265, 1157, 758 cm^{-1}; ^{1}H NMR (400 MHz, DMSO-d_6) δ 12.4 (s, 1H, OH), 7.62 (d, J = 7.7 Hz, 2H), 7.46 (s, 1H, Allylic CH), 7.37-7.34 (m, 2H), 7.29-7.25 (m, 8H), 7.24-7.20 (m, 4H), 7.18-7.14 (m, 2H), 2.63-2.59 (m, 2H, CH_2), 2.51 (s, 3H, CH_3), 2.36 (s, 3H, CH_3), 2.25-2.21 (m, 2H, CH_2), 1.56-1.49 (m, 2H, CH_2); ^{13}C{^{1}H} NMR (100 MHz, DMSO-d_6) δ 169.2(C=O), 163.4, 163.7, 161.26, 161.0, 150.2, 141.0, 139.9, 137.5, 137.0, 133.5, 132.7, 132.6, 132.1, 132.1, 131.6, 131.2, 129.6, 129.3, 127.7, 127.5, 127.1, 127.0, 124.9, 118.8, 116.3, 116.1, 115.9, 115.7, 34.6(CH_2), 29.9(CH_2), 27.1(CH_2), 23.8(CH_3), 21.3(CH_3); HRMS (ESI-TOF) m/z: $[M+H]^+$ calcd for $C_{35}H_{32}N_2NaO_2$ 535.2356; found 535.2347.

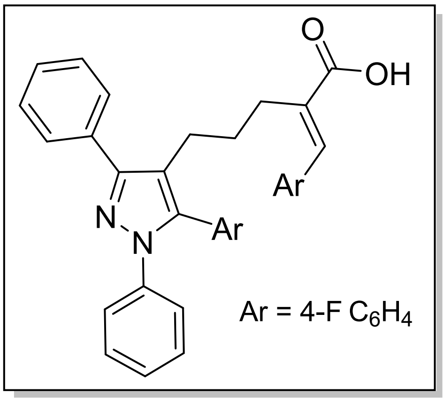

2-(4-fluorobenzylidene)-5-(5-(4-fluorophenyl)-1,3-diphenyl-1H-pyrazol-4-yl)pentanoic acid (3ad): Yield: 178.2 mg, 68%; yellow solid; mp: 173-175 °C; IR (ATR): ν_{max} = 2958, 2917, 1670, 1496, 1279, 1159, 929, 760 cm^{-1}; ^{1}H NMR (400 MHz, DMSO-d_6) δ 12.49 (s, 1H, OH), 7.73 (d, J = 7.5 Hz, 2H), 7.50-7.45 (m, 3H), 7.42-7.34 (m, 3H), 7.30-7.24 (m, 6H), 7.22-7.18 (m, 3H), 7.16-7.14 (m, 2H), 2.64-2.60 (m, 2H, CH_2), 2.25-2.21 (m, 2H, CH_2), 1.56-1.50 (m, 2H, CH_2); ^{13}C{^{1}H} NMR (100 MHz, DMSO-d_6) δ 168.7(C=O), 162.0 (d, $J_{C\text{-}F}$ = 244.7 Hz), 161.7 (d, $J_{C\text{-}F}$ = 245.2 Hz), 149.7, 140.6, 139.4, 136.6, 133.5, 133.0, 132.1 (d, $J_{C\text{-}F}$ = 8.3 Hz), 131.6 (d, $J_{C\text{-}F}$ = 3.2 Hz), 131.0 (d, $J_{C\text{-}F}$ = 8.3 Hz), 128.8, 128.6, 127.8, 127.3, 127.1, 126.5 (d, $J_{C\text{-}F}$ = 3.9 Hz), 124.5, 118.4, 115.7 (d, $J_{C\text{-}F}$ = 21.2 Hz), 115.4 (d, $J_{C\text{-}F}$ = 21.1 Hz), 29.5(CH_2), 26.6(CH_2), 23.3(CH_2); HRMS (ESI-TOF) m/z: $[M+H]^+$ calcd for $C_{33}H_{27}F_2$ N_2O_2 521.2035; found 521.2062.

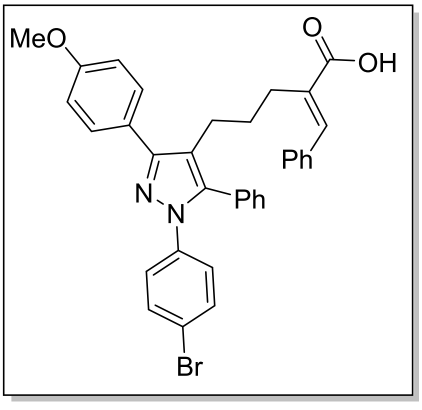

2-benzylidene-5-(1-(4-bromophenyl)-3-(4-methoxyphenyl)-5-phenyl-1H-pyrazol-4-yl)pentanoic acid (3fa) : Yield: 160.1 mg, 54%; yellow solid; mp: 162-164 °C; IR (ATR): ν_{max} = 3057, 2936, 1680, 1612, 1572, 1248, 1293, 830 cm^{-1}; ^{1}H NMR (400 MHz, DMSO-d_6) δ 12.46 (s, 1H, OH), 7.67 (d, J = 8.7 Hz, 2H), 7.52 (d, J = 8.7 Hz, 2H),

7.46-7.42 (m, 4H), 7.35-7.24 (m, 3H), 7.23-7.13 (m, 6H), 7.02 (d, *J* = 8.7 Hz, 2H), 3.80 (s, 3H, CH_3), 2.62-2.58 (m, 2H, CH_2), 2.24-2.20 (m, 2H, CH_2), 1.59-1.51 (m, 2H, CH_2); $^{13}C\{^1H\}$ NMR (100 MHz, DMSO-d_6) δ 168.8(C=O), 158.9, 150.0, 141.4, 138.8, 137.6, 135.1, 133.1, 131.7, 129.9, 129.8, 128.8, 128.8, 128.7, 128.4, 128.4. 126.0, 125.7, 119.5, 118.4, 114.0, 55.0(OCH_3), 29.6(CH_2), 26.8(CH_2), 23.3(CH_2); HRMS (ESI-TOF) m/z: $[M+H]^+$ calcd for $C_{34}H_{30}BrN_2O_3$ 593.1434; found 593.1431.

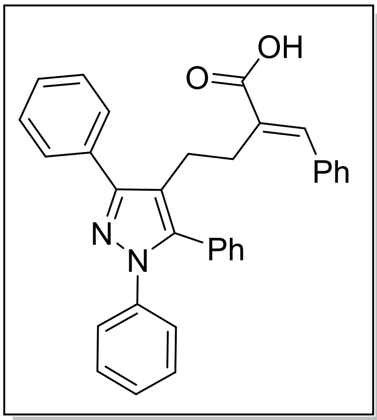

2-benzylidene-4-(1,3,5-triphenyl-1H-pyrazol-4-yl) butanoic acid (5aa): Yield: 169.2 mg, 72%; yellow solid; mp: 168-170 °C; IR (ATR): ν_{max} = 3063, 2948, 1670, 1494, 1358, 1277, 1232, 759 cm^{-1}; ^1H NMR (400 MHz, DMSO-d_6) δ 12.5 (s, 1H, OH), 7.83 (d, *J* = 7.4 Hz, 2H), 7.48-7.38 (m, 7H), 7.34-7.31 (m, 2H), 7.28-7.25 (m, 4H), 7.23-7.18 (m, 4H), 7.03 (d, *J* = 7.6 Hz, 2H), 2.88-2.84 (m, 2H, CH_2), 2.53-2.48 (m, 2H, CH_2); $^{13}C\{^1H\}$ NMR (100 MHz, DMSO-d_6) δ 169.3(C=O), 150.4, 142.1, 140.1, 138.8, 135.4, 133.9, 133.0, 130.5, 130.4, 129.2, 129.2, 129.1, 128.9, 128.9, 128.6, 128.1, 127.5, 126.5, 125.0, 118.6, 118.3, 28.6(CH_2), 23.4(CH_2); HRMS (ESI-TOF) m/z: $[M+H]^+$ calcd for $C_{32}H_{27}N_2O_2$ 471.2067; found 471.2067.

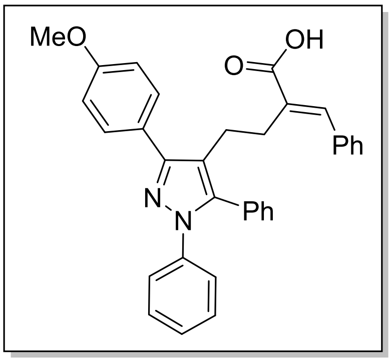

2-benzylidene-4-(3-(4-methoxyphenyl)-1,5-diphenyl-1H-pyrazol-4-yl)butanoic acid (5ba): Yield: 193.0 mg, 77%; yellow solid; mp: 188-190 °C; IR (ATR): ν_{max} = 2951, 2831, 1675, 1493, 1274, 1183, 758, 698 cm^{-1}; ^1H NMR (400 MHz, DMSO-d_6) δ 12.5 (s, 1H, OH), 7.56-7.72 (m, 2H), 7.47 (s, 1H, Allylic CH), 7.44-7.38 (m, 4H), 7.34-7.29 (m, 2H), 7.27-7.24 (m, 3H), 7.21-7.19 (m, 4H), 7.03 (d, *J* = 5.4 Hz, 2H), 6.98 (d, *J* = 8.7 Hz, 2H), 3.82 (s, 3H, CH_3), 2.82-2.79 (m, 2H, CH_2), 2.53-2.48 (m, 2H, CH_2); $^{13}C\{^1H\}$ NMR (100 MHz, DMSO-d_6) δ 169.3(C=O), 159.3, 150.6, 150.2, 141.9, 140.1, 138.8, 135.4, 133.0, 129.45, 129.42, 129.25, 129.21, 129.1, 128.9, 128.6, 126.4, 125.0, 118.3, 118.0, 114.3, 55.5(OCH_3), 28.5(CH_2), 23.4(CH_2); HRMS (ESI-TOF) m/z: $[M+H]^+$ calcd for $C_{33}H_{29}N_2O_3$ 501.2173; found 501.2333.

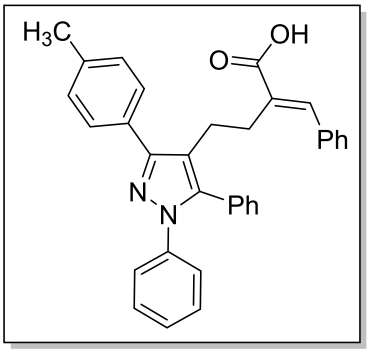

2-benzylidene-4-(1-(4-bromophenyl)-5-phenyl-3-(p-tolyl)-1H-pyrazol-4-yl)butanoic acid (5ca): Yield: 189.2 mg, 78%;

yellow solid; mp: 154-156 °C; IR (ATR): v_{max} = 3060, 2926, 1681, 1628, 1448, 1315, 1211, 824 cm^{-1}; ^{1}H NMR (400 MHz, DMSO-d_6) δ 12.5 (s, 1H, OH), 7.70 (dd, J = 7.8 Hz, 2H), 7.46 (s, 1H, Allylic CH), 7.42-7.35 (m, 3H), 7.33-7.29 (m, 2H), 7.27-7.24 (m, 5H), 7.22-7.16 (m, 5H), 7.01 (d, *J* = 7.6 Hz, 2H), 2.85-2.81 (m, 2H, CH_2), 2.51-2.47 (m, 2H, CH_2), 2.36 (s, 3H,CH_3); $^{13}C\{^1H\}$ NMR (100 MHz, DMSO-d_6) δ 168.8(C=O), 149.9, 141.5, 139.6, 138.3, 136.8, 134.9, 132.6, 130.0, 129.9, 129.0, 128.1, 127.5, 126.9, 124.5, 117.7, 28.1(CH_2), 22.9(CH_2), 20.8(OCH_3); HRMS (ESI-TOF) m/z: $[M+H]^+$ calcd for $C_{33}H_{29}N_2O_2$ 485.2224; found 485.2336.

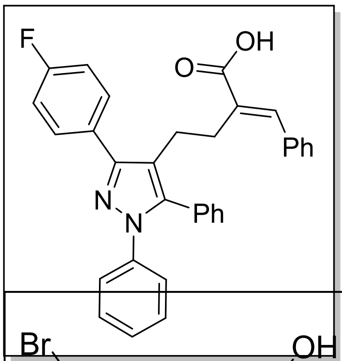

(Z)-2-benzylidene-4-(3-(4-fluorophenyl)-1,5-diphenyl-1H-pyrazol-4-yl)butanoic acid (5da): Yield: 168.2mg, 69%; yellow solid; mp: 174-176 °C; IR (ATR): v_{max} = 3054, 2940, 1678, 1595, 1498, 1444, 1155, 838 cm^{-1}; ^{1}H NMR (400 MHz, DMSO-d_6) δ 12.50 (s, 1H, OH), 7.85-7.81 (m, 2H), 7.47 (s, 1H, Allylic CH), 7.42-7.36 (m, 3H), 7.34-7.26 (m, 4H), 7.25-7.21 (m, 5H), 7.19-7.18 (m, 3H), 7.02 (d, *J* = 7.5 Hz, 2H), 2.85-2.81 (m, 2H, CH_2), 2.48-2.46 (m, 2H, CH_2); $^{13}C\{^1H\}$ NMR (100 MHz, DMSO-d_6) δ 169.3(C=O), 162.2 (d, J_{C-F} = 242.9 Hz), 149.6, 142.1, 140.0, 138.9, 135.4, 132.9, 130.5, 130.4 (d, J_{C-F} = 3.0 Hz), 130.3, 130.2 (d, J_{C-F} = 8.0Hz), 129.5, 129.1, 129.1, 128.9, 128.6, 127.5, 126.5, 125.0, 118.2, 115.7 (d, J_{C-F} = 21.1 Hz), 28.9(CH_2), 23.2(CH_2); HRMS (ESI-TOF) m/z: $[M+H]^+$ calcd for $C_{32}H_{26}FN_2O_2$ 489.1973; found 489.1974.

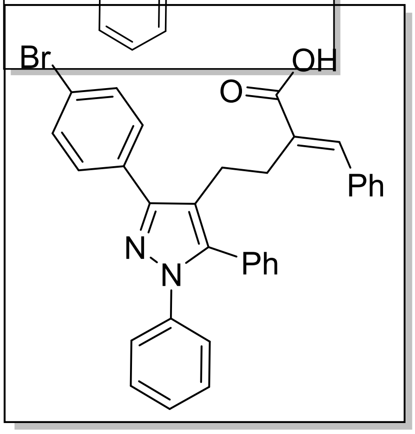

2-benzylidene-4-(3-(4-bromophenyl)-1,5-diphenyl-1H-pyrazol-4-yl)butanoic acid (5ea): Yield: 184.6 mg, 67%; yellow solid; mp: 204-206 °C; IR (ATR): v_{max} = 3053, 2952, 1672, 1594, 1495, 1447, 1157, 830 cm^{-1}; ^{1}H NMR (400 MHz, DMSO-d_6) δ 12.55 (s, 1H, OH), 7.76 (d, *J* = 8.4 Hz, 2H), 7.59 (d, *J* = 8.2 Hz, 2H), 7.46 (s, 1H, Allylic CH), 7.43-7.36 (m, 4H), 7.34-7.30 (m, 2H), 7.28-7.24 (m, 3H), 7.22-7.17 (m, 4H), 7.01 (d, *J* = 7.6 Hz, 2H), 2.85-2.81 (m, 2H, CH_2), 2.49-2.46 (m, 2H, CH_2); $^{13}C\{^1H\}$ NMR (100 MHz, DMSO-d_6) δ 168.8(C=O), 148.7, 141.9, 139.5, 139.5, 138.5, 134.9, 132.6, 132.3, 131.3, 130.0, 129.7, 129.6, 128.7, 128.7, 128.6, 128.6, 128.4, 128.2, 127.1, 124.6, 121.0, 117.8, 28.0(CH_2), 22.8(CH_2); HRMS (ESI-TOF) m/z: $[M+H]^+$ calcd for $C_{32}H_{26}BrN_2O_2$ 549.1172; found 549.1174.

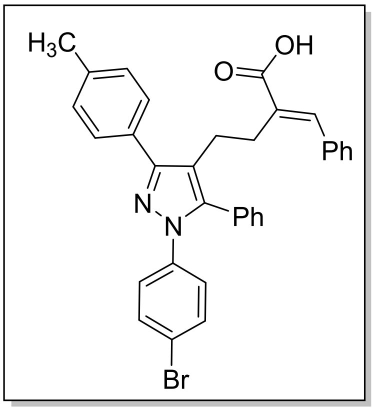

2-benzylidene-4-(1-(4-bromophenyl)-5-phenyl-3-(p-tolyl)-1H-pyrazol-4-yl)butanoic acid (5fa): Yield: 165.1 mg, 58%; yellow solid; mp: 188-190 °C; IR (ATR): ν_{max} = 3056, 2979, 1680, 1590, 1493, 1412, 1265, 824 cm^{-1}; ^{1}H NMR (400 MHz, DMSO-*d*$_6$) δ 12.50 (s, 1H, OH), 7.70-7.67 (m, 2H), 7.52-7.49 (m, 2H), 7.46-7.38 (m, 4H), 7.30-7.20 (m, 5H), 7.19-7.11 (m, 4H), 7.02-6.99 (m, 2H), 2.84-2.79 (m, 2H, CH_2), 2.48-2.44 (m, 2H, CH_2), 2.36 (s, 3H, CH_3); ^{13}C{^{1}H} NMR (100 MHz, DMSO-*d*$_6$) δ 169.3(C=O), 150.8, 142.0, 139.3, 138.8, 137.4, 135.4, 133.0, 132.1, 130.8, 130.5, 130.1, 129.5, 129.2, 129.1, 128.9, 129.6, 128.0, 126.7, 120.1, 118.6, 28.5(CH_2), 23.4(CH_2), 21.3(CH_3); HRMS (ESI-TOF) m/z: [M+H]$^+$ calcd for $C_{33}H_{28}BrN_2O_2$ 563.1329; found 563.1461.

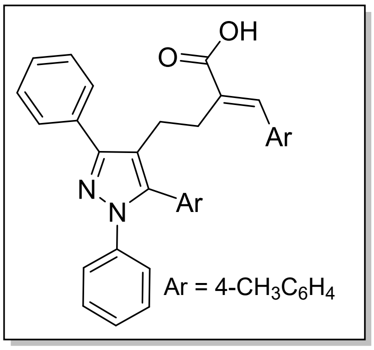

4-(1,3-diphenyl-5-(p-tolyl)-1H-pyrazol-4-yl)-2-(4-methylbenzylidene)butanoic acid (5ac): Yield: 198.0 mg, 84%; yellow solid; mp: 212-214 °C; IR (ATR): ν_{max} = 2956, 2925, 1670, 1495, 1449, 1270, 831, 760 cm^{-1}; ^{1}H NMR (400 MHz, DMSO-d$_6$) δ 12.4 (s, 1H, OH), 7.84 (d, *J* = 6.7Hz, 2H), 7.45-7.36 (m, 4H), 7.35-7.31 (m, 2H), 7.28-7.25 (m, 1H,), 7.22-7.19 (m, 4H), 7.16-7.14 (m, 2H), 6.98-6.91 (m, 4H), 2.84-2.80 (m, 2H, CH_2), 253-2.51 (m, 2H, CH_2), 2.35 (s, 3H, CH_3), 2.29 (s, 3H, CH_3); ^{13}C{^{1}H} NMR (100 MHz, DMSO-d$_6$) δ 168.4(C=O), 149.2, 141.1, 139.1, 137.7, 137.2, 137.2, 132.9, 131.4, 131.0, 129.3(2C), 128.7(2C), 128.4(2C), 128.19(2C), 128.16(2C), 127.7(2C), 127.1(2C), 127.0, 126.4, 126.3, 124.0(2C), 117.1, 27.6(CH_2), 22.3(CH_2), 20.3(CH_3), 20.2(CH_3); HRMS (ESI-TOF) m/z: [M+H]$^+$ calcd for $C_{34}H_{31}N_2O_2$ 499.2380; found 499.2384.

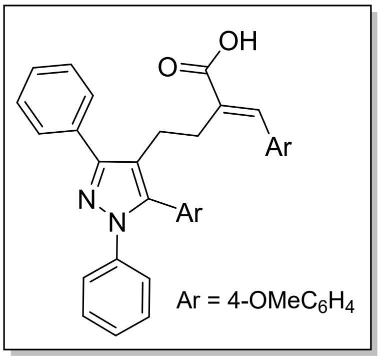

2-(4-methoxybenzylidene)-4-(5-(4-methoxyphenyl)-1,3-diphenyl-1H-pyrazol-4-yl)butanoic acid (5ab): Yield: 212.0 mg, 80%; yellow solid; mp: 218-220 °C; FT-IR (ATR): ν_{max} = 3018, 2947, 1674, 1495, 1419, 1240, 1156, 756 cm^{-1}; ^{1}H NMR (400 MHz, DMSO-*d*$_6$) δ 12.41 (s, 1H, OH), 7.86 (d, *J* = 6.7 Hz, 2H), 7.47-7.38 (m, 4H), 7.35-7.32 (m, 2H), 7.28-7.22 (m, 5H), 7.00-6.95 (m, 4H), 6.69 (d, *J* = 8.9 Hz, 2H), 3.78 (s, 3H, CH_3), 3.76 (s, 3H, CH_3), 2.85-2.81 (m, 2H, CH_2), 2.54-2.52 (m, 2H, CH_2); ^{13}C{^{1}H} NMR (100 MHz,

DMSO-d_6) δ 169.2(C=O), 159.2, 159.2, 149.8, 141.6, 139.7, 138.0, 133.5, 131.5, 130.6, 130.1, 128.7, 128.4, 127.7, 127.6, 127.2, 126.9, 124.5, 122.0, 117.7, 114.1, 113.9, 55.1(OCH_3), 55.0(OCH_3), 28.3(CH_2), 22.9(CH_2); HRMS (ESI-TOF) m/z: $[M+H]^+$ calcd for $C_{34}H_{31}N_2O_4$ 531.2278; found 531.2292.

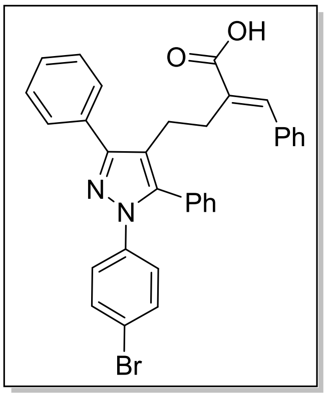

2-benzylidene-4-(1-(4-bromophenyl)-3,5-diphenyl-1H-pyrazol-4-yl)butanoic acid (5fa): Yield: 154.1 mg, 56%; yellow solid; mp: 176-178 °C; IR (ATR): ν_{max} = 3064, 1682, 1598, 1554, 1505, 1453, 1156, 839 cm^{-1}; ^{1}H NMR (400 MHz, DMSO-d_6) δ 12.60 (s, 1H, OH), 7.78 (d, J = 6.9 Hz, 2H), 7.44-7.40 (m, 4H), 7.36-7.31 (m, 3H), 7.30-7.24 (m, 4H), 7.22-7.18 (m, 3H), 7.10- 7.06 (m, 2H), 7.01-6.97 (m, 2H), 2.82-2.78 (m, 2H, CH_2), 2.47-2.44 (m, 2H, CH_2); ^{13}C{^{1}H} NMR (100 MHz, DMSO-d_6) δ 169.1(C=O), 163.7, 161.2, 150.3, 141.2, 139.9, 137.8, 133.8, 132.9, 132.8, 131.3, 131.2, 129.3, 128.9, 128.1, 125.1, 118.4, 116.2, 116.0, 115.9, 115.6, 28.3(CH_2), 23.2(CH_2); HRMS (ESI-TOF) m/z: $[M+H]^+$ calcd for $C_{32}H_{26}BrN_2O_2$ 549.1172; found 549.1174.

1.7. Procedure for control experiments (Scheme 5 in the manuscript)

Reaction 1.

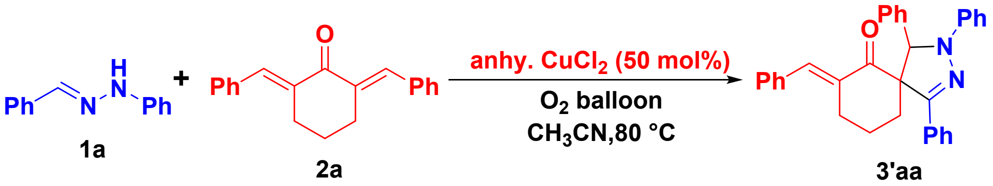

To an oven-dried 25 mL round bottom flask equipped with a magnetic stirrer, 1-benzylidene-2-phenylhydrazine, **1a** (0.6 mmol, 1.2 equiv.), 2,6-di((E)-benzylidene) cyclohexan-1-one, **2a** (0.5 mmol) and $CuCl_2$ anhydrous (0.25 mmol, 0.5 equiv.) were weighed and added followed by 5 mL of acetonitrile solvent. The reaction vessel was stirred in an oil bath at 80 °C under an oxygen atmosphere. The progress of the reaction was monitored by TLC. After 28h, the reaction predominantly yielded the spiro pyrazoline derivative, **3'aa** as

shown in (scheme 5). The reaction mixture was cooled to room temperature, diluted with ethyl acetate, and washed with water. The organic layer was concentrated, and the residue was purified by silica gel column chromatography using pet ether-ethyl acetate (hexane/EtOAc, 9:1) as eluent.

Reaction 2.

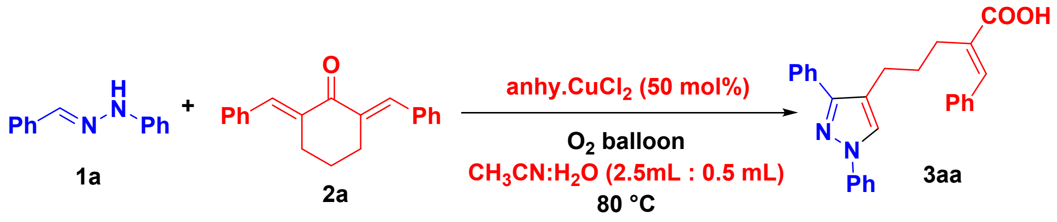

To an oven-dried 25 mL round bottom flask equipped with a magnetic stirrer, 1-benzylidene-2-phenylhydrazine, **1a** (0.30 mmol, 1.2 equiv.), 2,6-di((E)-benzylidene) cyclohexan-1-one, **2a** (0.25 mmol, 1.0 equiv.) and $CuCl_2$ Anhydrous (0.125 mmol, 0.5 equiv.) were weighed and added followed by 2.5 mL of acetonitrile and 0.5 mL H_2O as solvent. The reaction vessel was stirred in an oil bath at 80 °C under an oxygen atmosphere. The progress of the reaction was monitored by TLC and the reaction exclusively offered only the 2-benzylidene-5-(1,3,5-triphenyl-1H-pyrazol-4-yl) pentanoic acid derivative, **3aa**. The reaction mixture was cooled to room temperature, diluted with ethyl acetate, and washed with water. The organic layer was concentrated, and the residue was purified by silica gel column chromatography using pet ether-ethyl acetate (hexane/EtOAc, 8:2) as eluent.

Reaction 3.

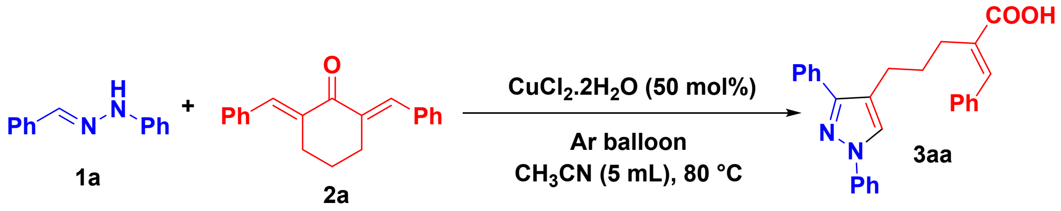

To an oven-dried 25 mL round bottom flask equipped with a magnetic stirrer, 1-benzylidene-2-phenylhydrazine, **1a** (0.6 mmol, 1.2 equiv.), 2,6-di((E)-benzylidene) cyclohexan-1-one, **2a** (0.5 mmol) and $CuCl_2 \cdot 2H_2O$ (0.25 mmol, 0.5 equiv.) were weighed and added followed by 5 mL of acetonitrile solvent. The reaction vessel was stirred in an oil bath at 80 °C under an Argon atmosphere. The progress of the reaction was monitored by TLC, and the reaction exclusively offered only the 2-benzylidene-5-(1,3,5-triphenyl-1H-pyrazol-4-yl) pentanoic acid

derivative, **3aa**. The reaction mixture was cooled to room temperature, diluted with ethyl acetate, and washed with water. The organic layer was concentrated, and the residue was purified by silica gel column chromatography using pet ether-ethyl acetate (hexane/EtOAc, 8:2) as eluent, and the reaction yield was low (25%) under argon.

1.8 Procedure for Gram-scale synthesis

2-benzylidene-5-(1,3,5-triphenyl-1H-pyrazol-4-yl) pentanoic acid (3aa):

To an oven-dried 50 mL round bottom flask equipped with a magnetic stirrer, 1-benzylidene-2-phenylhydrazine, **1a** (863 mg, 4.44 mmol, 1.2 equiv.), 2,6-di((E)-benzylidene) cyclohexan-1-one, **2a** (670 mg, 3.7 mmol, 1 equiv.) and $CuCl_2 \cdot 2H_2O$ (316 mg, 1.85 mmol, 0.5 equiv.) were weighed and added followed by 20 mL of acetonitrile solvent. The reaction vessel was stirred in an oil bath at 80 °C under an oxygen atmosphere. The progress of the reaction was monitored by TLC and after 28h, the reaction exclusively offered only the 2-benzylidene-5-(1,3,5-triphenyl-1H-pyrazol-4-yl) pentanoic acid, **3aa** (Scheme 4 in the manuscript). The reaction mixture was cooled to room temperature, diluted with ethyl acetate, and washed with water. The organic layer was concentrated, and the residue was purified by silica gel column chromatography using pet ether-ethyl acetate (hexane/EtOAc, 8:2) as eluent to afford **3aa** in 56% yield, 1.001 gm.

2-benzylidene-4-(1,3,5-triphenyl-1H-pyrazol-4-yl) butanoic acid (5aa):

To an oven-dried 50 mL round bottom flask equipped with a magnetic stirrer, 1-benzylidene-2-phenylhydrazine, **1a** (1000 mg, 5.1 mmol, 1.2 equiv.), 2,5-di((E)-benzylidene) cyclopentan-1-one, **4a** (1093.42 mg, 4.2 mmol, 1 equiv.) and $CuCl_2 \cdot 2H_2O$ (375 mg, 2.1 mmol, 0.5 equiv.) were weighed and added followed by 20 mL of acetonitrile solvent. The reaction vessel was stirred in an oil bath at 80 °C under an oxygen atmosphere. The progress of the reaction was monitored by TLC and after 28h, the reaction exclusively offered only the 2-benzylidene-4-(1,3,5-triphenyl-1H-pyrazol-4-yl) butanoic acid, **5aa** (Scheme 4). The reaction mixture was cooled to room temperature, diluted with ethyl acetate, and washed with water. The organic layer was concentrated, and the residue was purified by silica gel column chromatography using pet ether-ethyl acetate (hexane/EtOAc, 8:2) as eluent. (1.205 gm, Yield= 61%)

1.9 References:

S1. E. Jedlovská, L. Fišera, A. Lévai, G. Tóth and B. Balázs, *J. Heterocyclic Chem.*, 1999, **36,** 1087-1090.
S2. A. L. Gerten, M. C. Slade, K. M. Pugh and L. M. Stanley, *Org. Biomol. Chem.,* 2013, **11**, 7834.
S3. H. Gazzeh, S. Boudriga, M. Askri, A. Khatyr, M. Knorr, C. Strohmann, C. Golz, Y. Rousselin and M. M. Kubicki, *RSC Adv*., 2016, **6**, 49868-49875.
S4. A. S. Girgis, Y. A. Ibrahim, N. Mishriky, J. N. Lisgarten, B. S. Potter and R. A. Palmer, *Tetrahedron*., 2001, **57**, 2015-2019.

2.0 ^{1}H and ^{13}C {^{1}H} NMR spectra of new compounds

^{1}H NMR of cyclopentanone dienone compound in $CDCl_3$

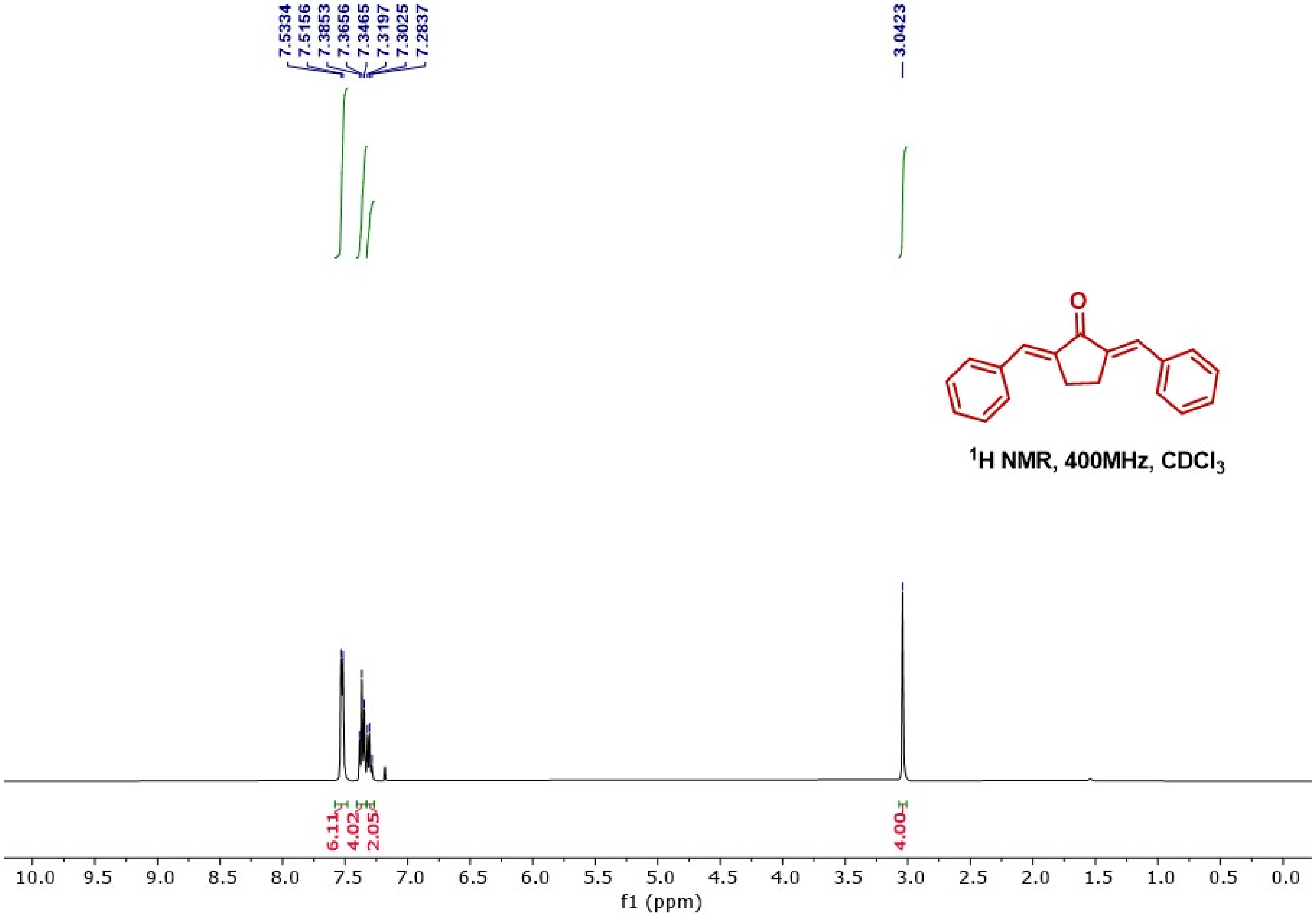

^{1}H NMR of cyclopentanone dienone compound in $CDCl_3$

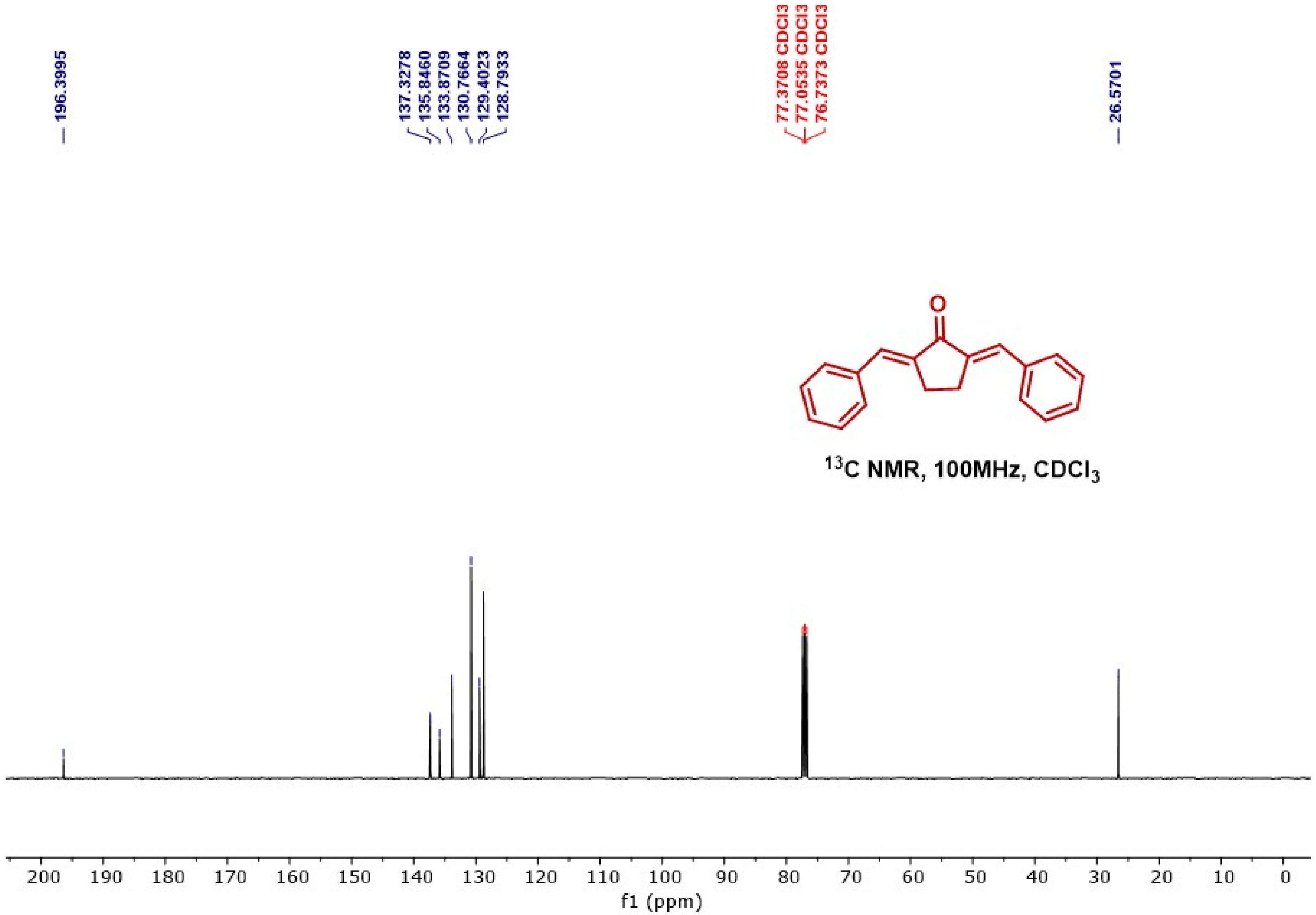

^{1}H NMR spectrum of compound 3aa in DMSO-d_6

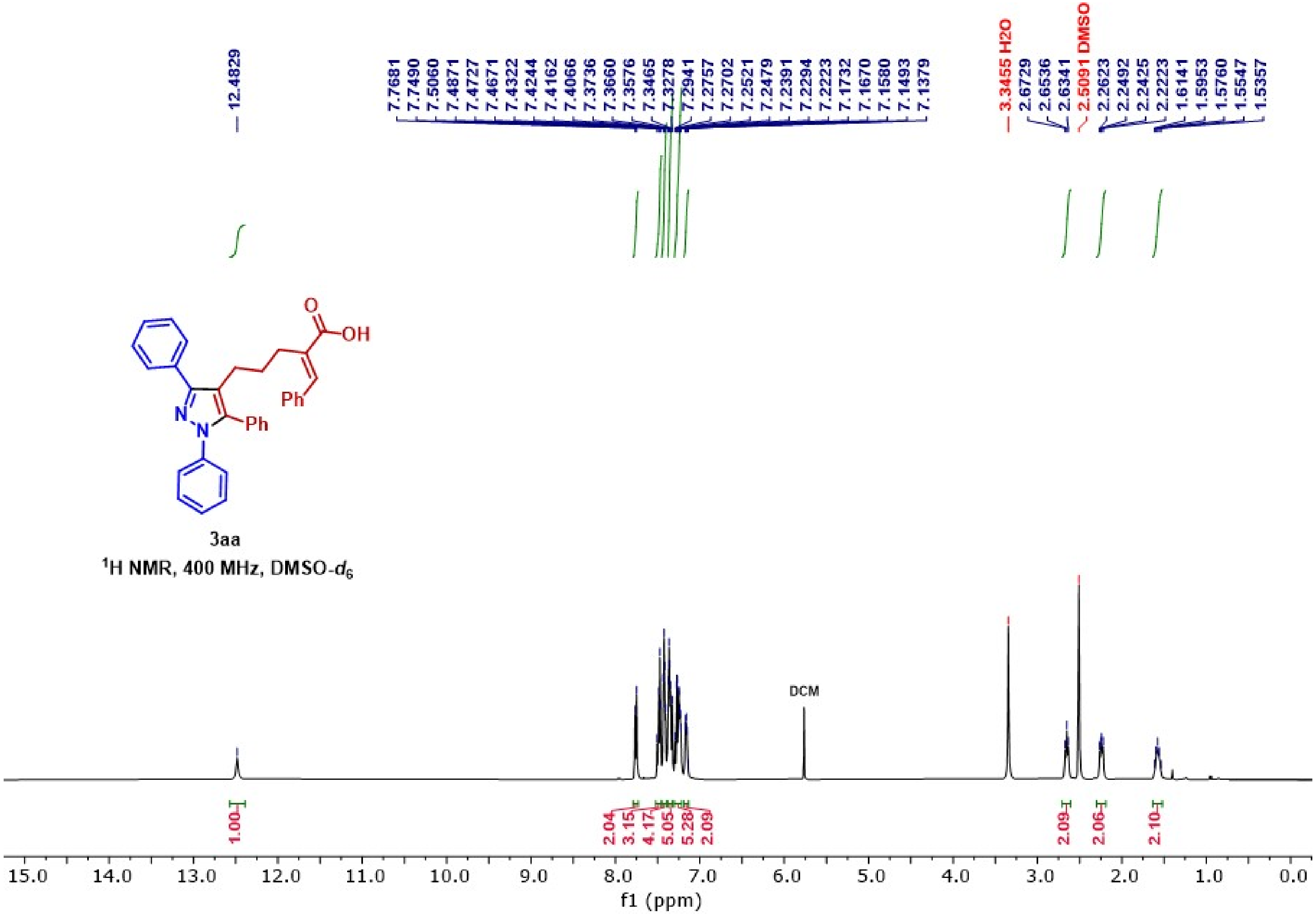

D_2O exchange ^1H NMR spectrum of compound 3aa in DMSO-d_6

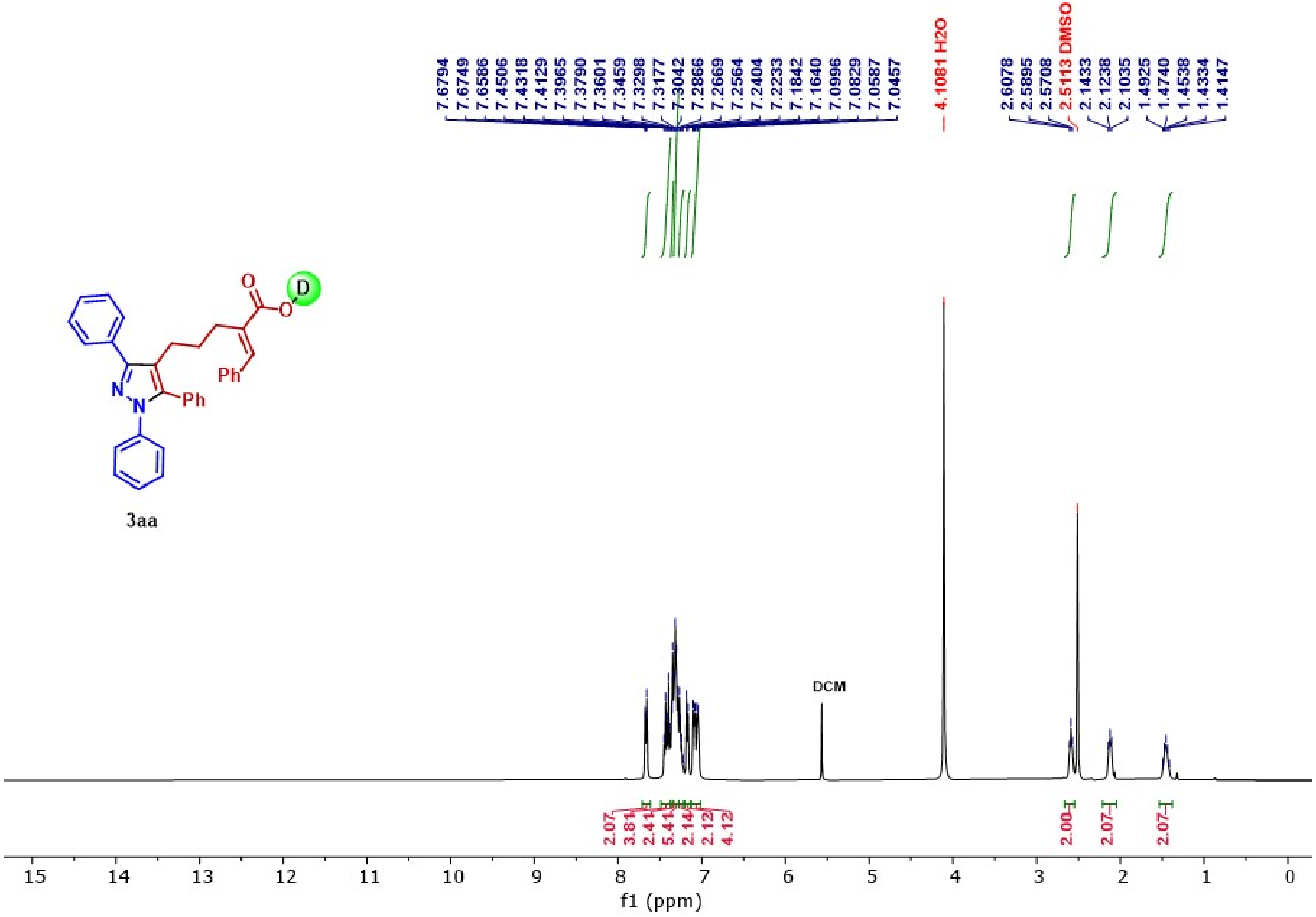

$^{13}C\{^1H\}$ NMR spectrum of compound 3aa in DMSO- d_6

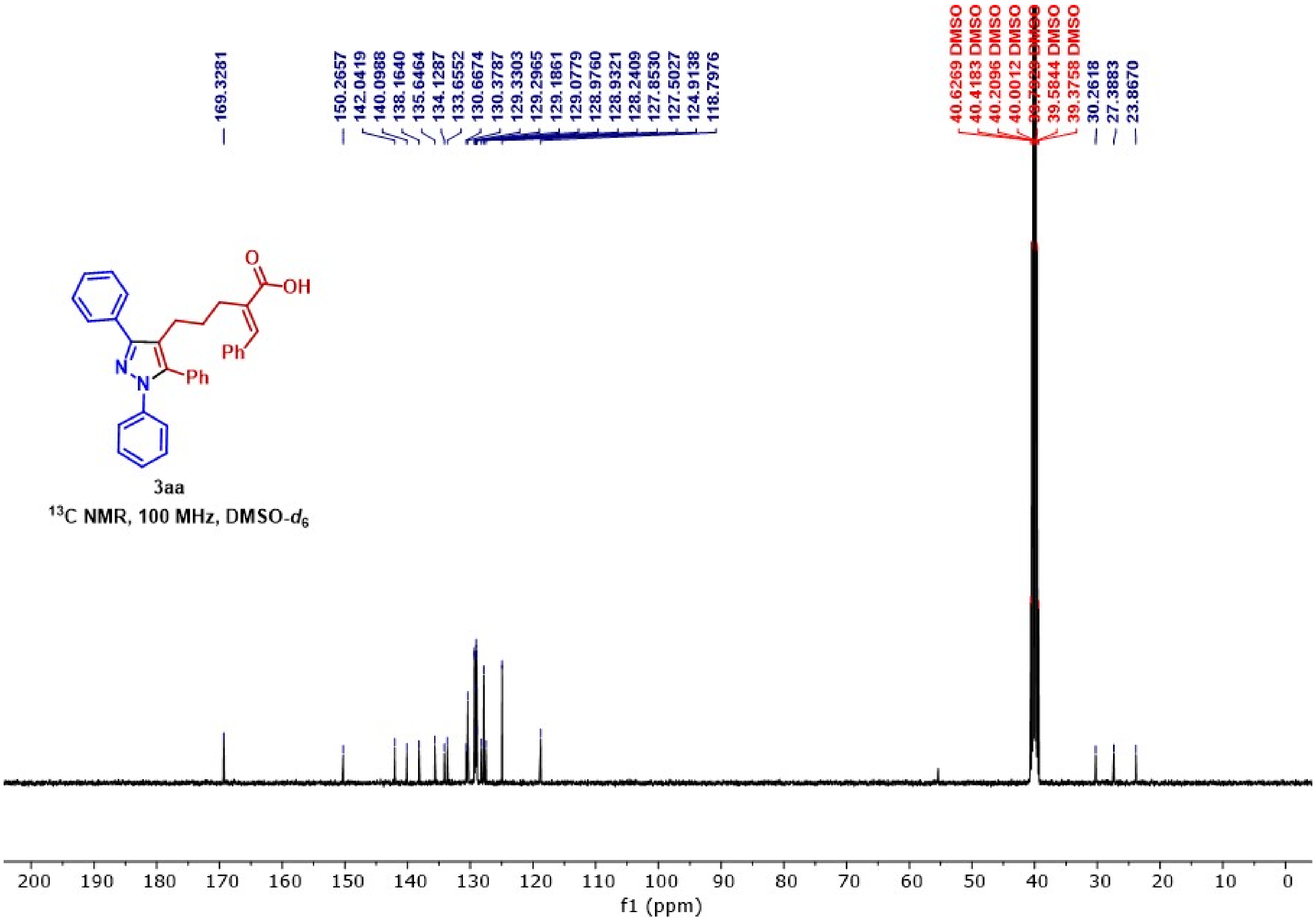

^{1}H NMR spectrum of compound 3’aa in DMSO-d_6

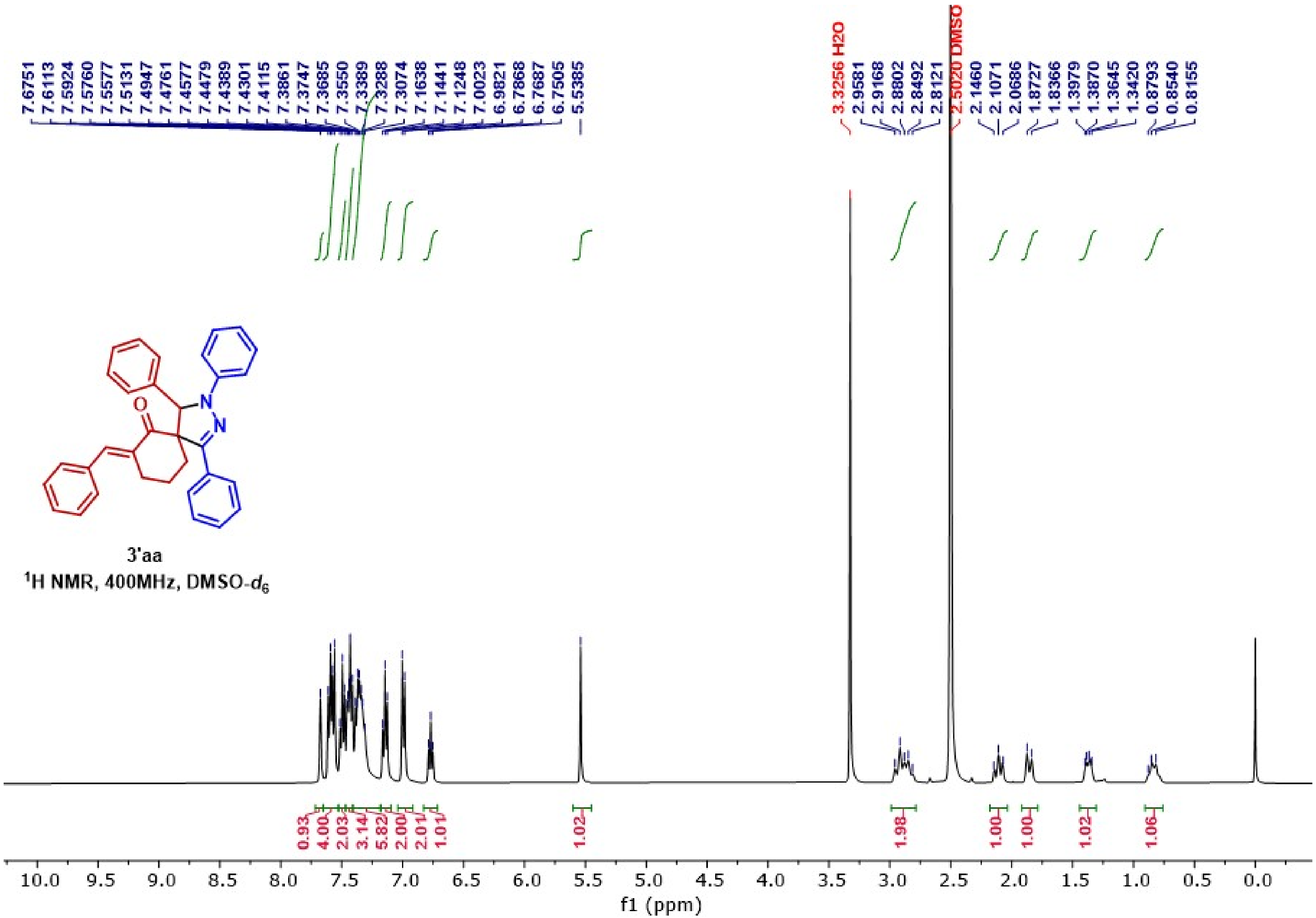

$^{13}C\{^1H\}$ NMR spectrum of compound 3'aa in DMSO-d$_6$

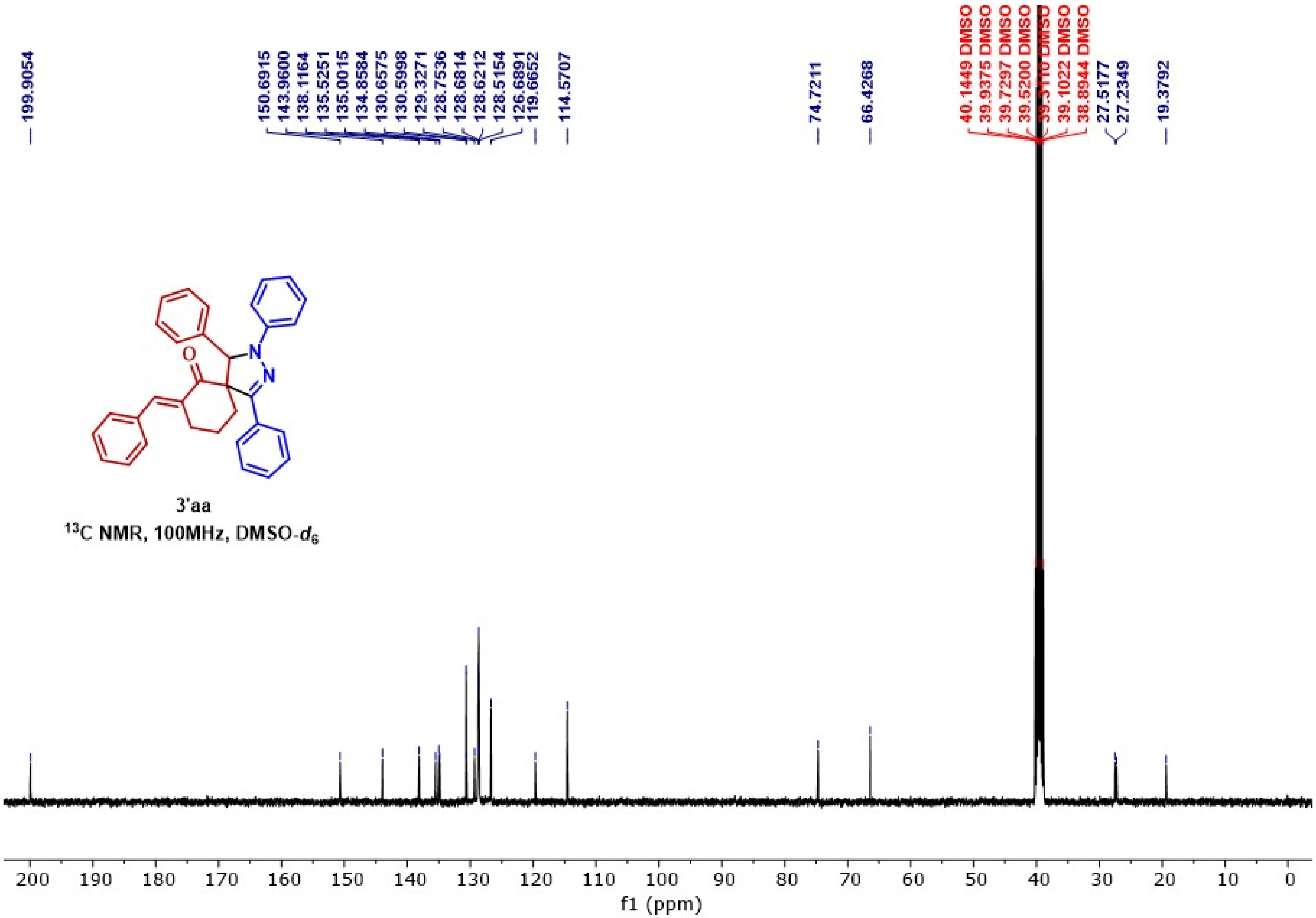

DEPT 135 ^{13}C NMR spectrum of compound 3'aa in DMSO-d_6

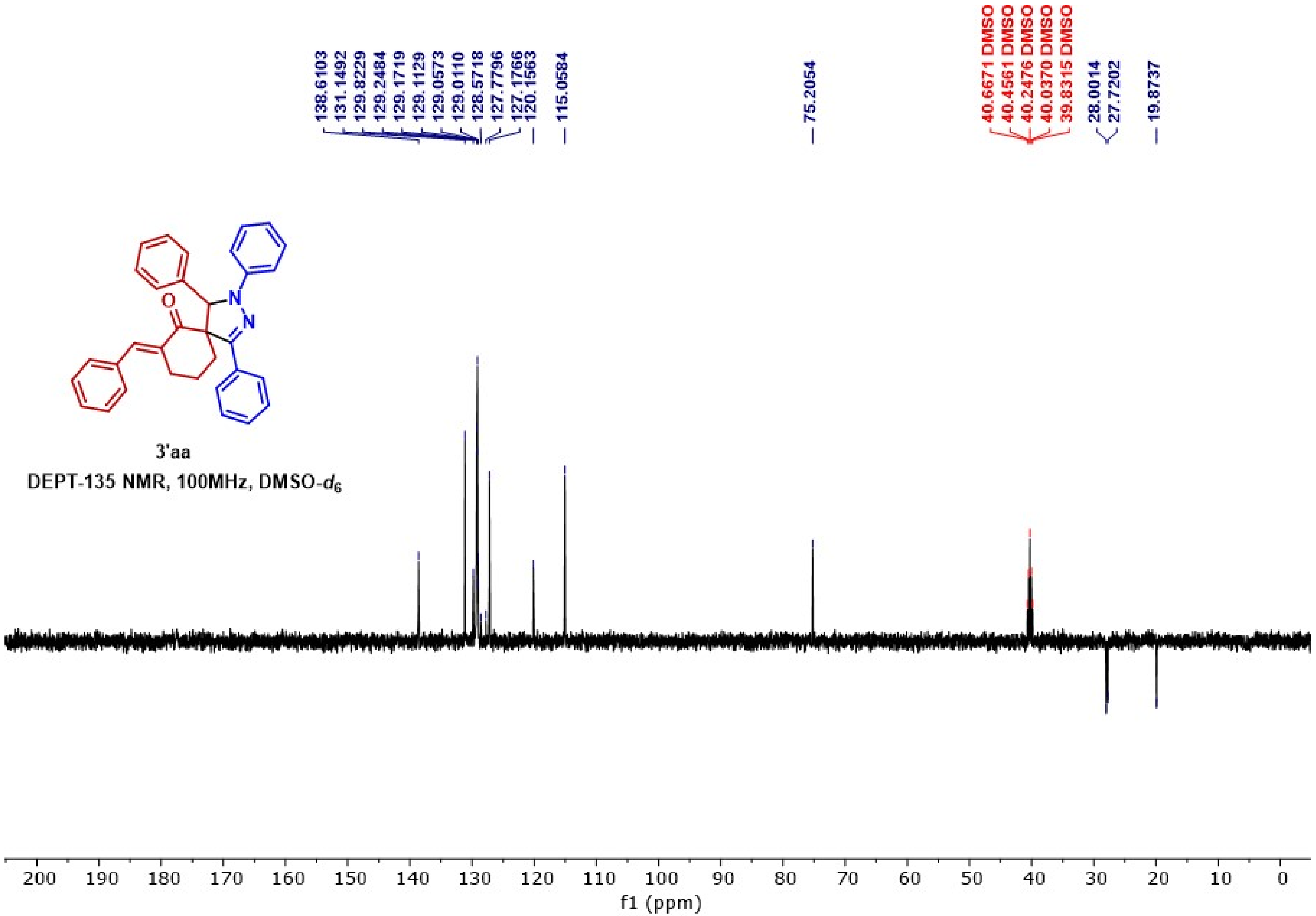

^1H NMR spectrum of compound 3ba in DMSO-d_6

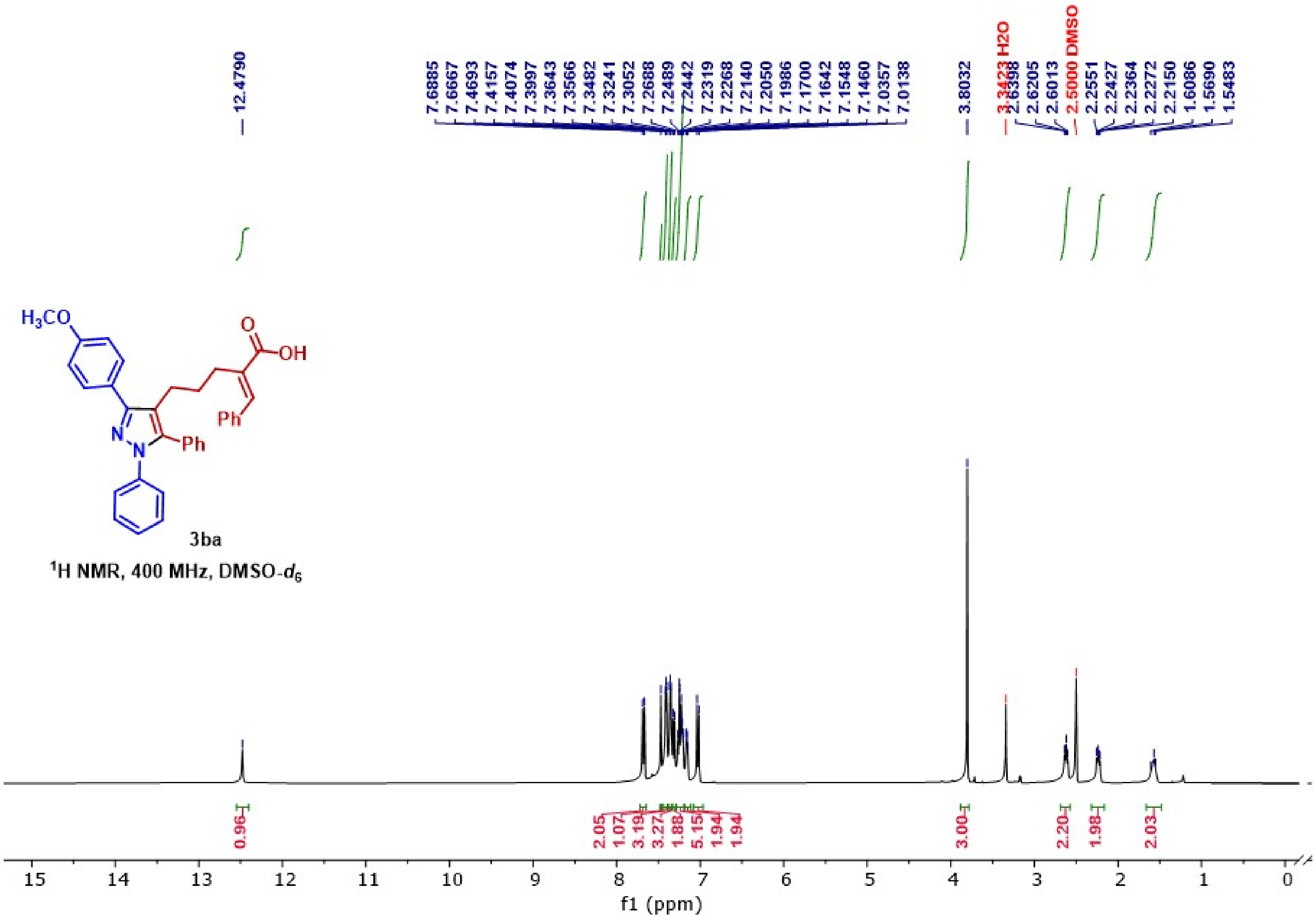

$^{13}C\{^{1}H\}$ NMR spectrum of compound 3ba in DMSO-d_6

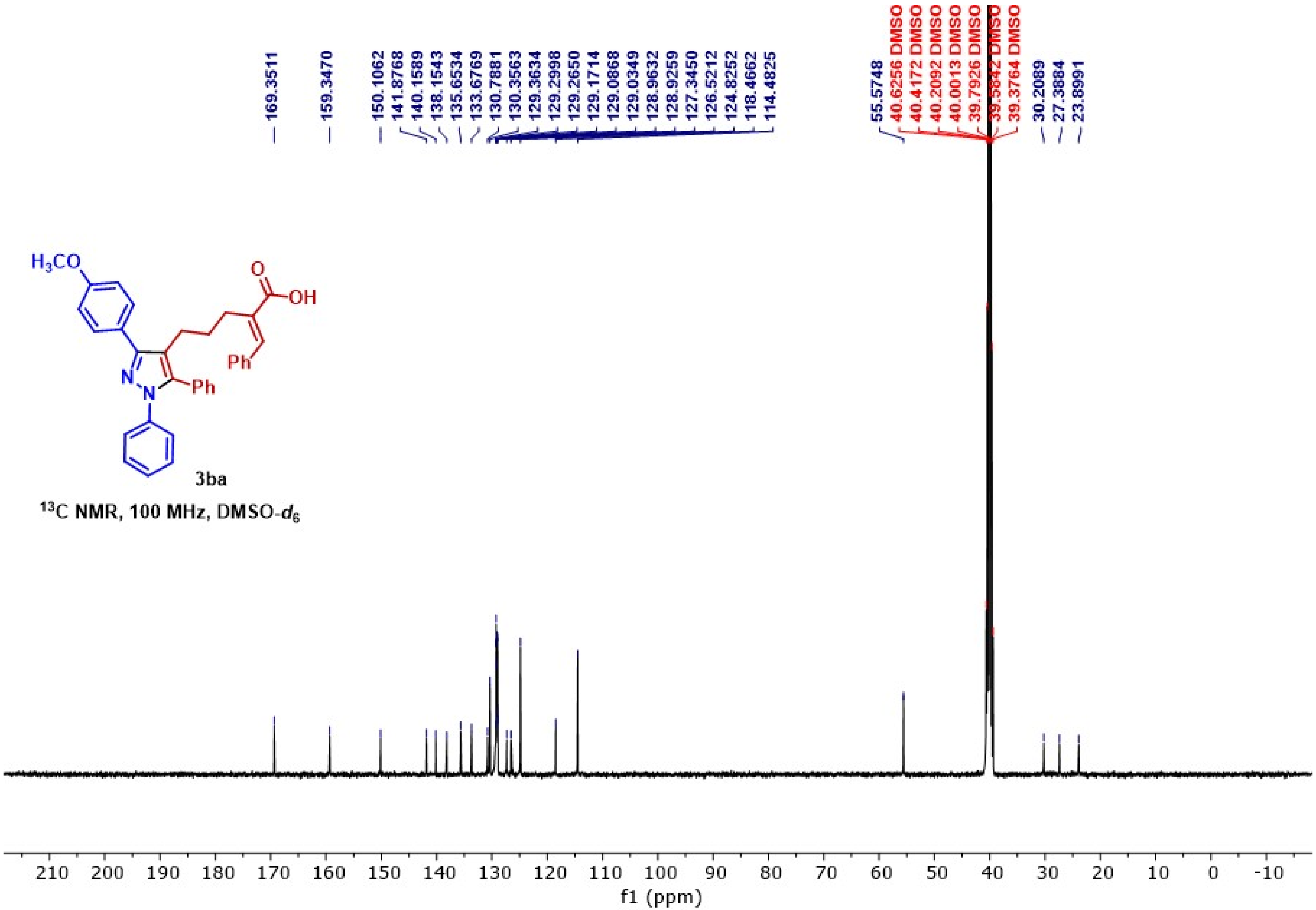

^{1}H NMR spectrum of compound 3ca in DMSO-d_6

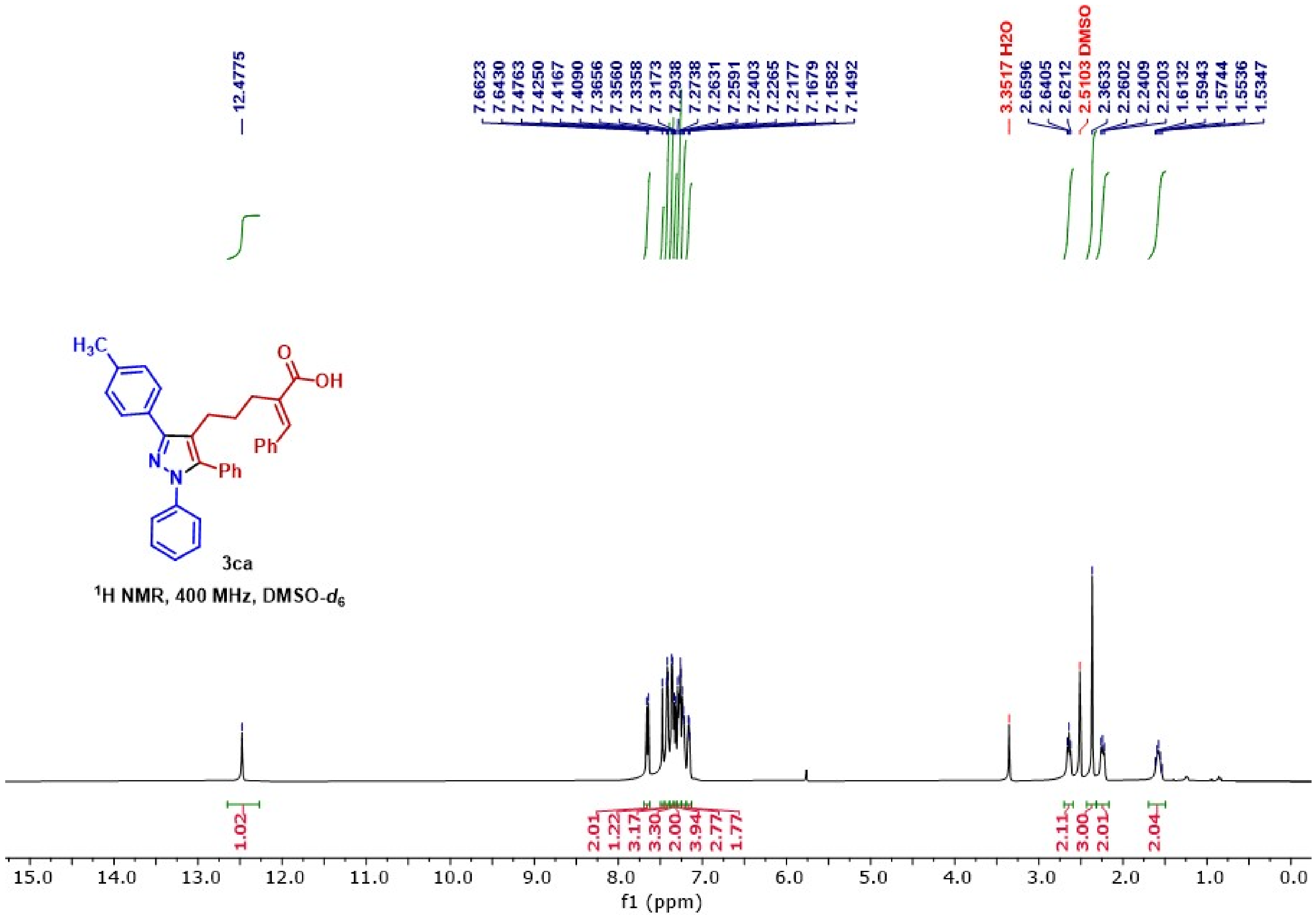

$^{13}C\{^1H\}$ NMR spectrum of compound 3ca in DMSO-d_6

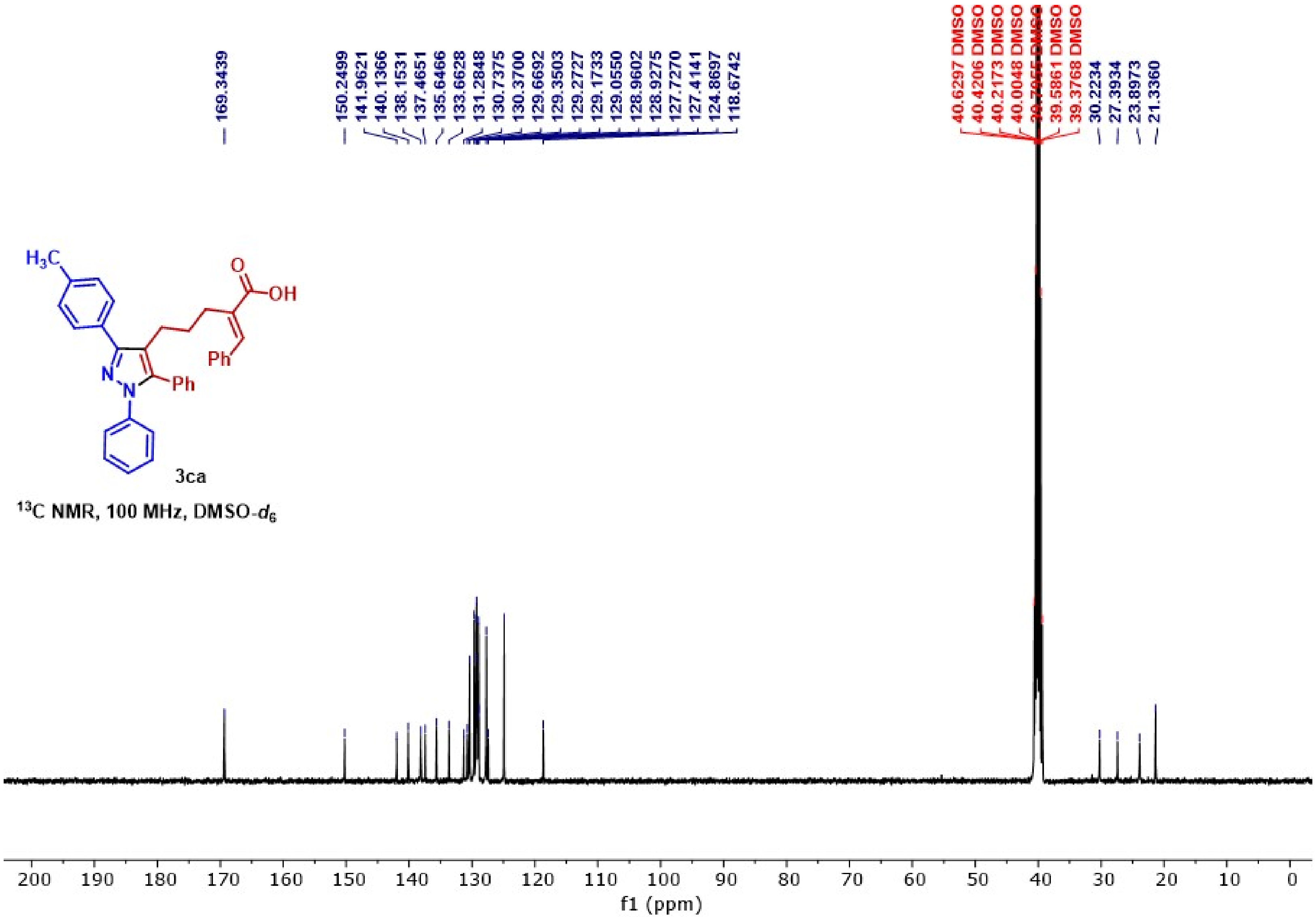

^{1}H NMR spectrum of compound 3da in DMSO-d_6

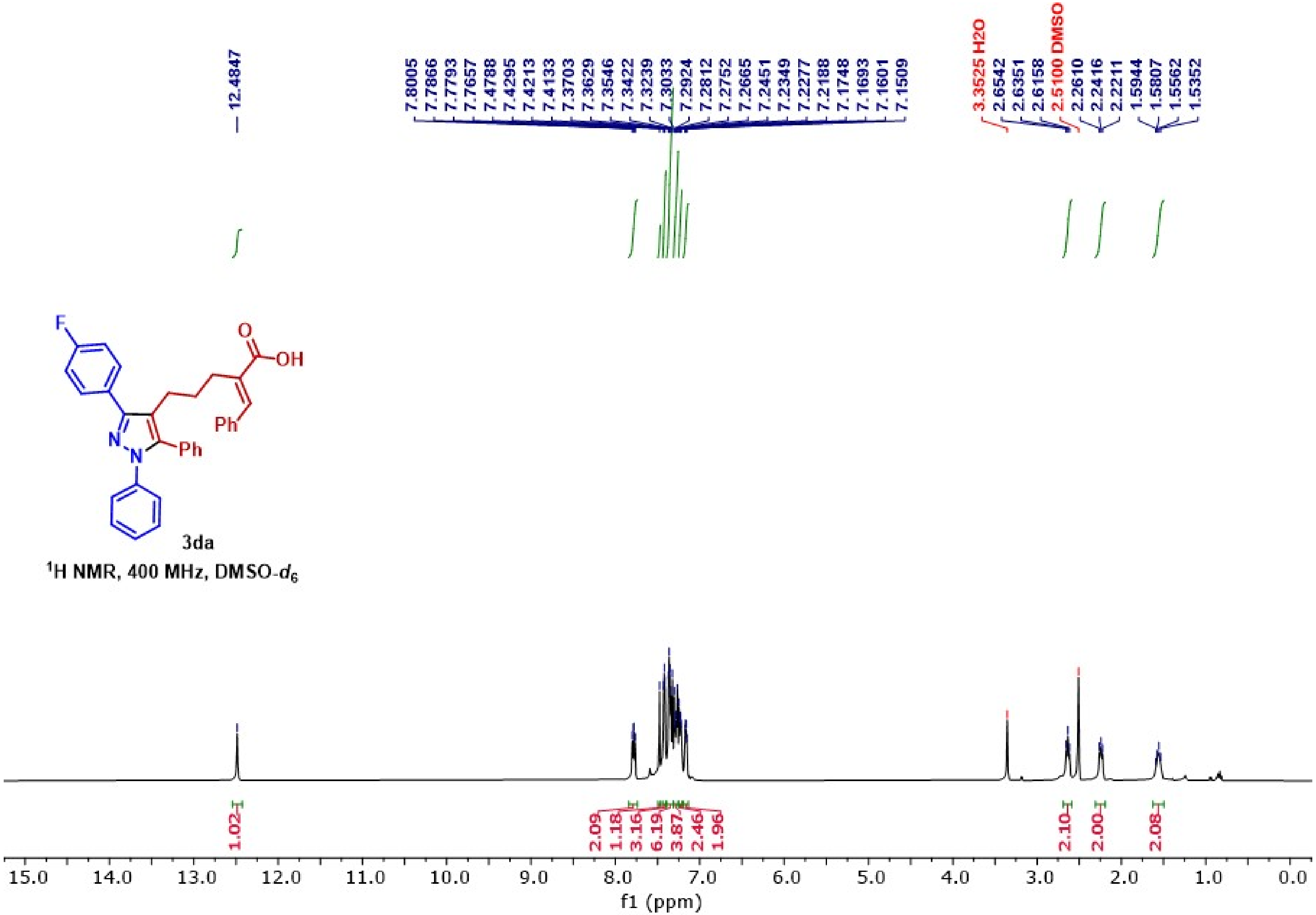

$^{13}C\{^{1}H\}$ NMR spectrum of compound 3da in DMSO-d$_6$

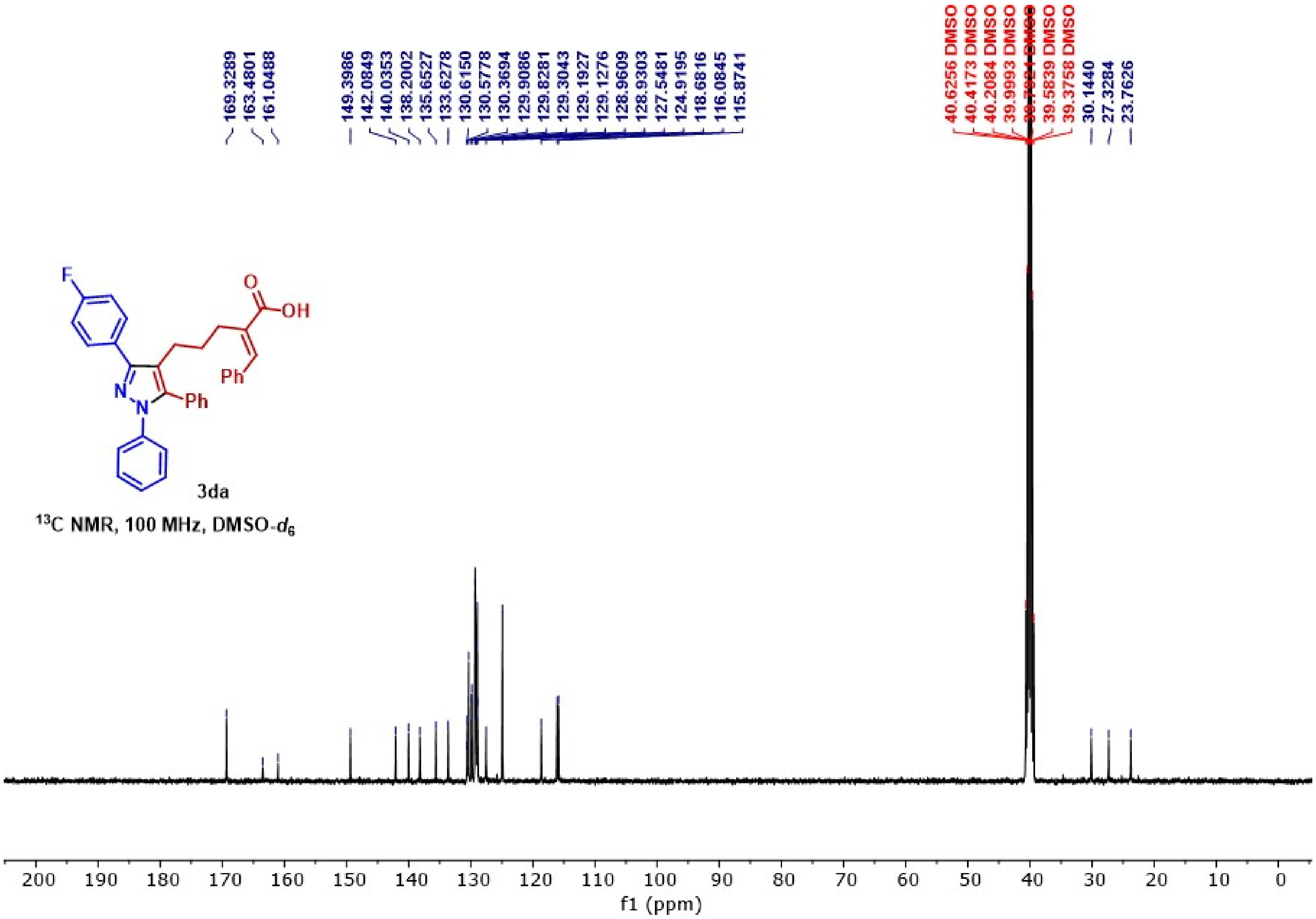

^{1}H NMR spectrum of compound 3ea in DMSO-d_6

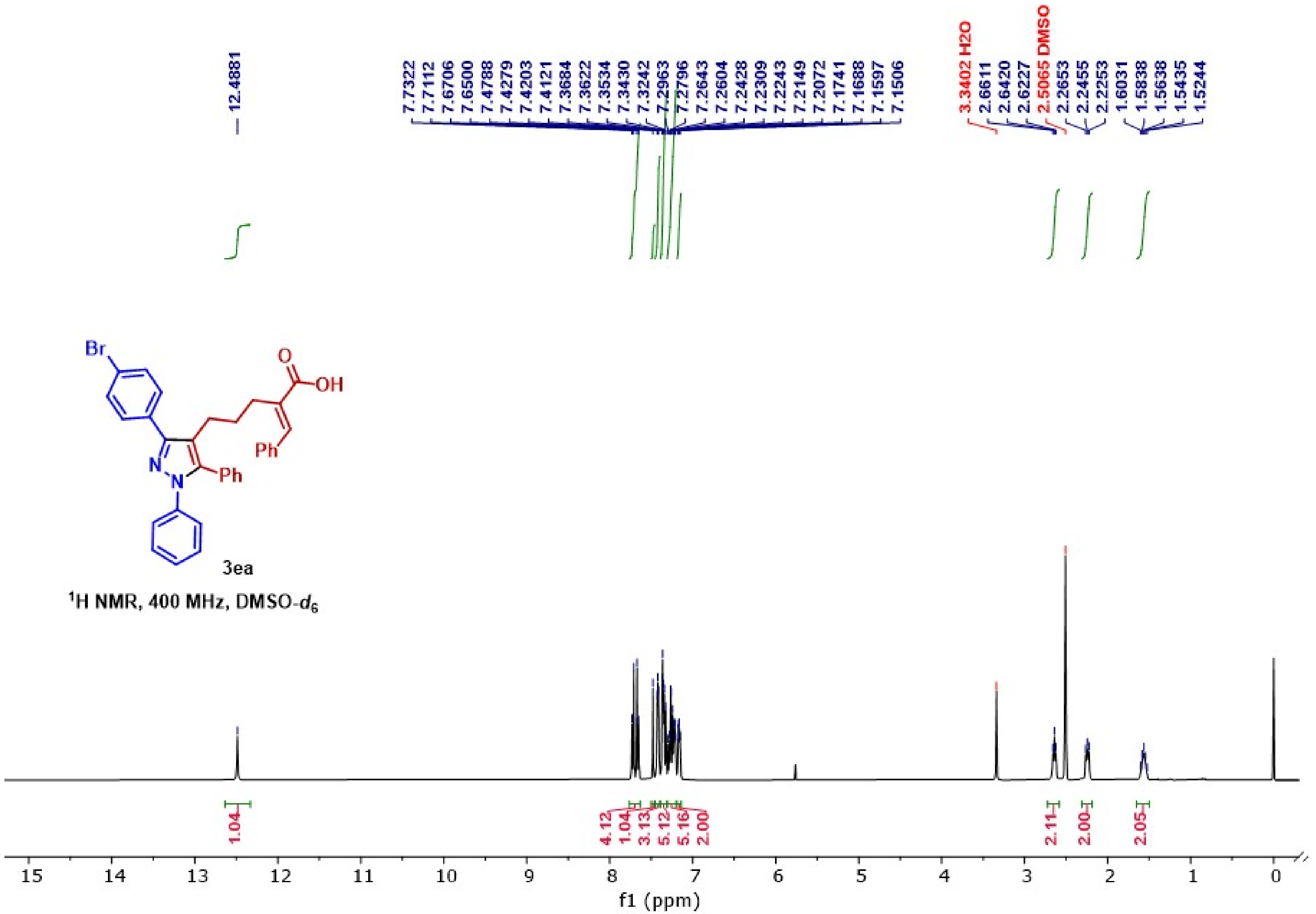

$^{13}C\{^{1}H\}$ **NMR spectrum of compound 3ea in DMSO-d$_6$**

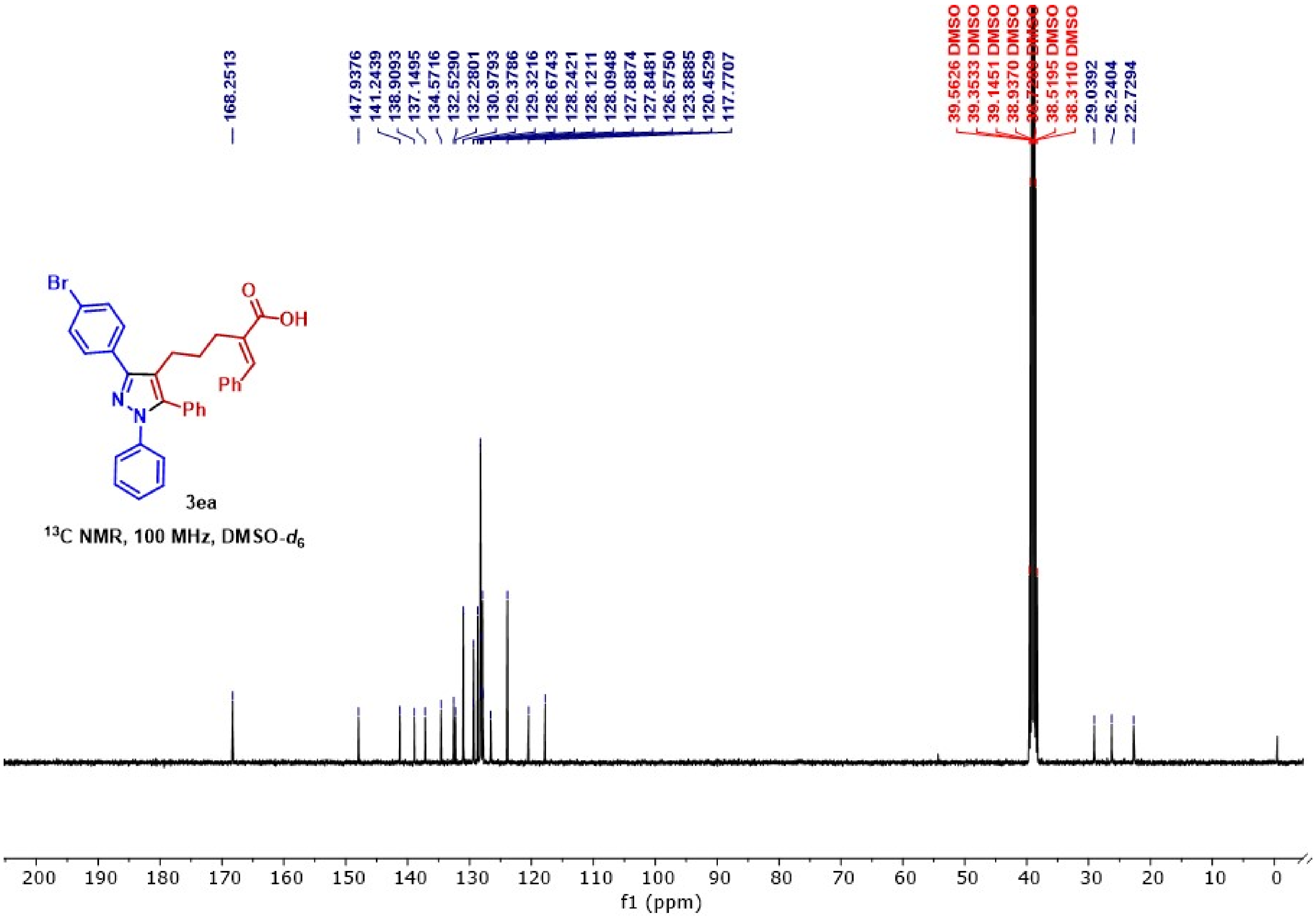

^{1}H NMR spectrum of compound 3ab in DMSO-d_6

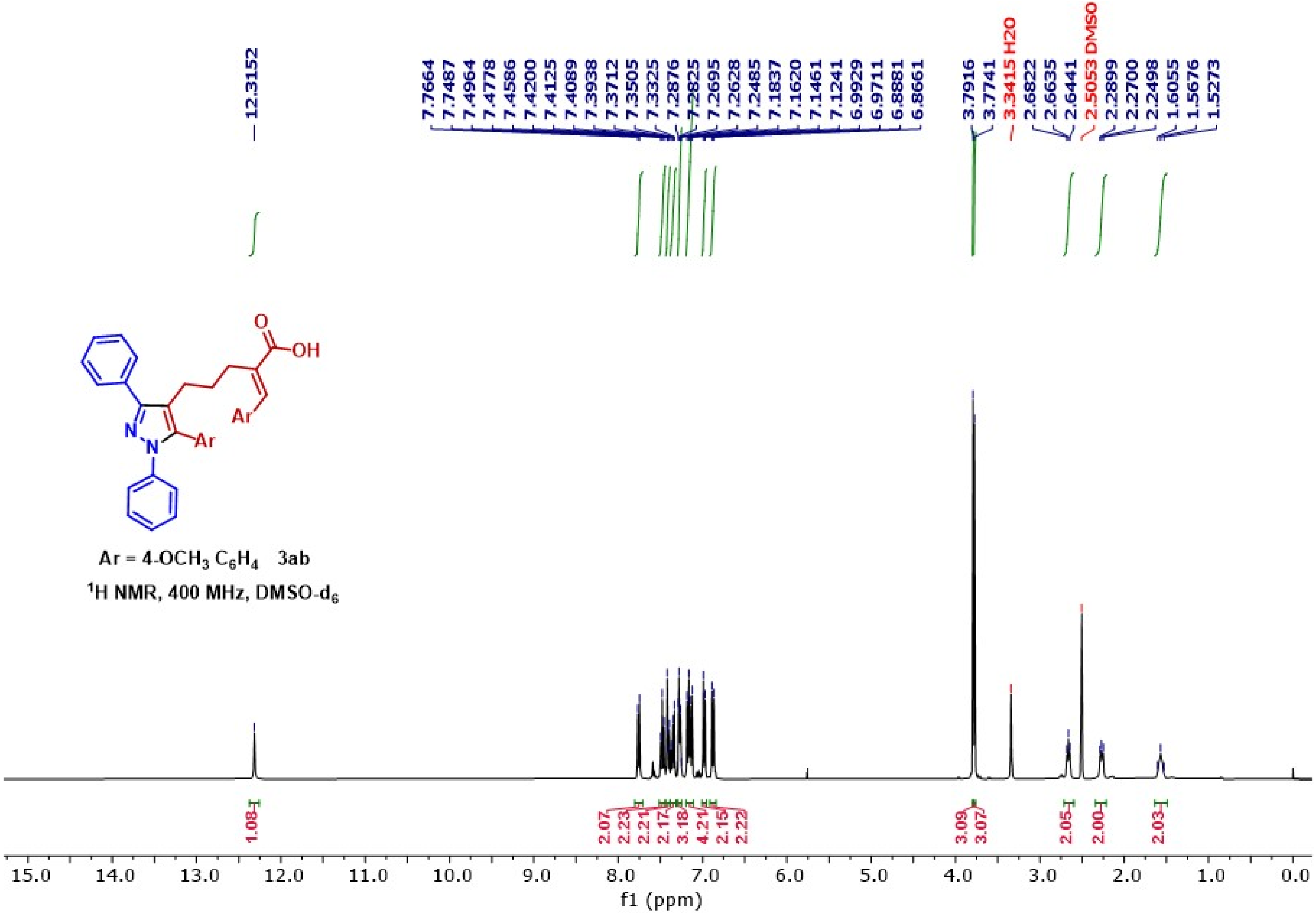

^{13}C{^{1}H} NMR spectrum of compound 3ab in DMSO-d_6

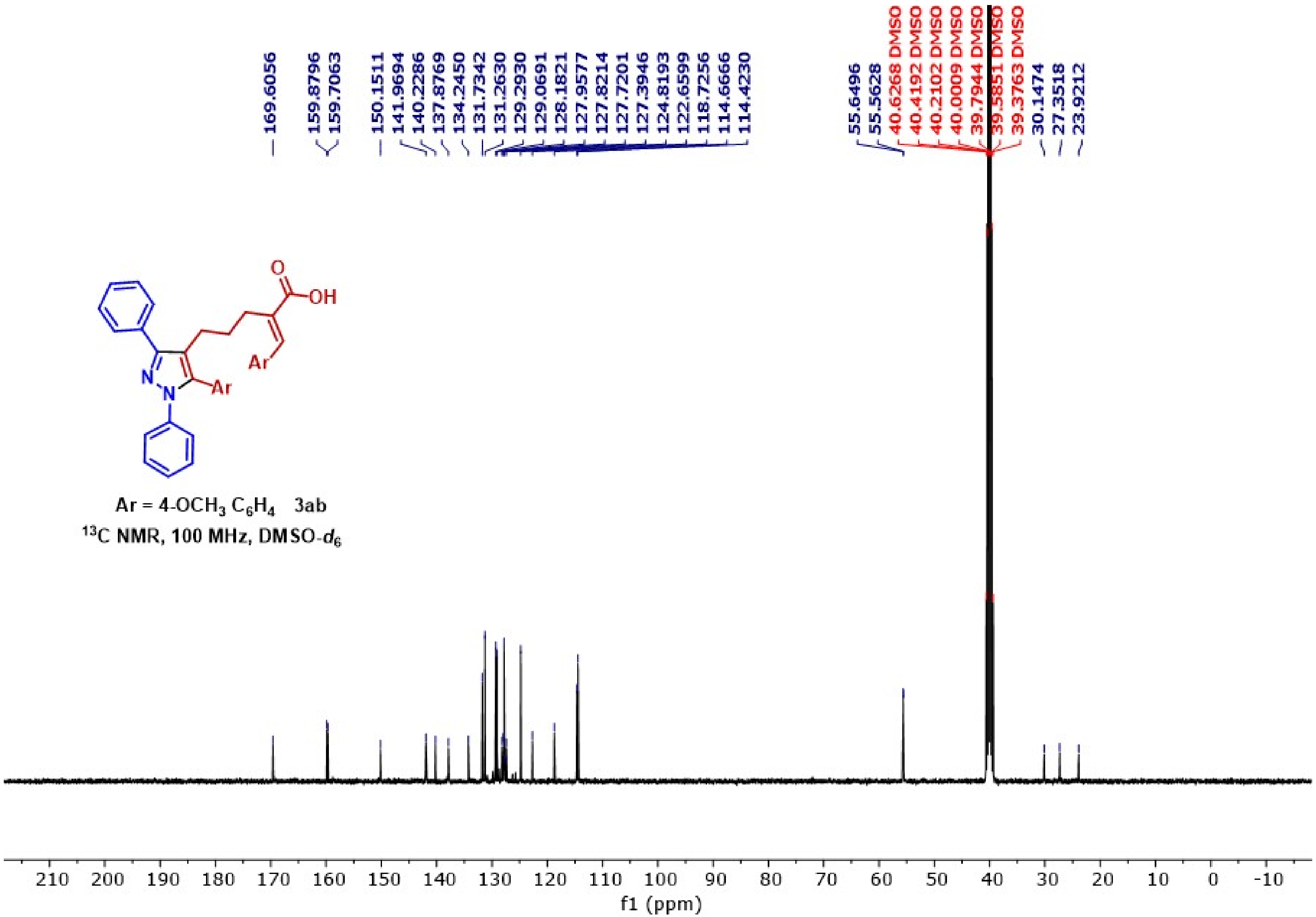

^{1}H NMR spectrum of compound 3ac in DMSO-d$_6$

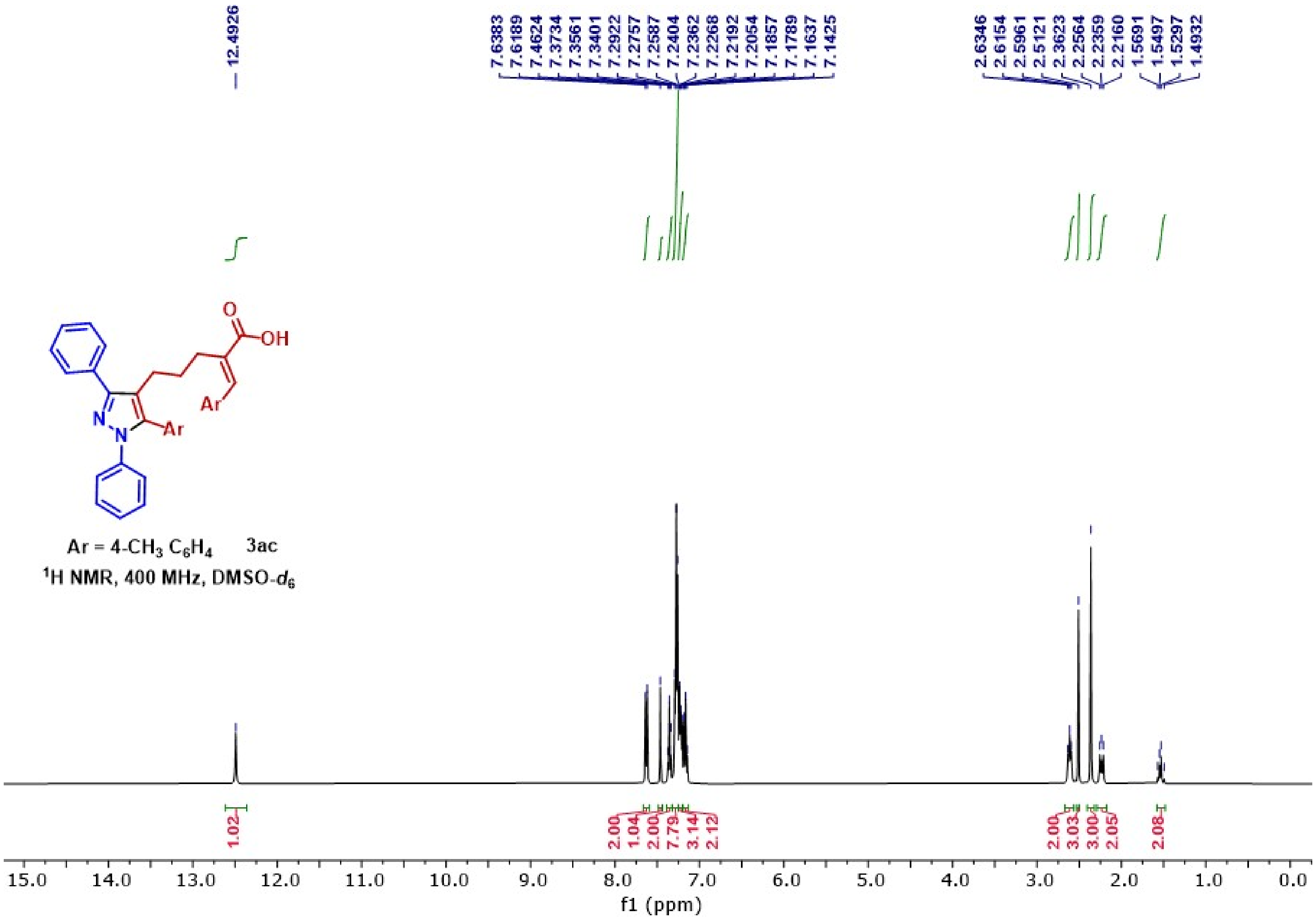

$^{13}C\{^1H\}$ NMR spectrum of compound 3ac in DMSO-d_6

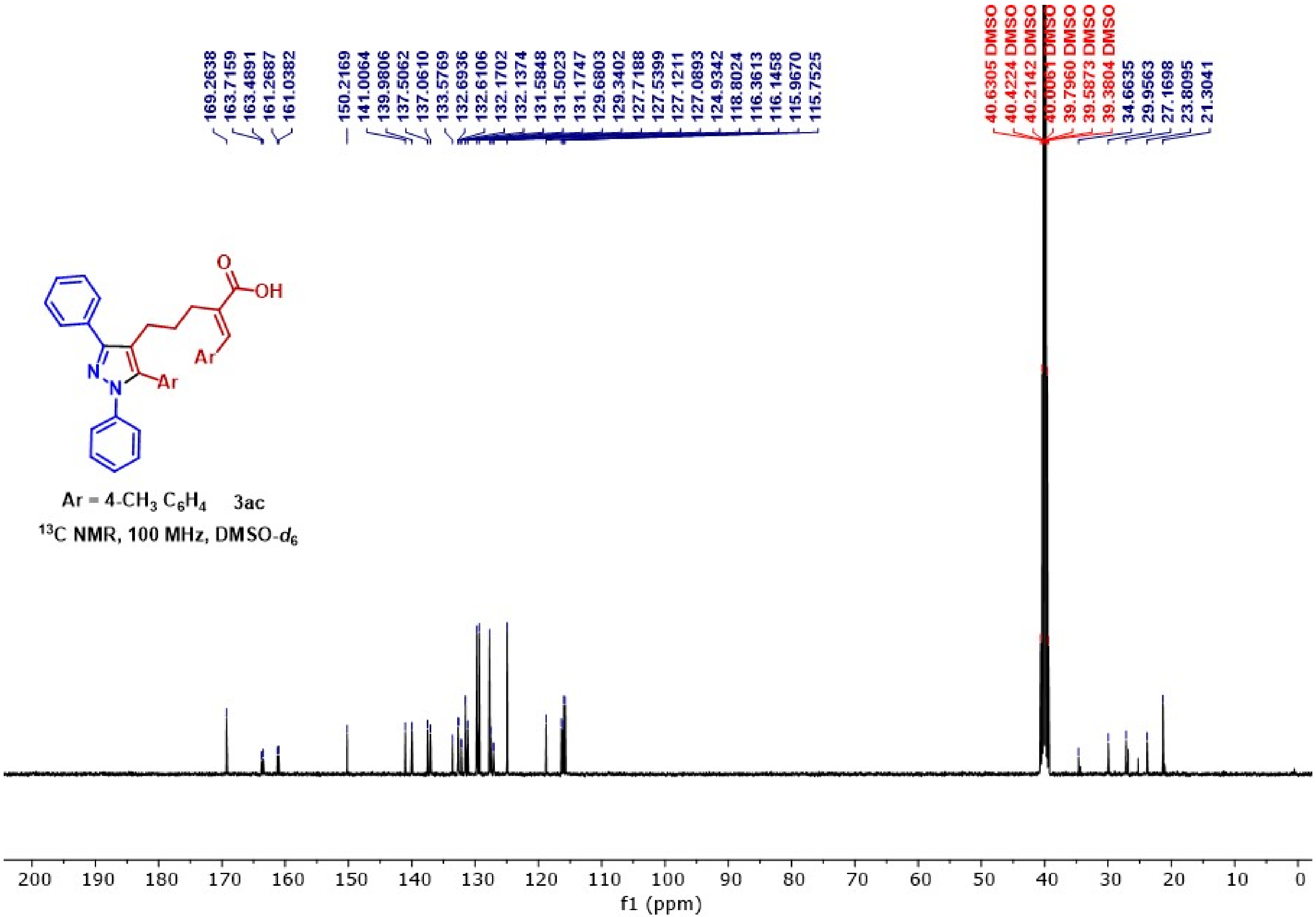

^{1}H NMR spectrum of compound 3ad in DMSO-d_6

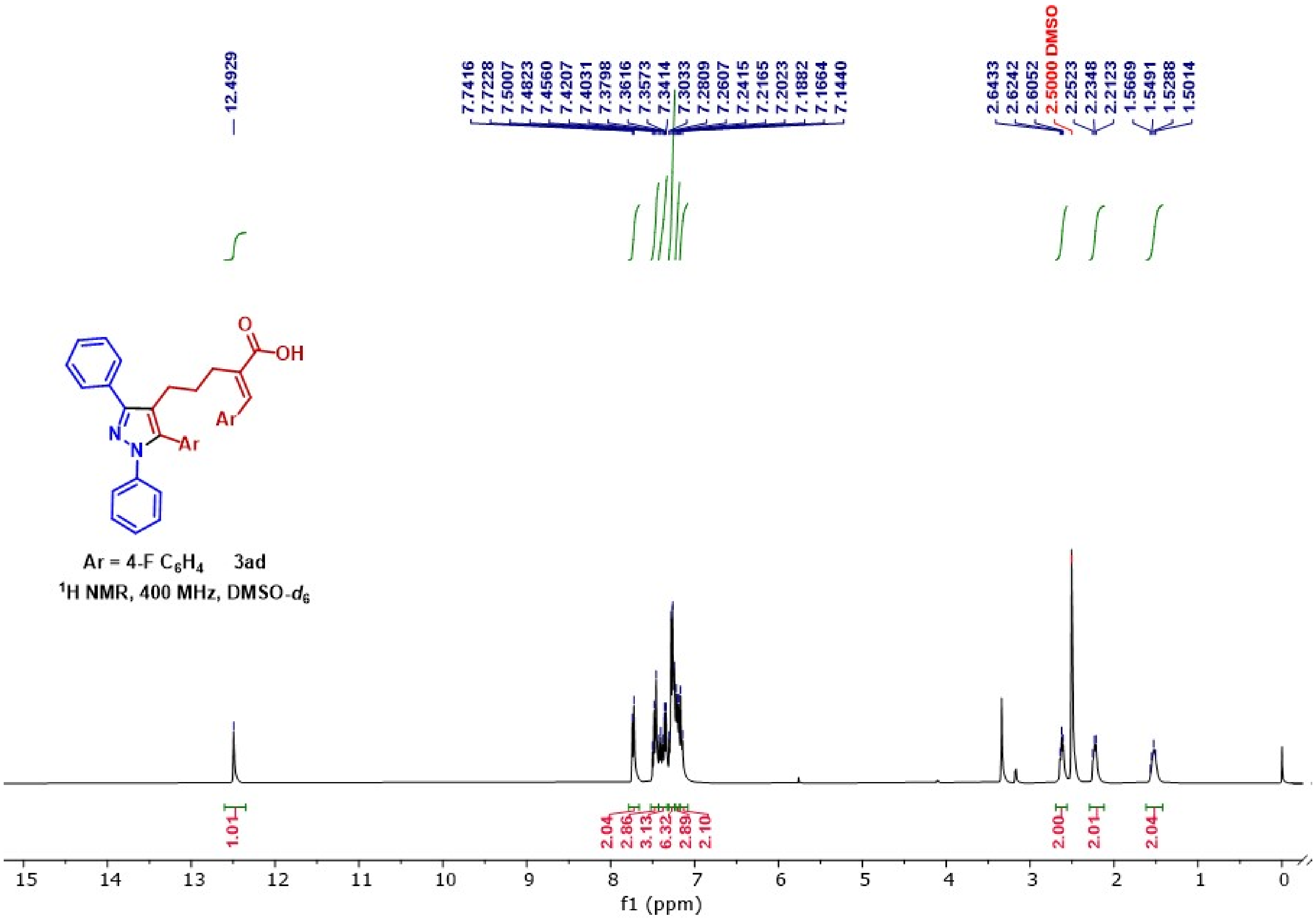

$^{13}C\{^1H\}$ NMR spectrum of compound 3ad in DMSO-d_6

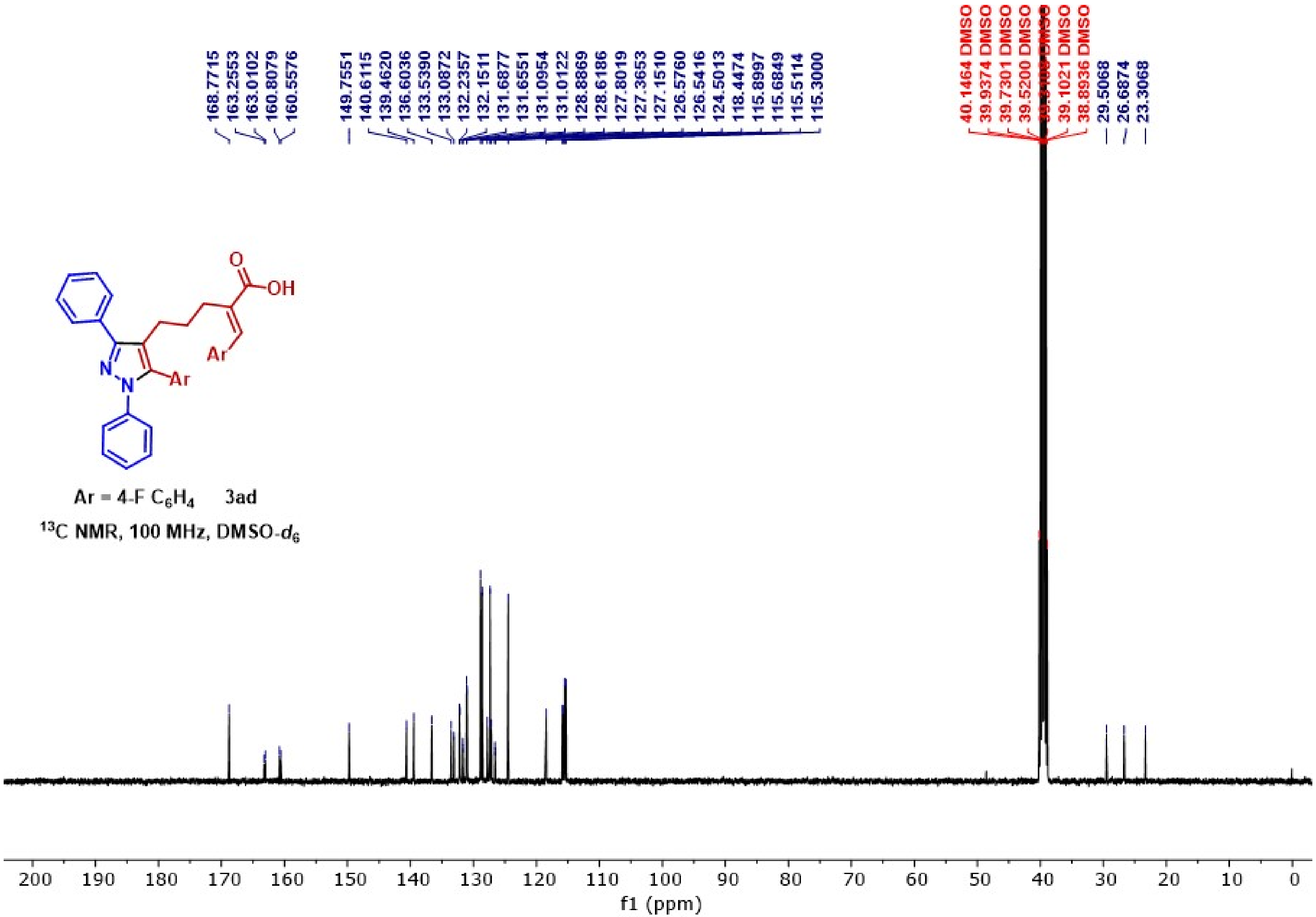

^{1}H NMR spectrum of compound 3fa in DMSO-d_6

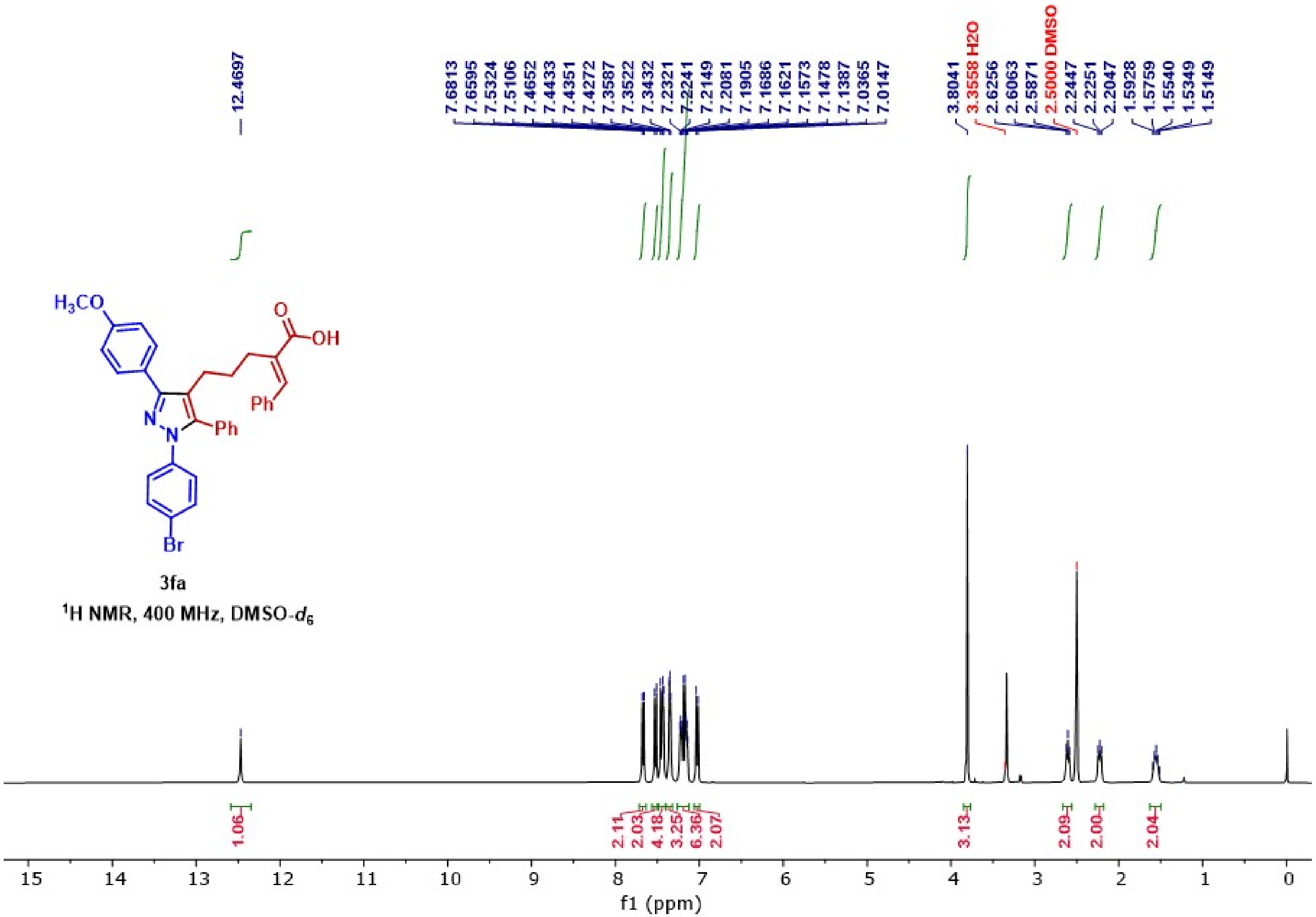

$^{13}C\{^{1}H\}$ NMR spectrum of compound 3fa in DMSO-d_6

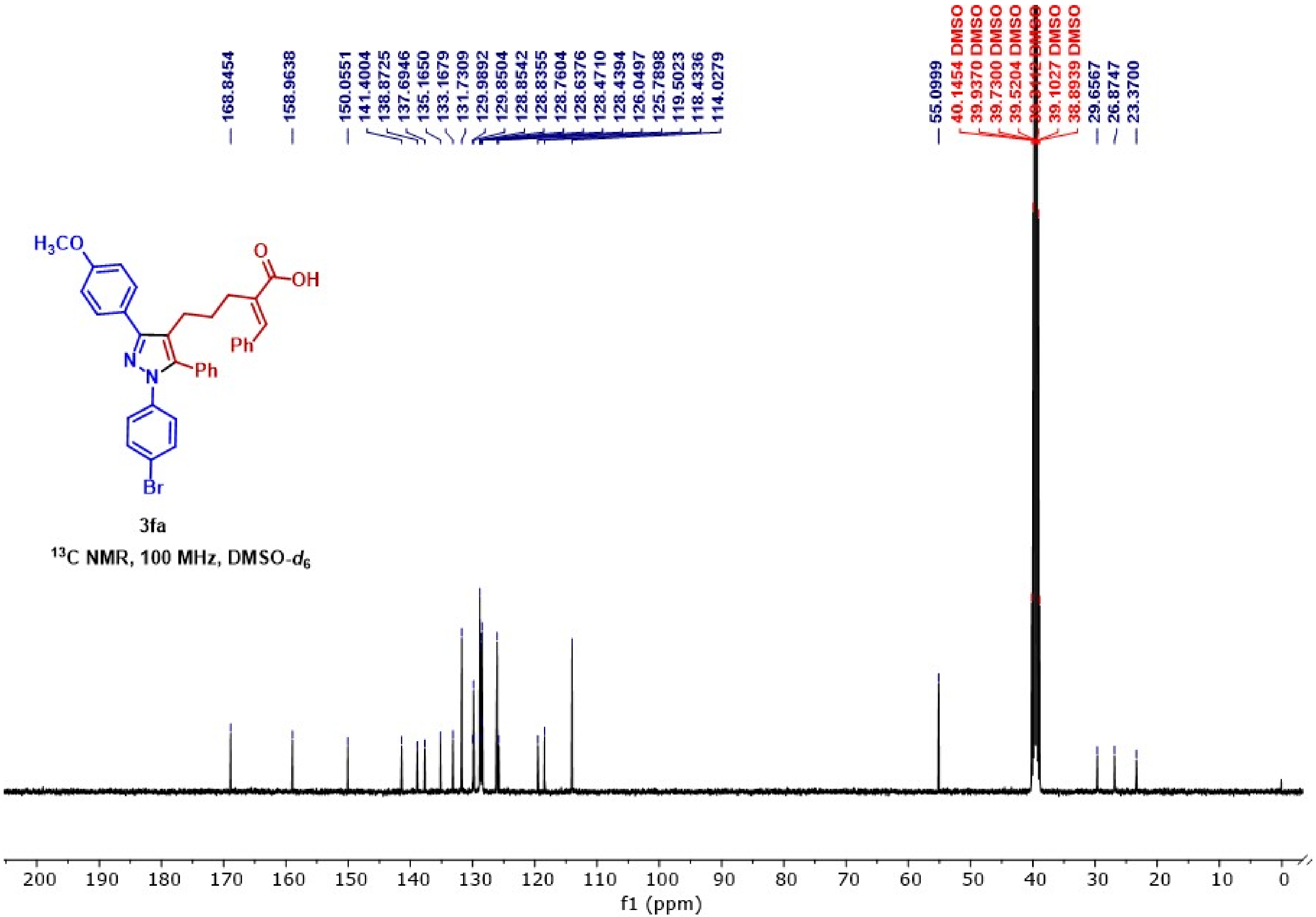

^{1}H NMR spectrum of compound 5aa in DMSO-d_6

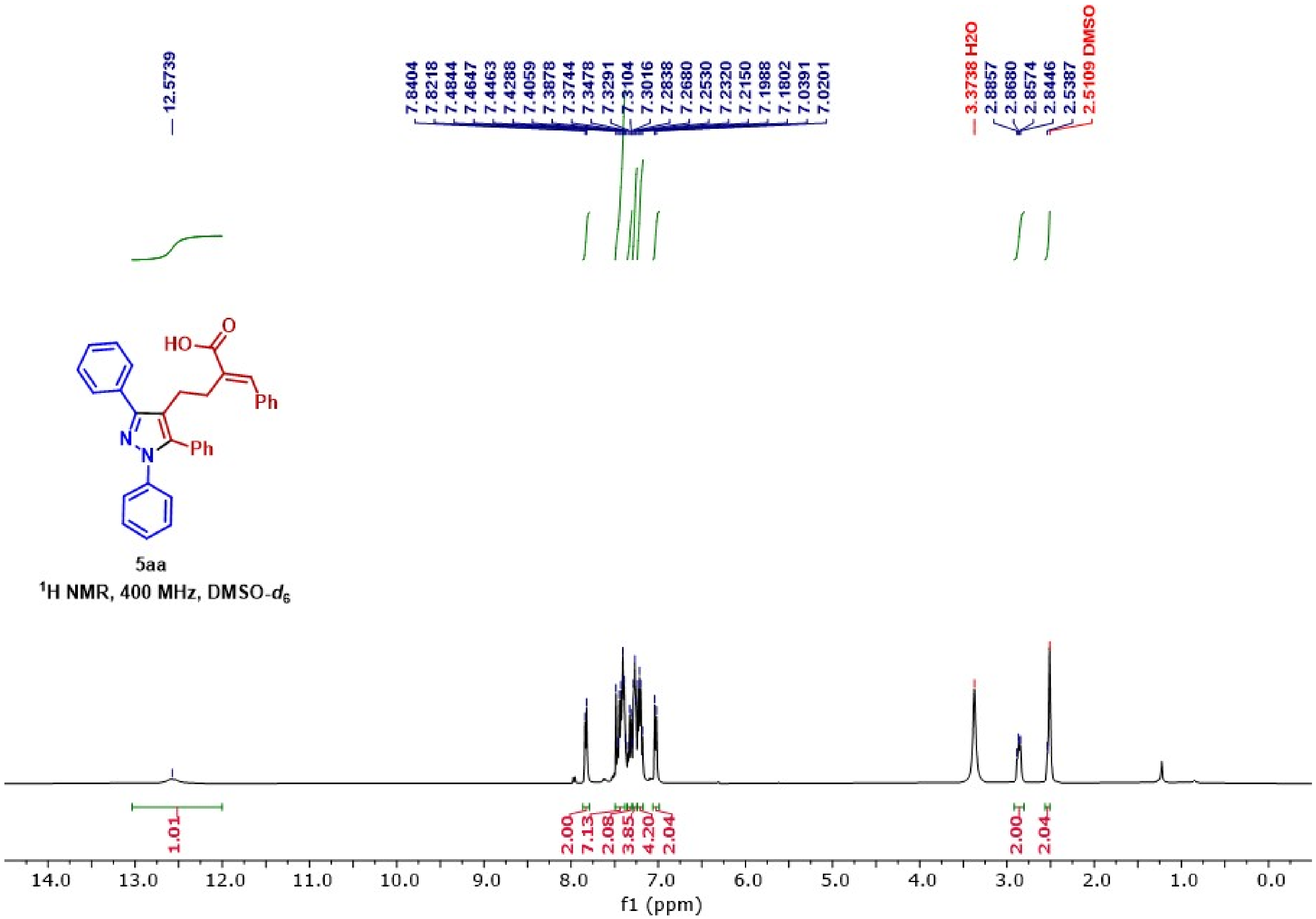

$^{13}C\{^{1}H\}$ NMR spectrum of compound 5aa in DMSO-d_6

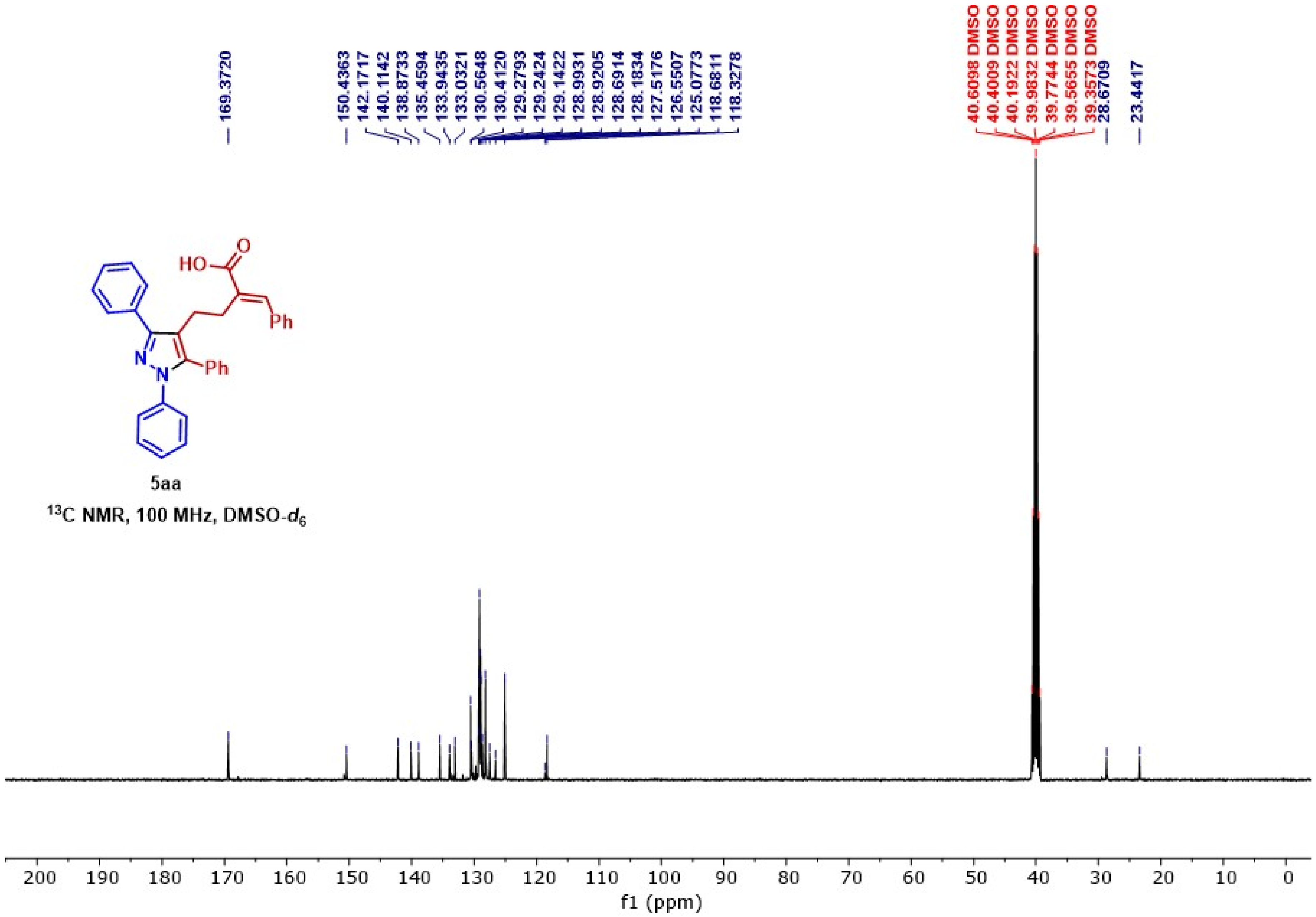

¹H NMR spectrum of compound 5ba in DMSO-d_6

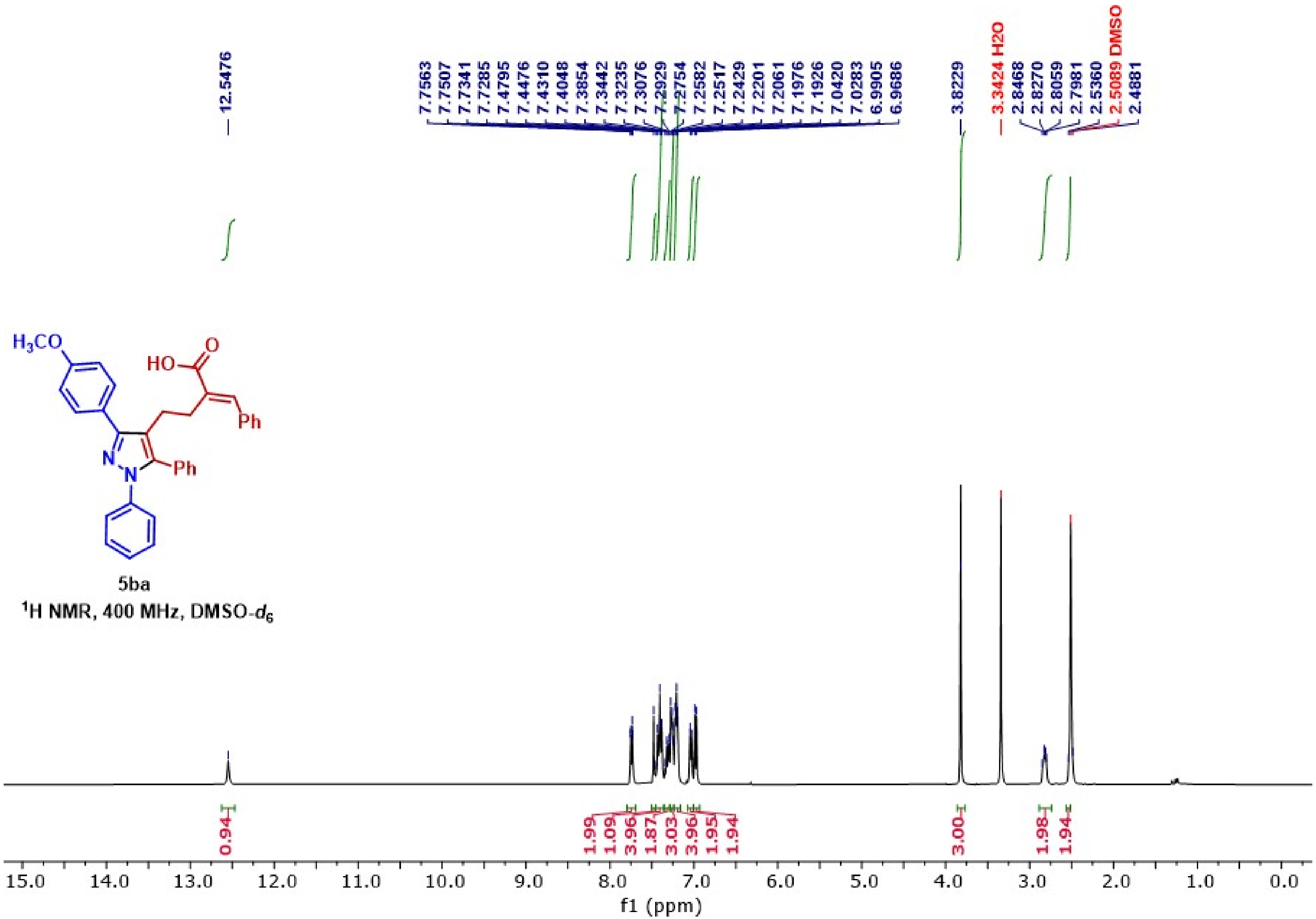

$^{13}C\{^1H\}$ NMR spectrum of compound 5ba in DMSO-d_6

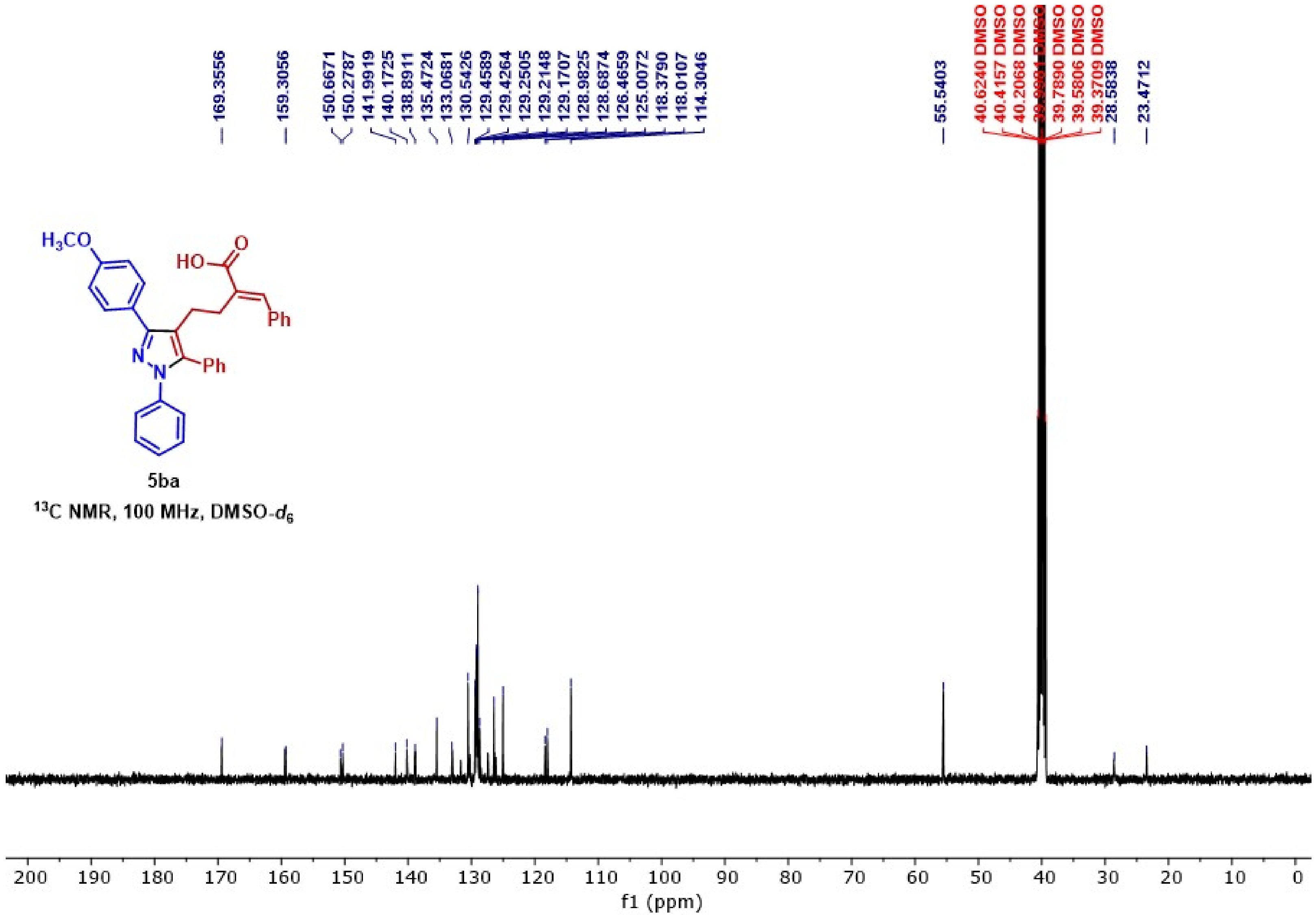

^{1}H NMR spectrum of compound 5ca in DMSO-d_6

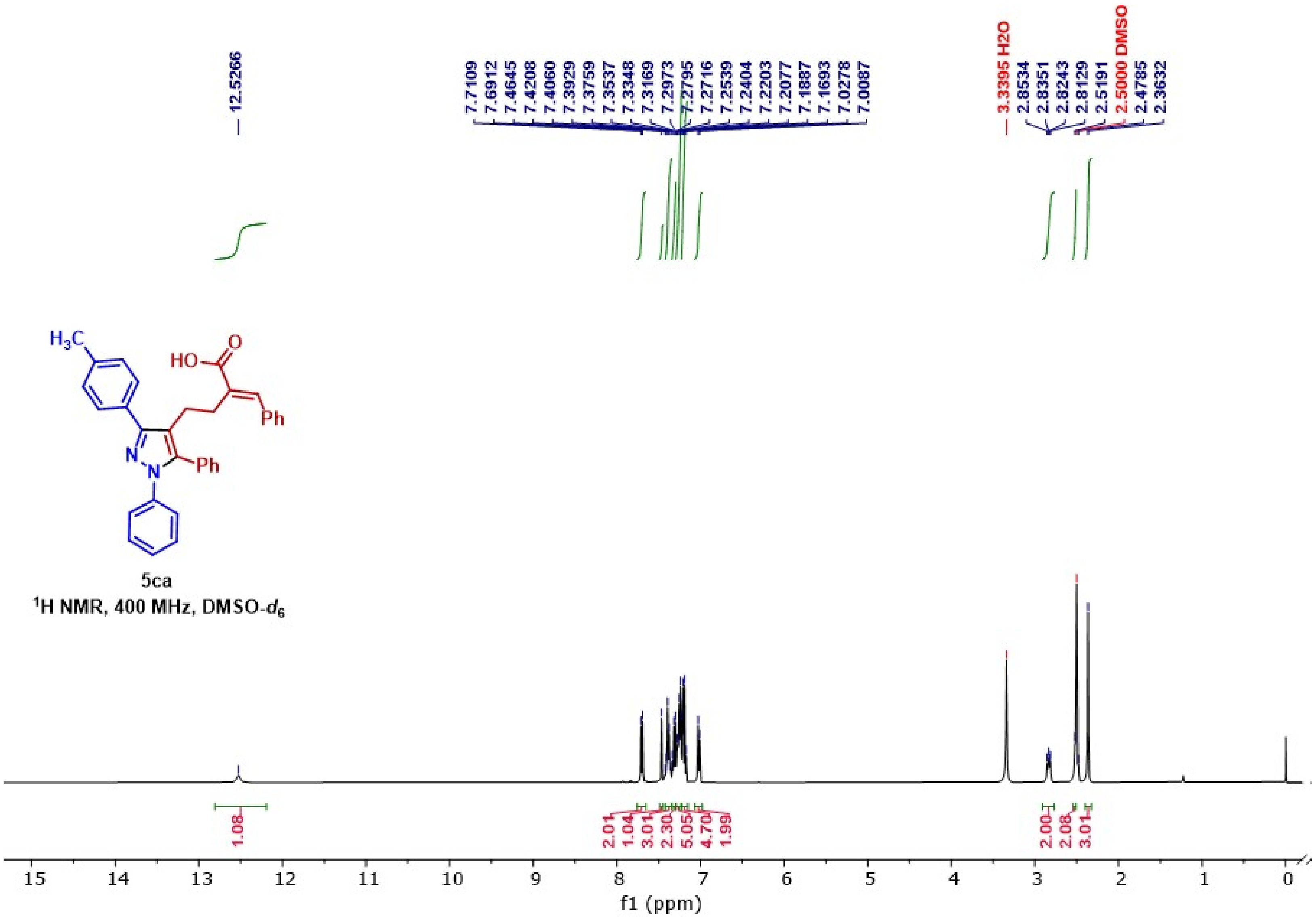

$^{13}C\{^{1}H\}$ **NMR spectrum of compound 5ca in DMSO-d$_6$**

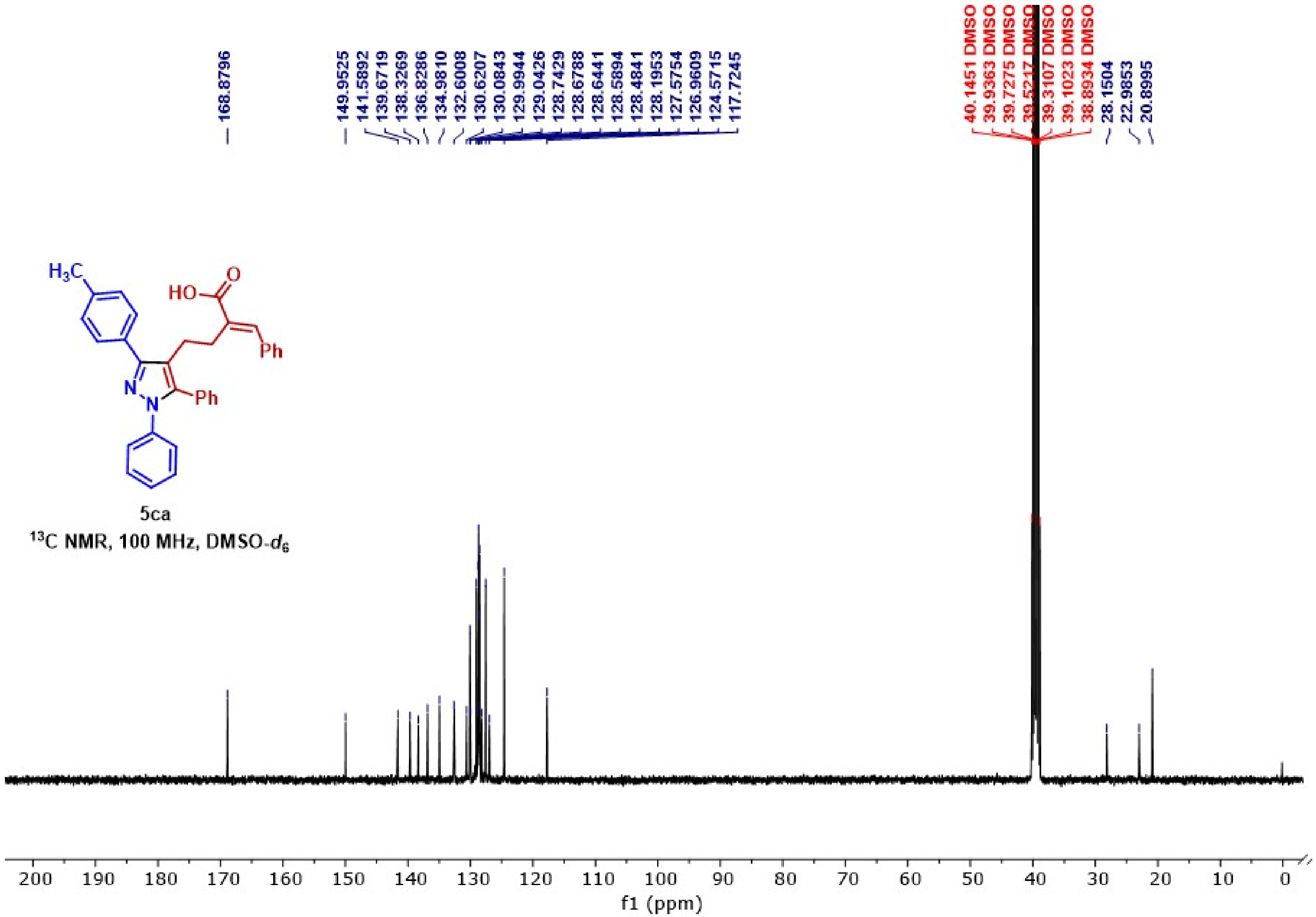

^{1}H NMR spectrum of compound 5da in DMSO-d_6

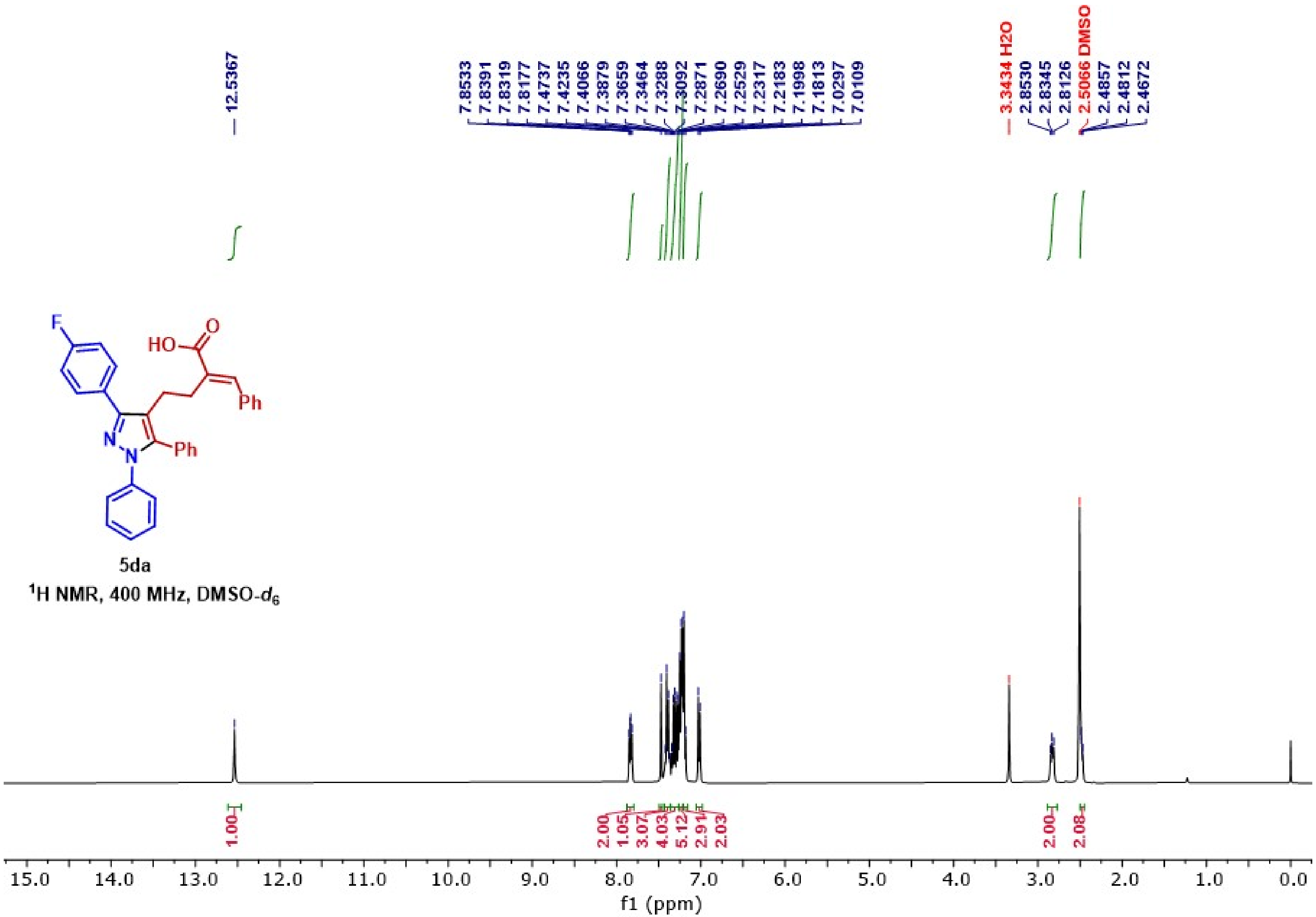

$^{13}C\{^{1}H\}$ NMR spectrum of compound 5da in DMSO-d_6

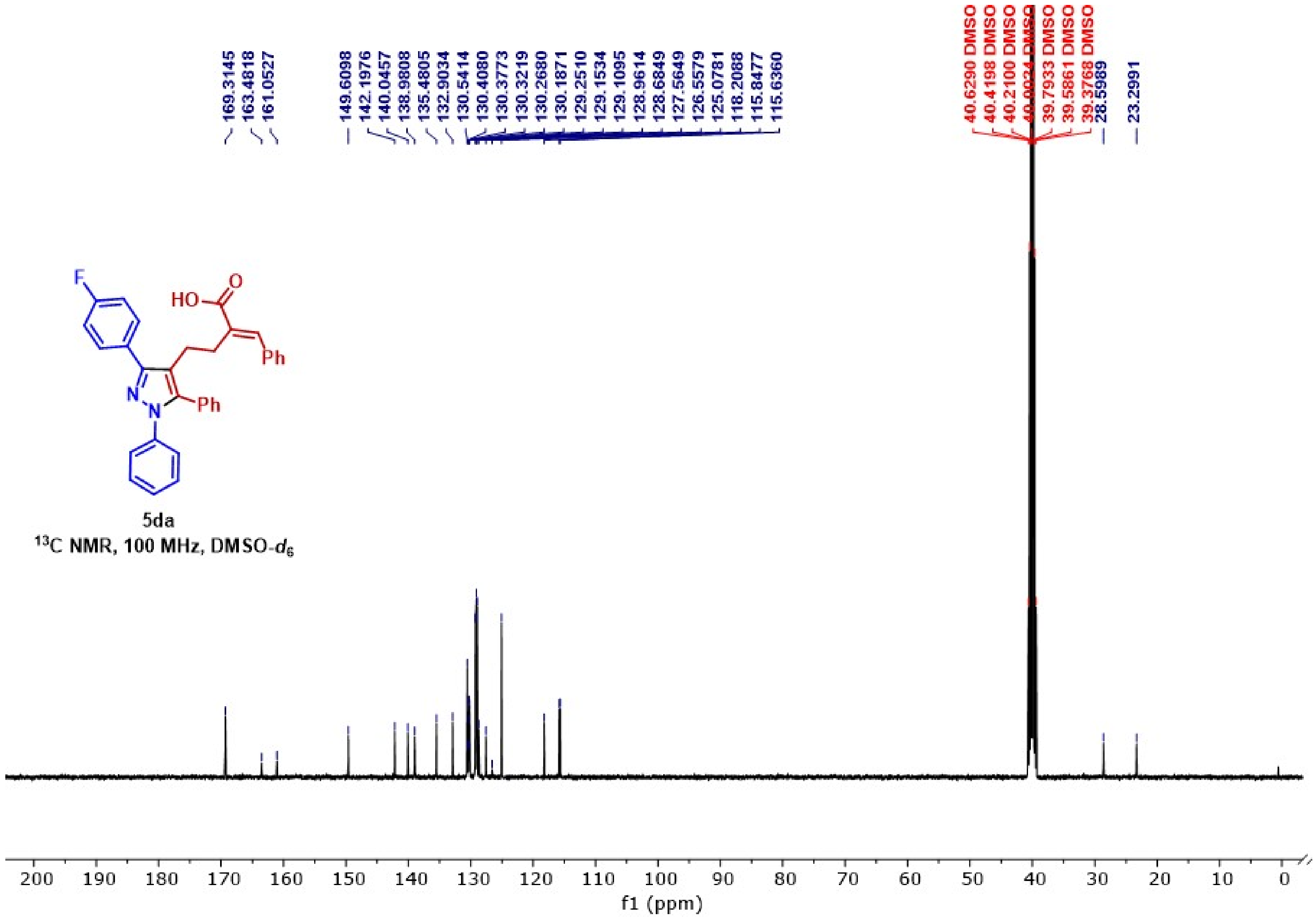

^{1}H NMR spectrum of compound 5ea in DMSO-d_6

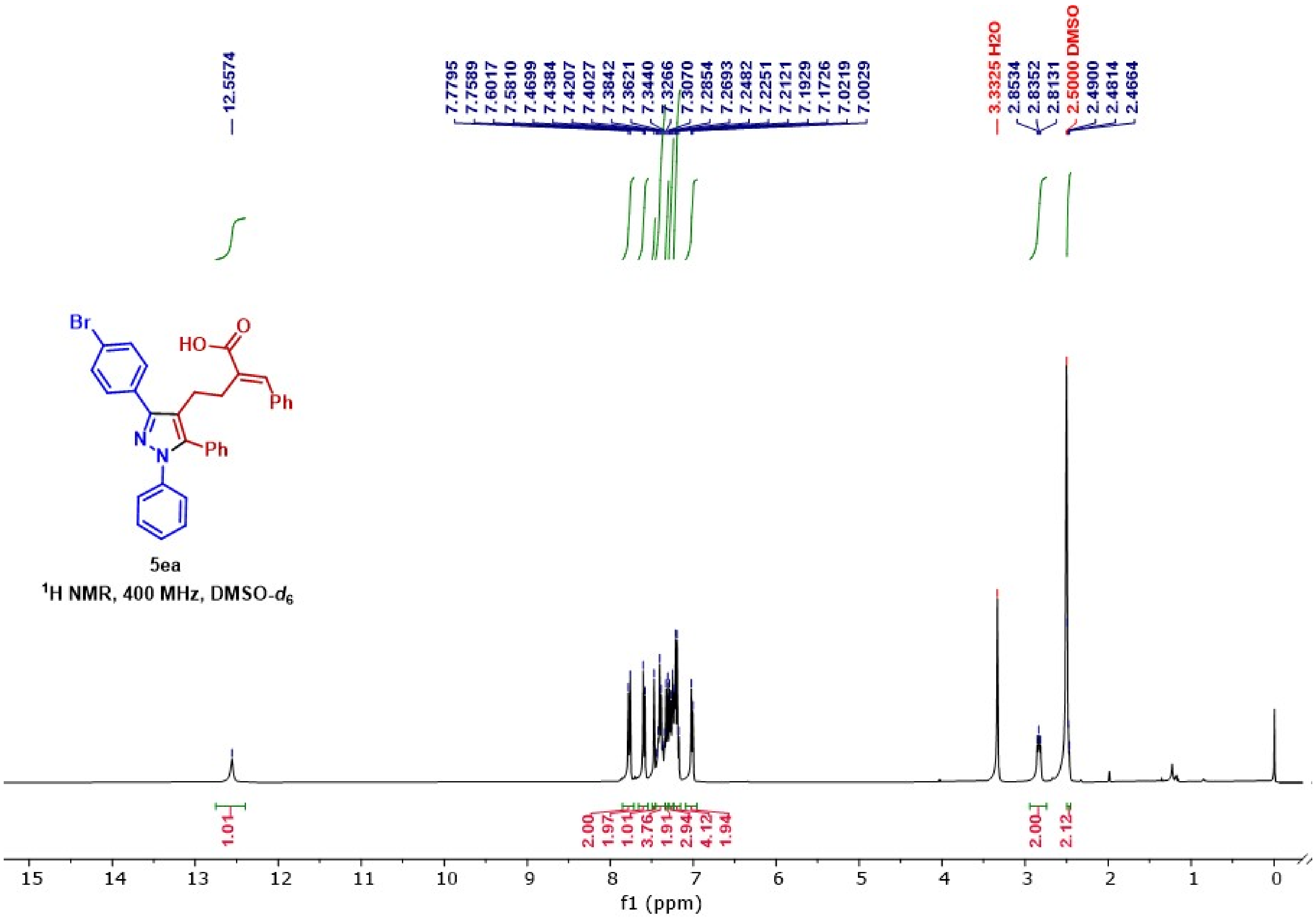

$^{13}C\{^{1}H\}$ NMR spectrum of compound 5ea in DMSO-d_6

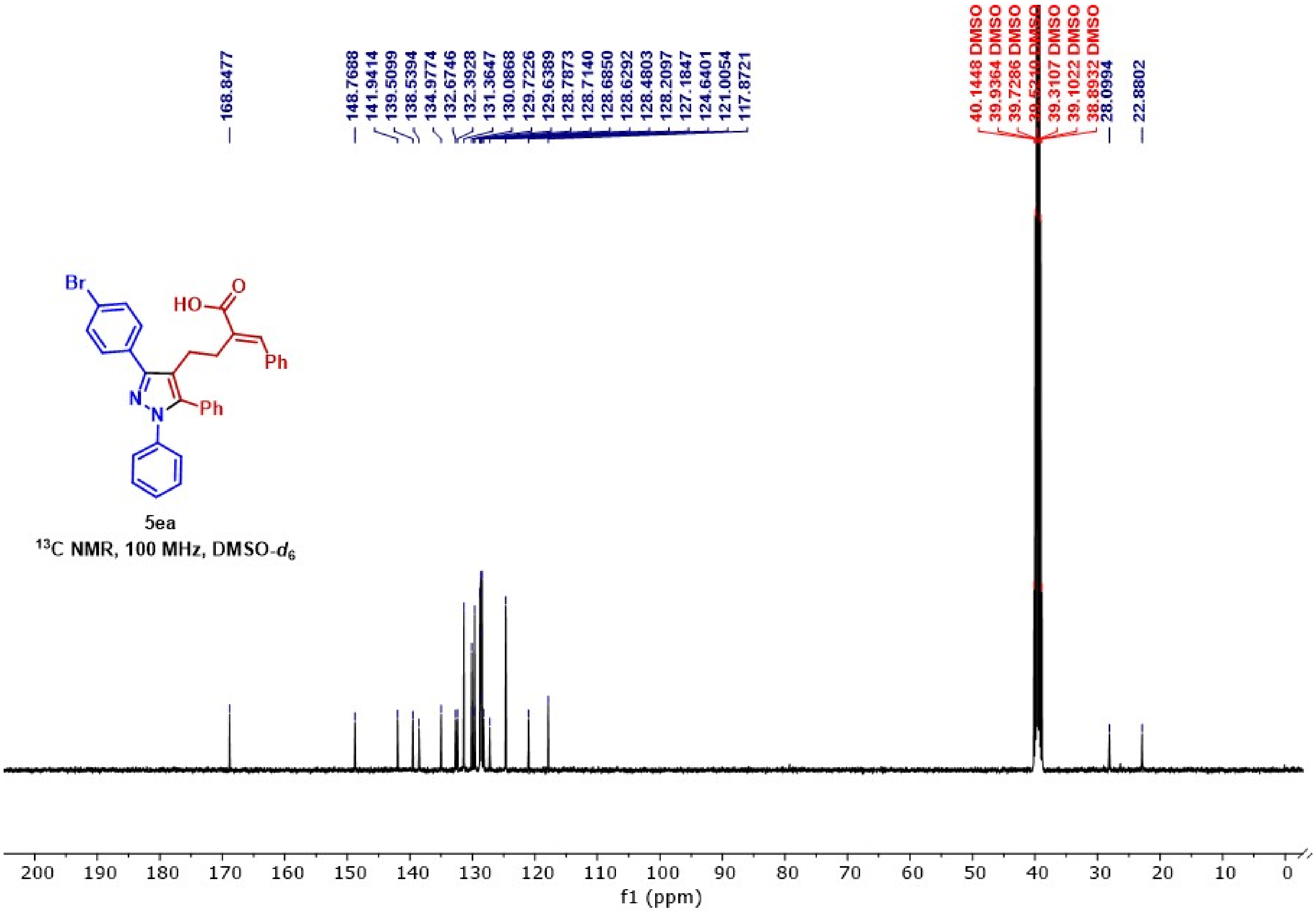

^{1}H NMR spectrum of compound 3fa in DMSO-d_6

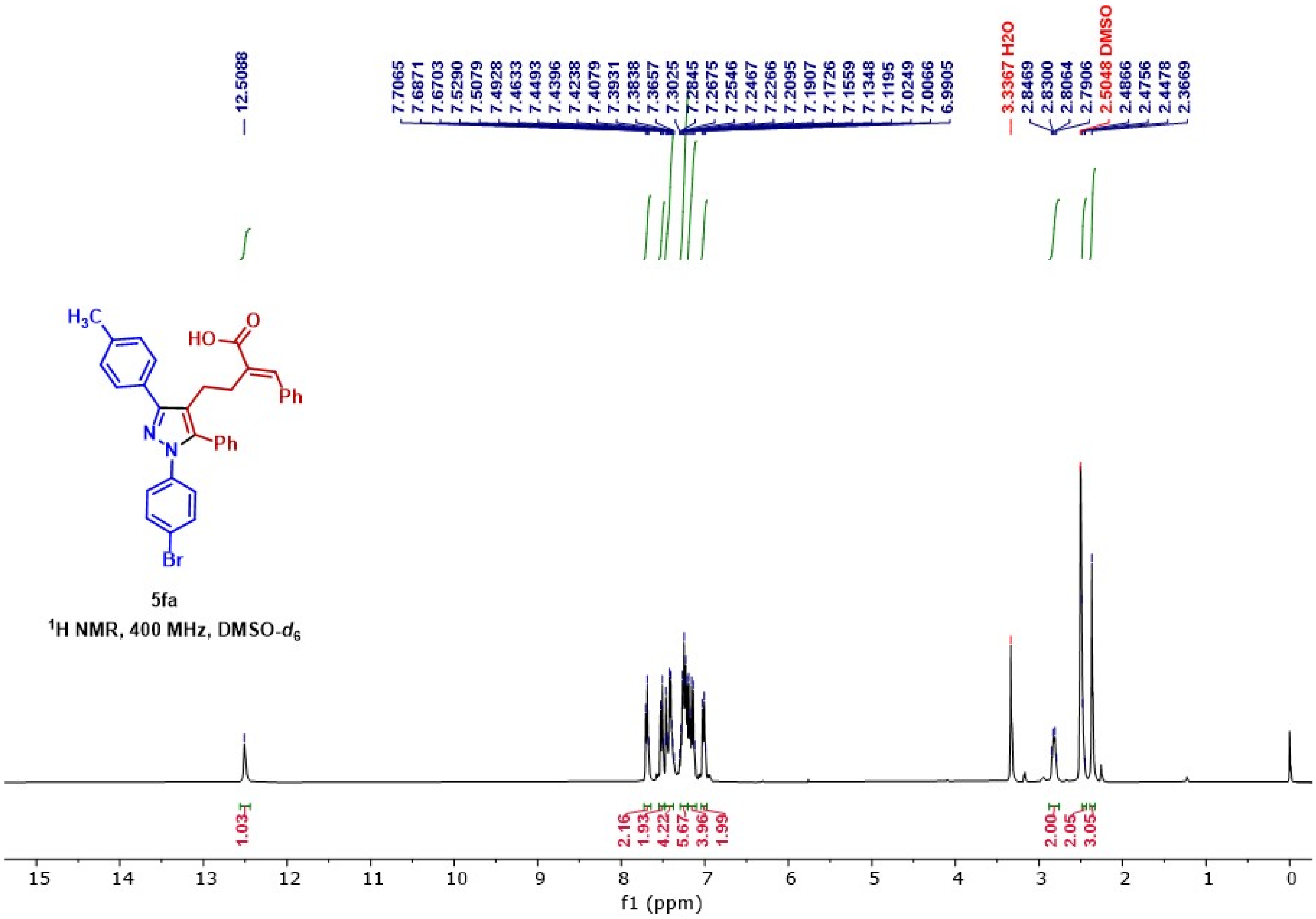

$^{13}C\{^{1}H\}$ spectrum NMR of compound 5fa in DMSO-d_6

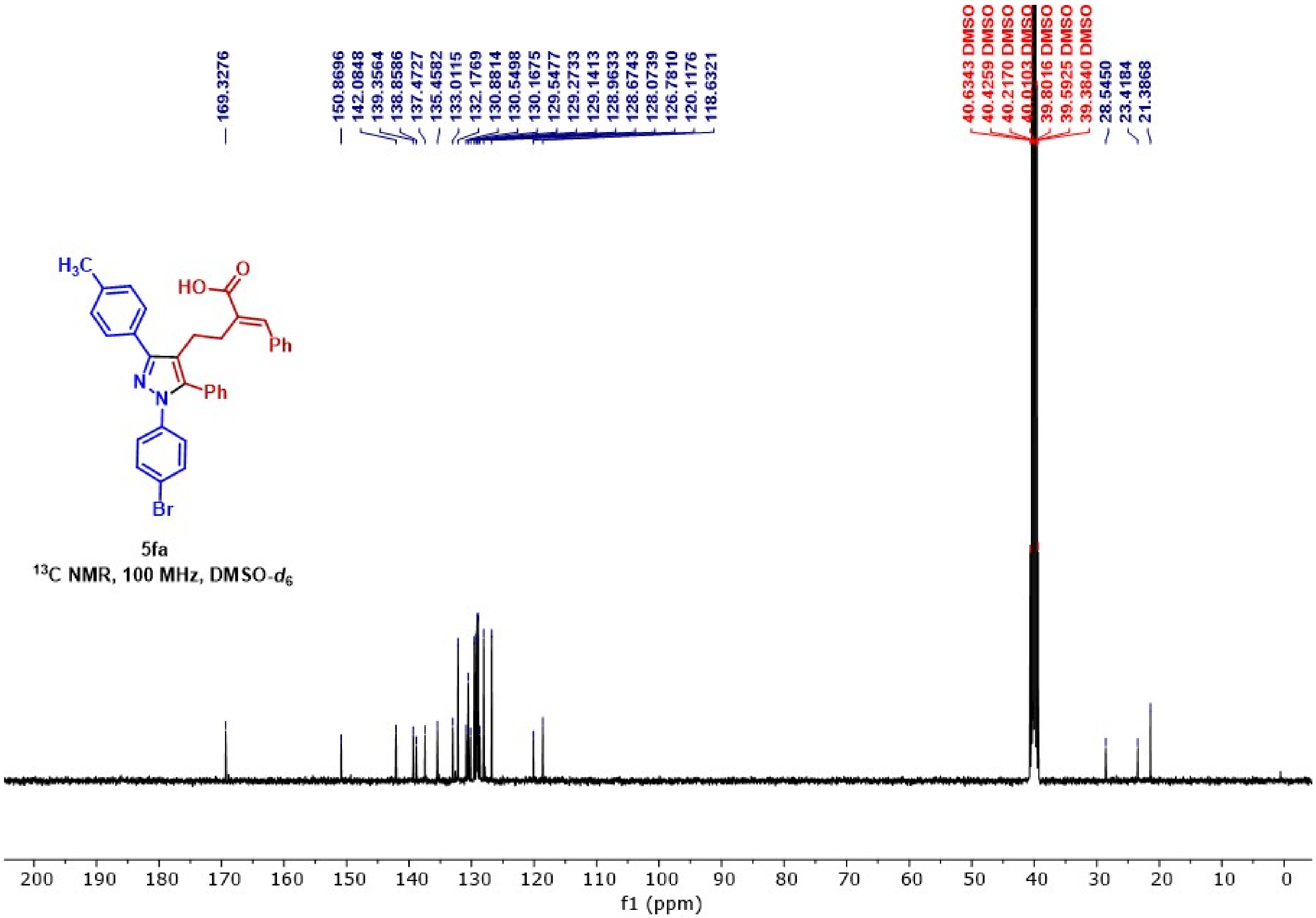

^{1}H NMR spectrum of compound 5ga in DMSO-d_6

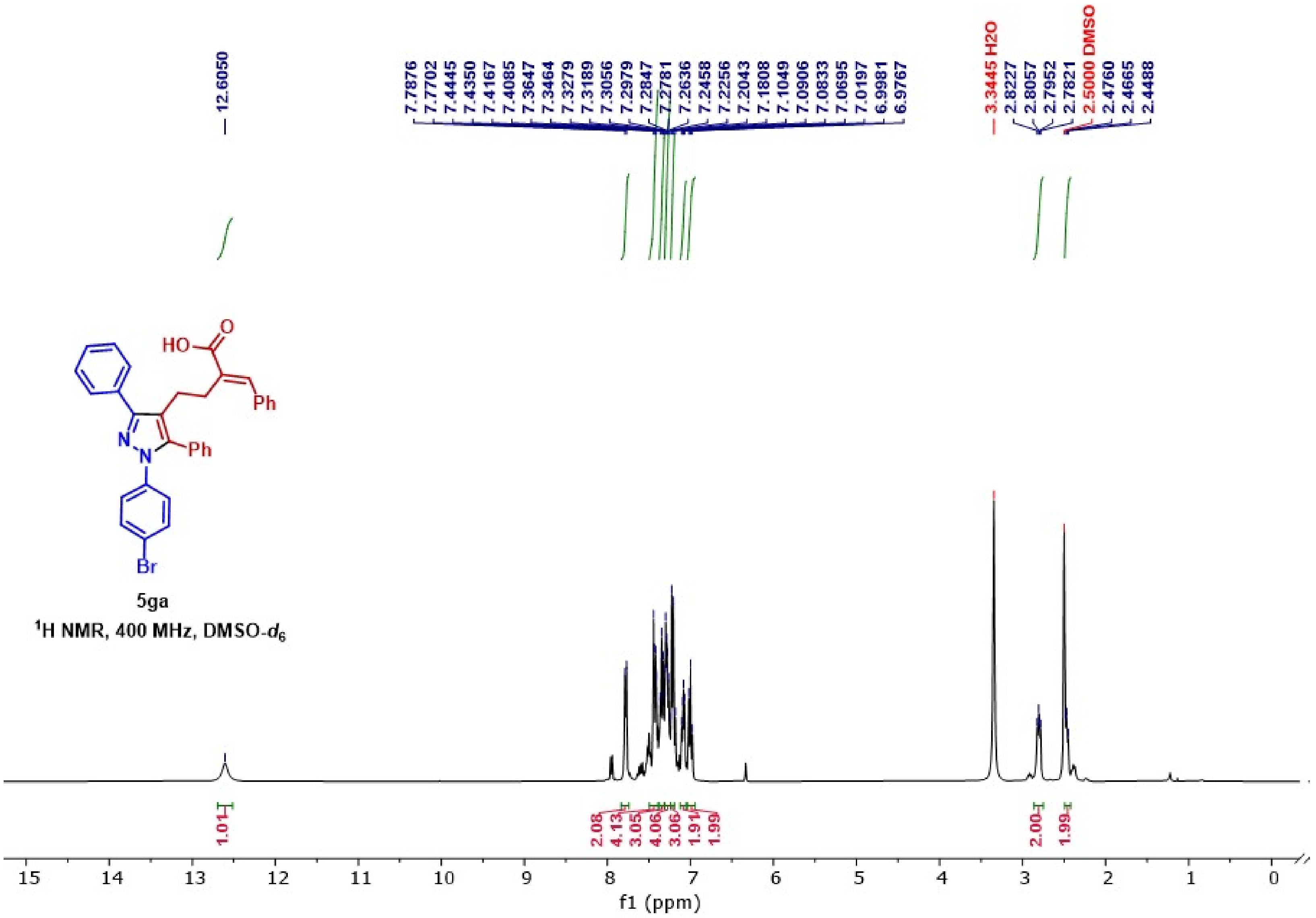

$^{13}C\{^1H\}$ NMR spectrum of compound 5ga in DMSO-d_6

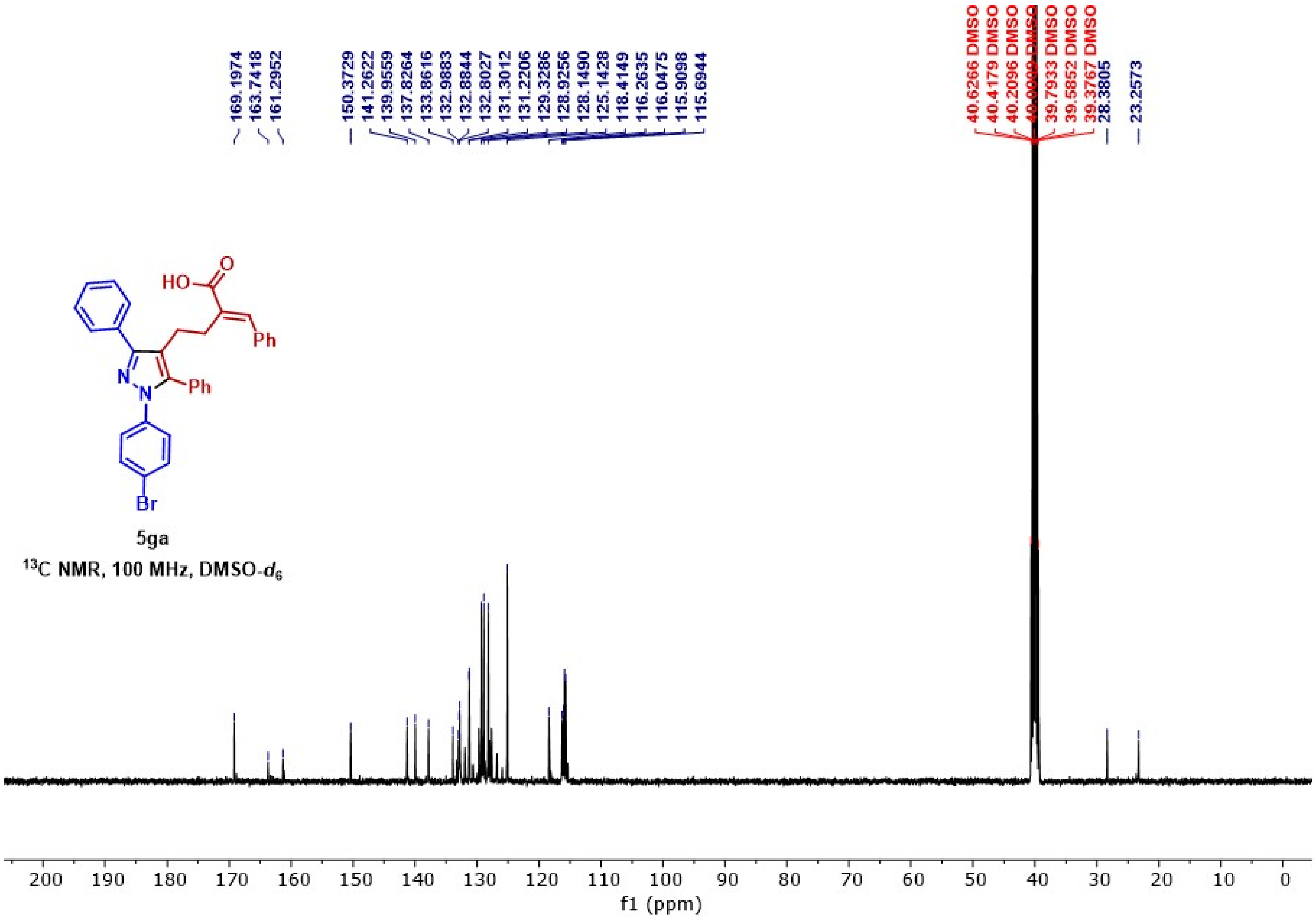

^{1}H NMR spectrum of compound 5ab in DMSO-d_6

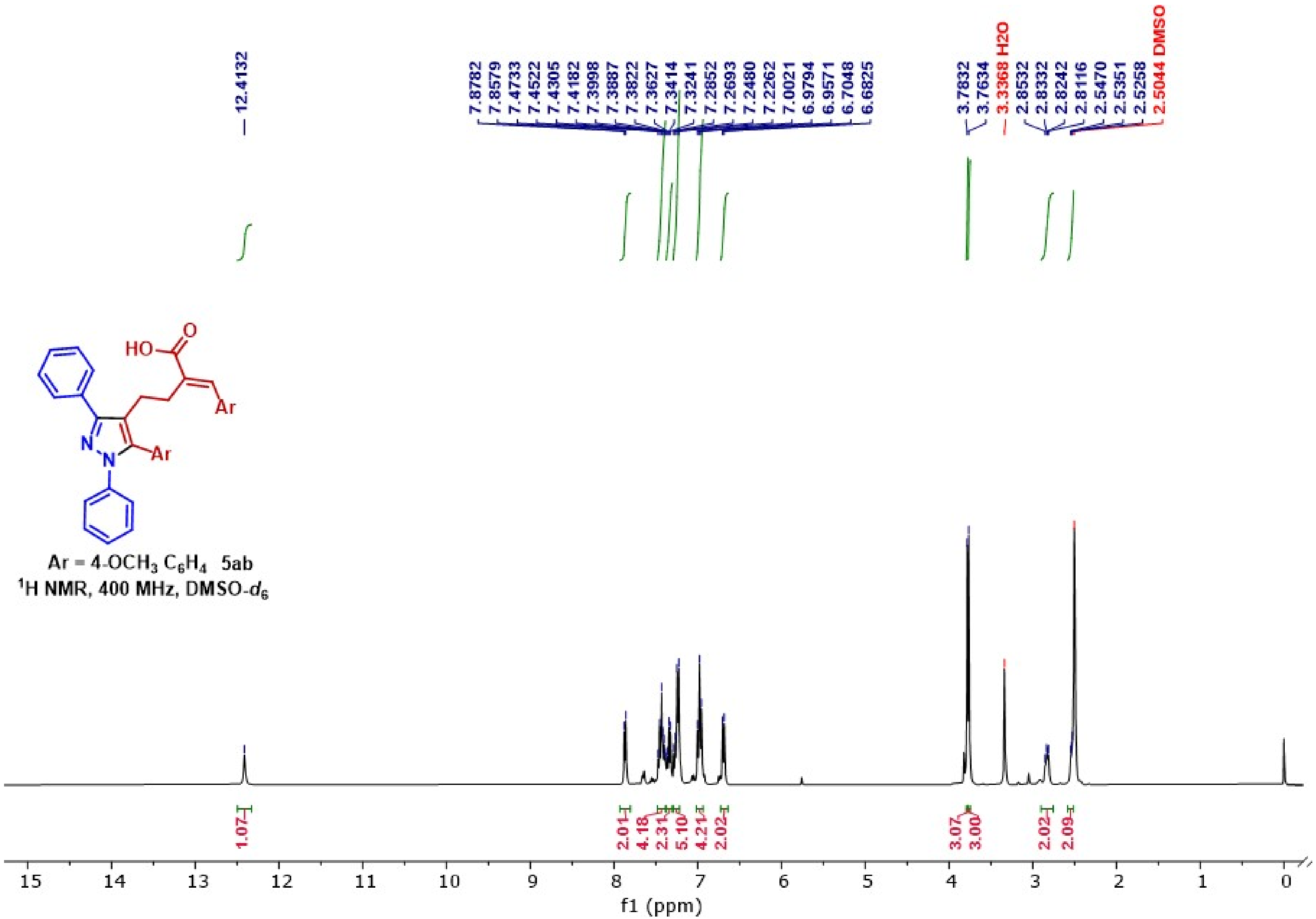

$^{13}C\{^{1}H\}$ NMR spectrum of compound 5ab in DMSO-d_6

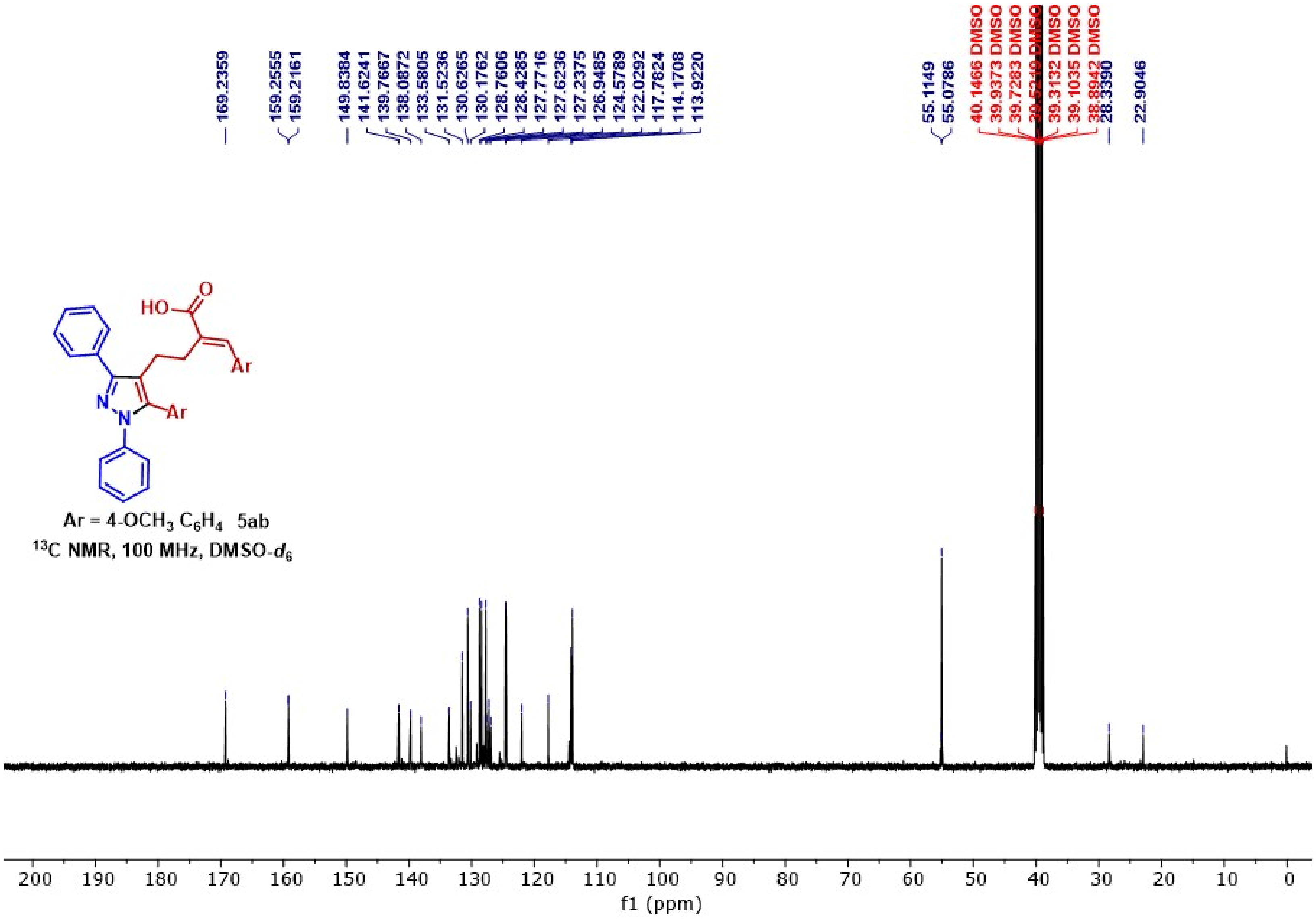

^{1}H NMR spectrum of compound 5ac in DMSO-d_6

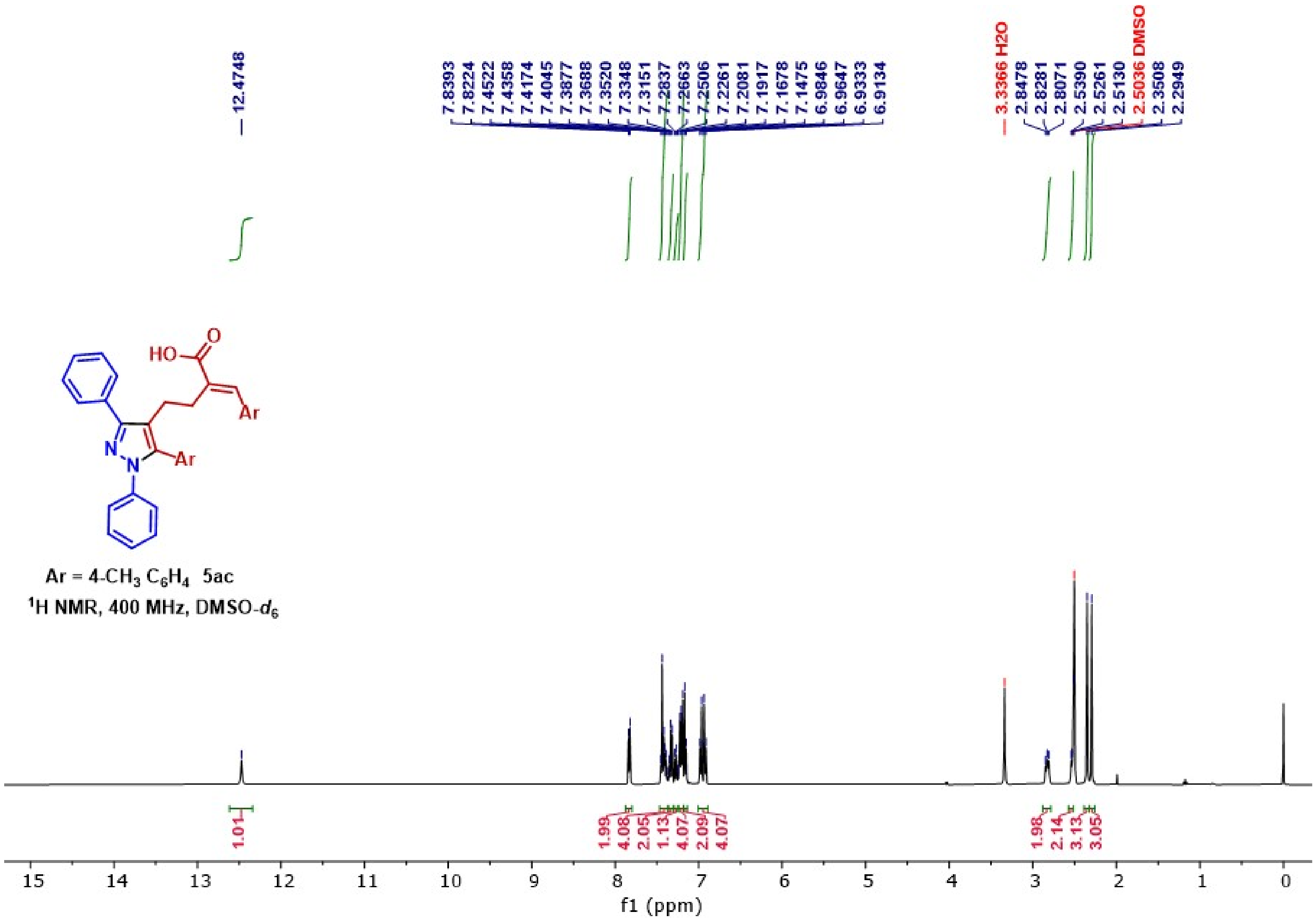

$^{13}C\{^1H\}$ NMR spectrum of compound 5ac in DMSO-d_6

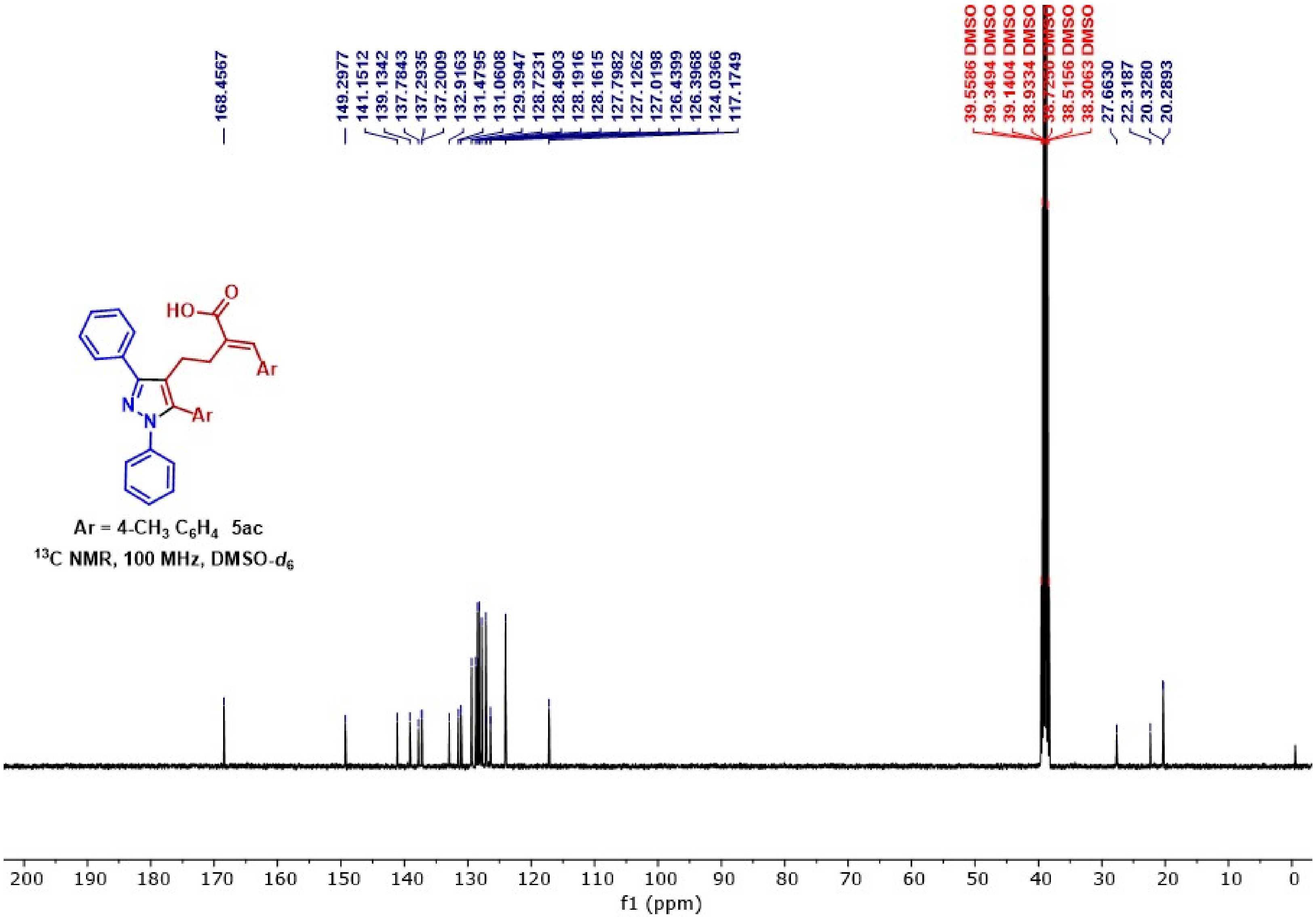

Supplement: RA-015-D5RA03561A-s001 [file RA-015-D5RA03561A-s001.pdf]
